# Supplementary figures and images for: A pulse-chasable reporter processing assay for mammalian autophagic flux with HaloTag
Source: eLife. 2022 Aug 8;11:e78923. doi: 10.7554/eLife.78923 (PMC9385206; doi:10.7554/eLife.78923)

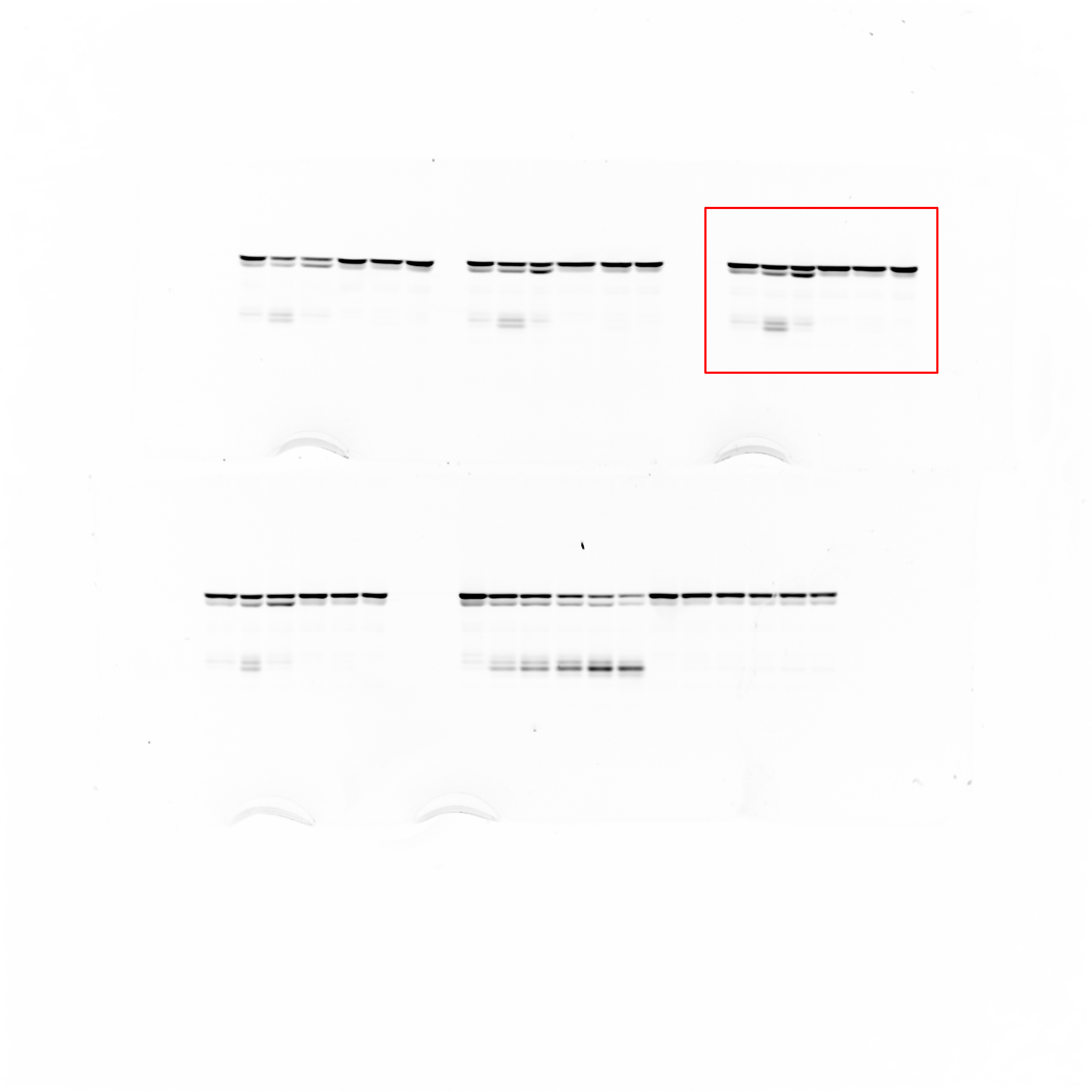

Supplement: Figure 1—source data 1. [file elife-78923-fig1-data1.zip › Figure 1-source data 1/Figure 1c_TMR in-gel fluorescence_annotated.tif]

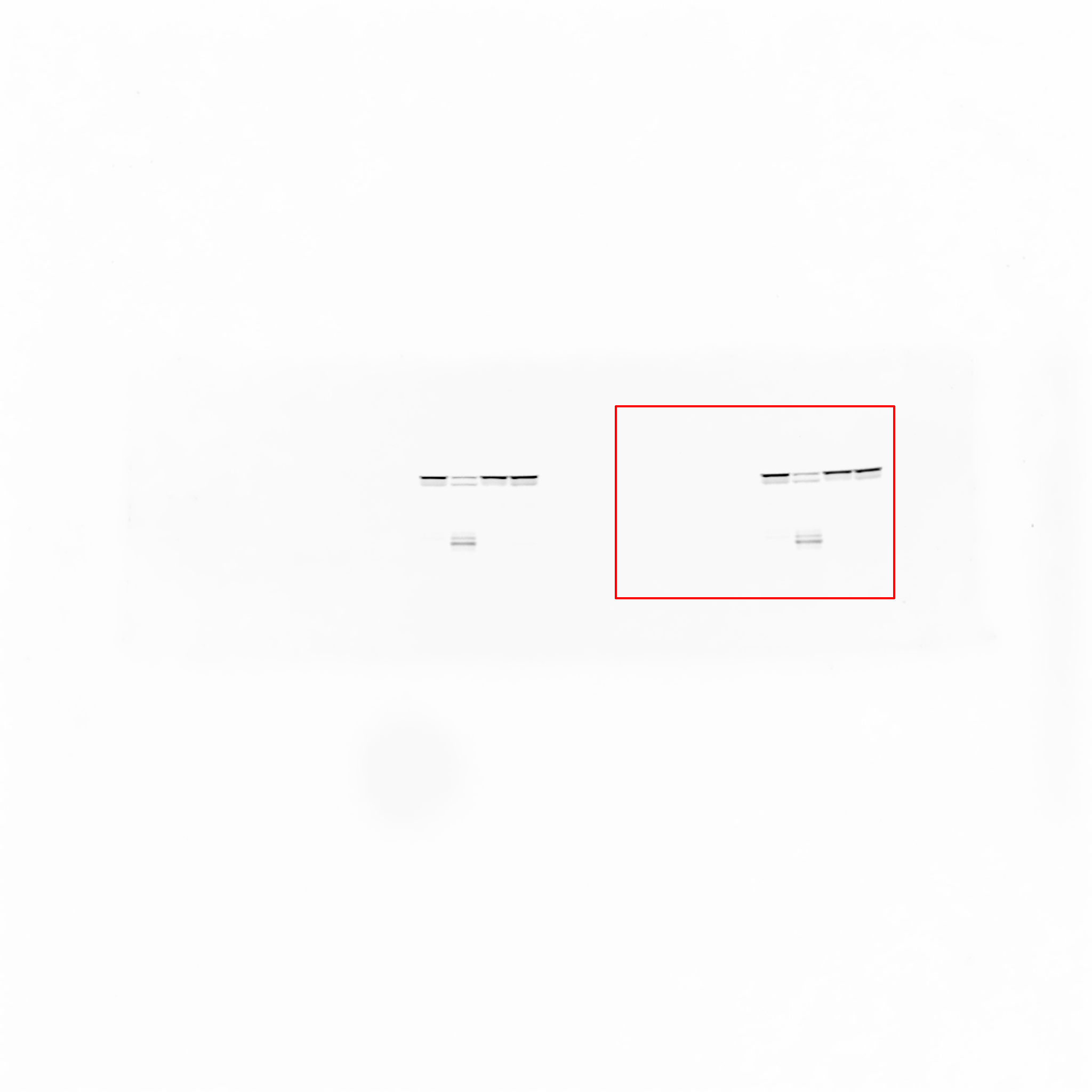

Supplement: Figure 1—source data 1. [file elife-78923-fig1-data1.zip › Figure 1-source data 1/Figure 1b_TMR in-gel fluorescence_annotated.tif]

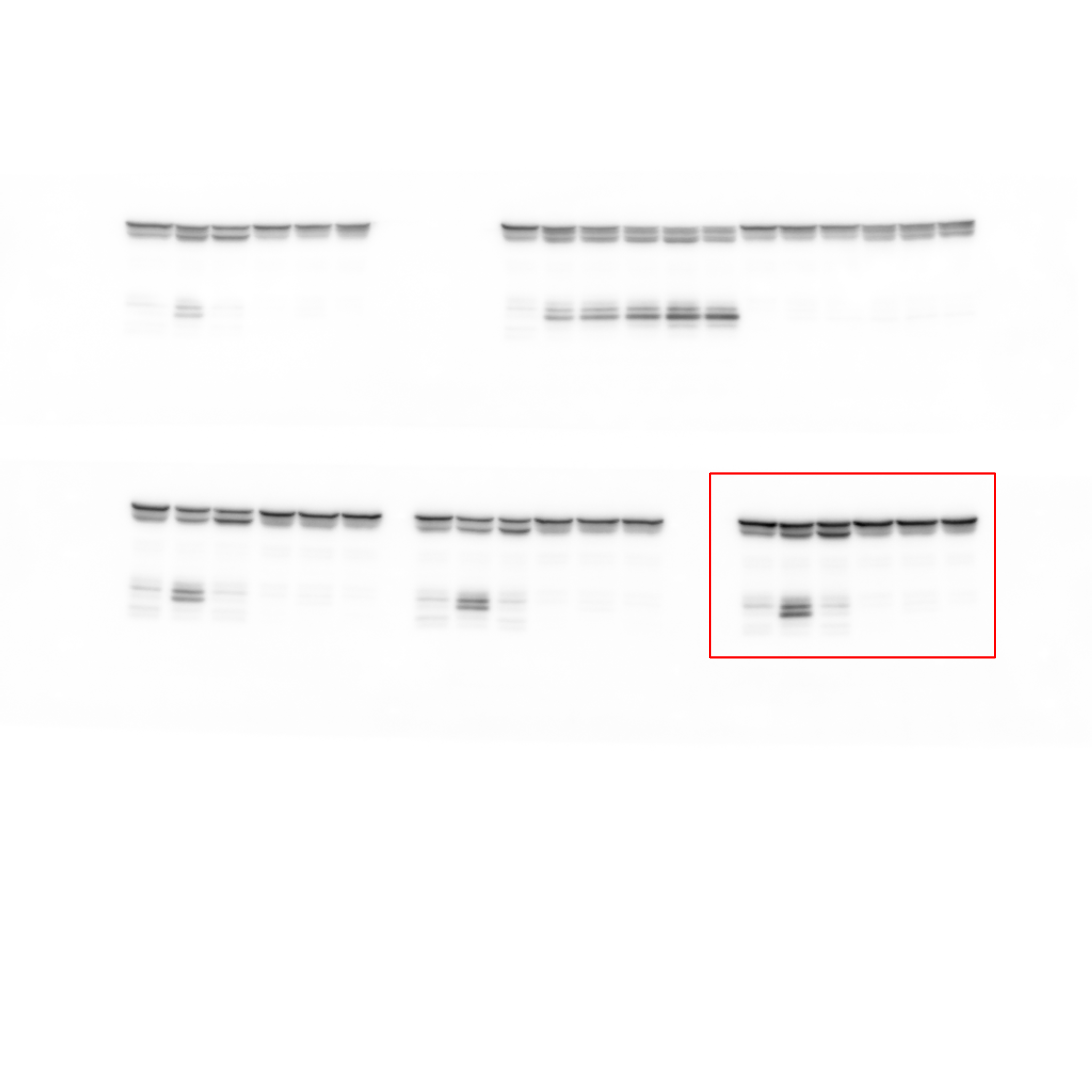

Supplement: Figure 1—source data 1. [file elife-78923-fig1-data1.zip › Figure 1-source data 1/Figure 1c_HaloTag blot_annotated.tif]

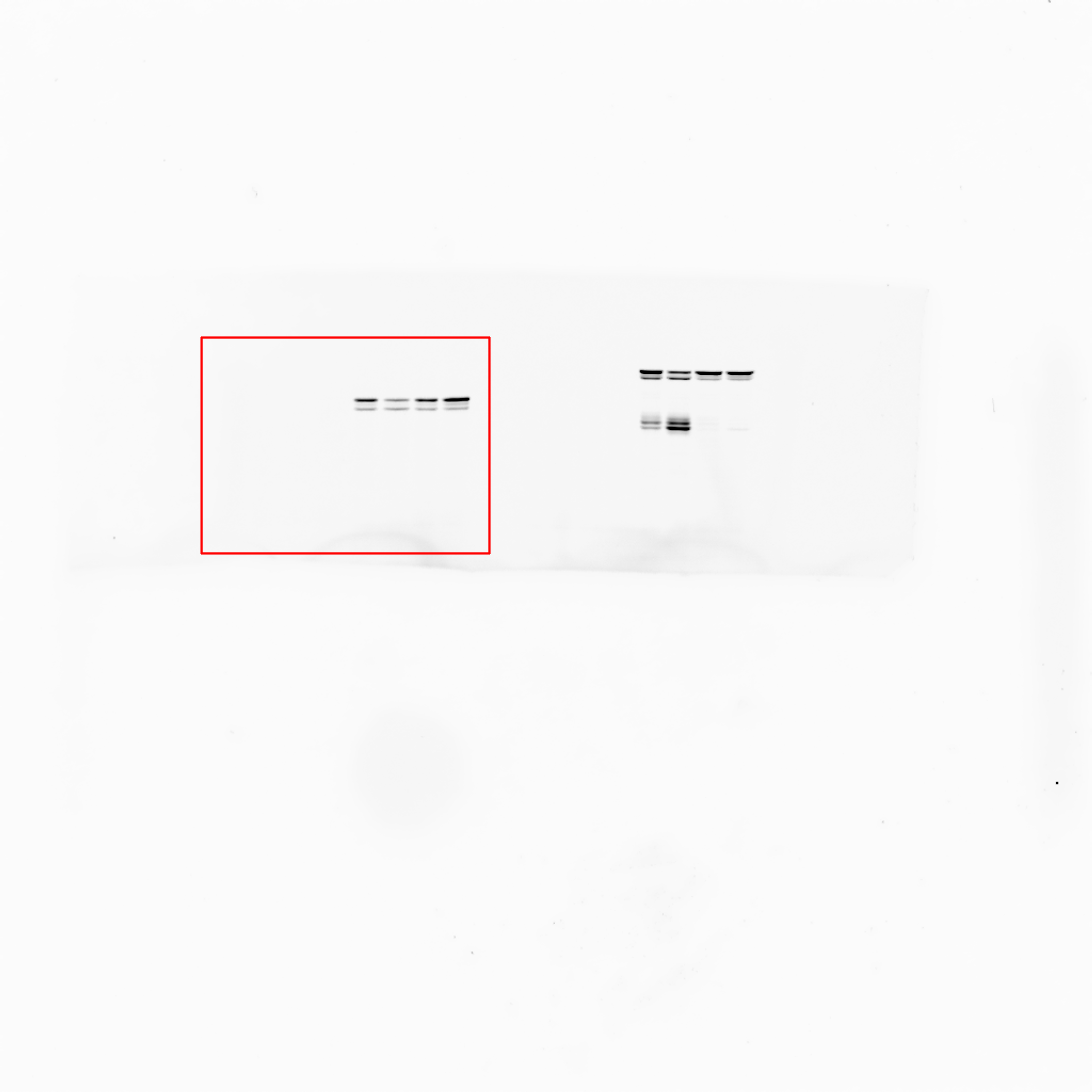

Supplement: Figure 1—source data 1. [file elife-78923-fig1-data1.zip › Figure 1-source data 1/Figure 1d_TMR in-gel fluorescence_annotated.tif]

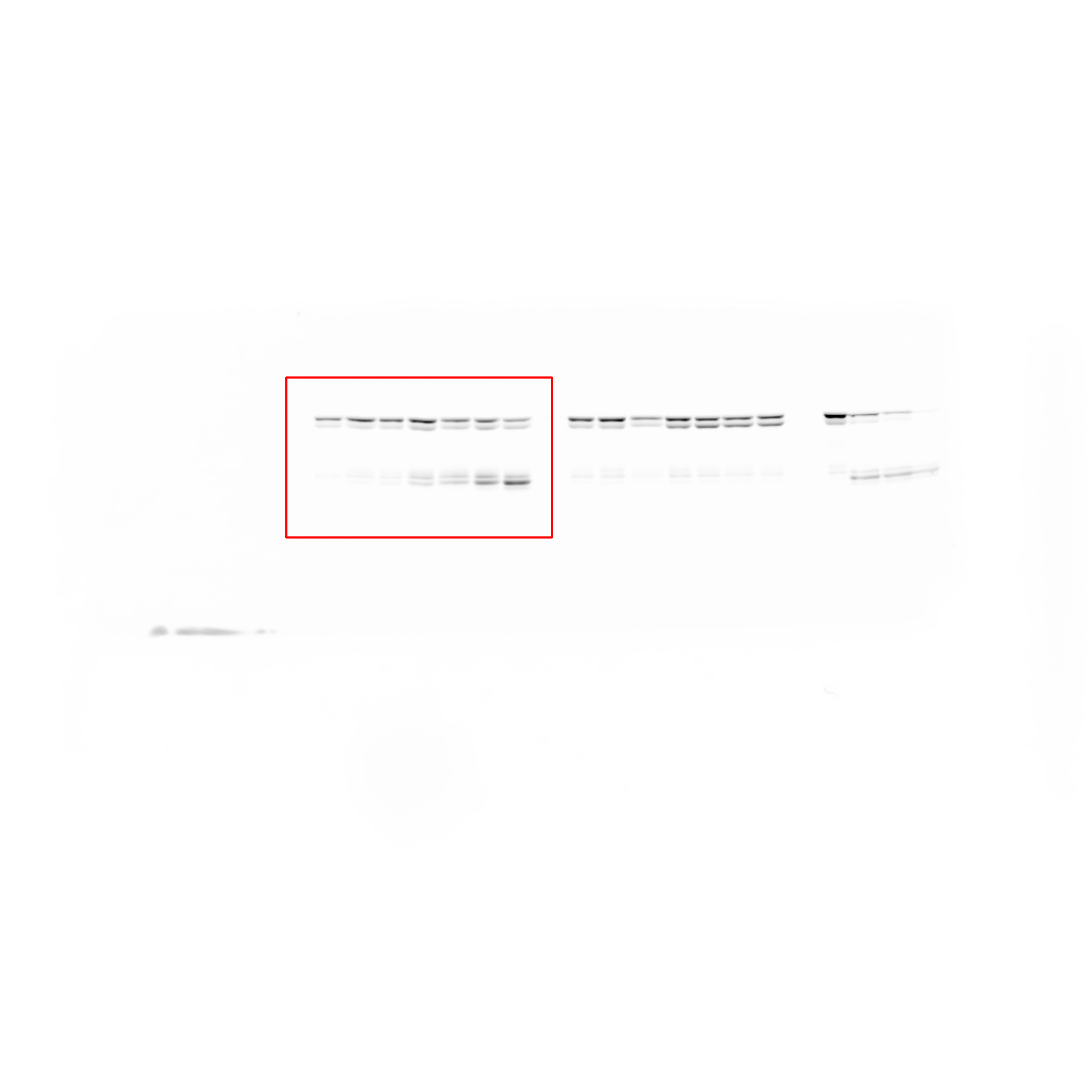

Supplement: Figure 1—source data 1. [file elife-78923-fig1-data1.zip › Figure 1-source data 1/Figure 1f_TMR in-gel fluorescence_annotated.tif]

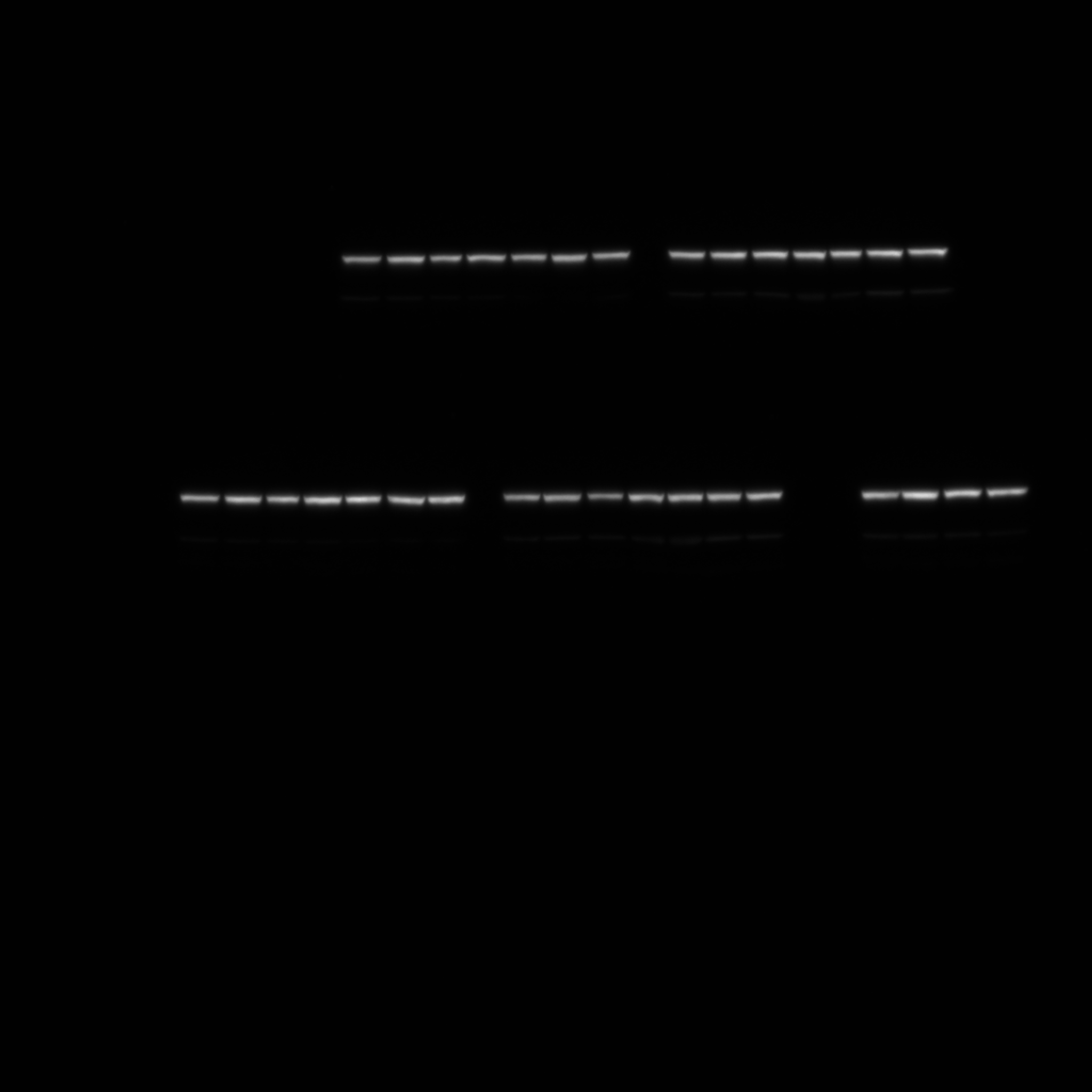

Supplement: Figure 1—source data 1. [file elife-78923-fig1-data1.zip › Figure 1-source data 1/Figure 1f_Hsp90 blot_raw.Tif]

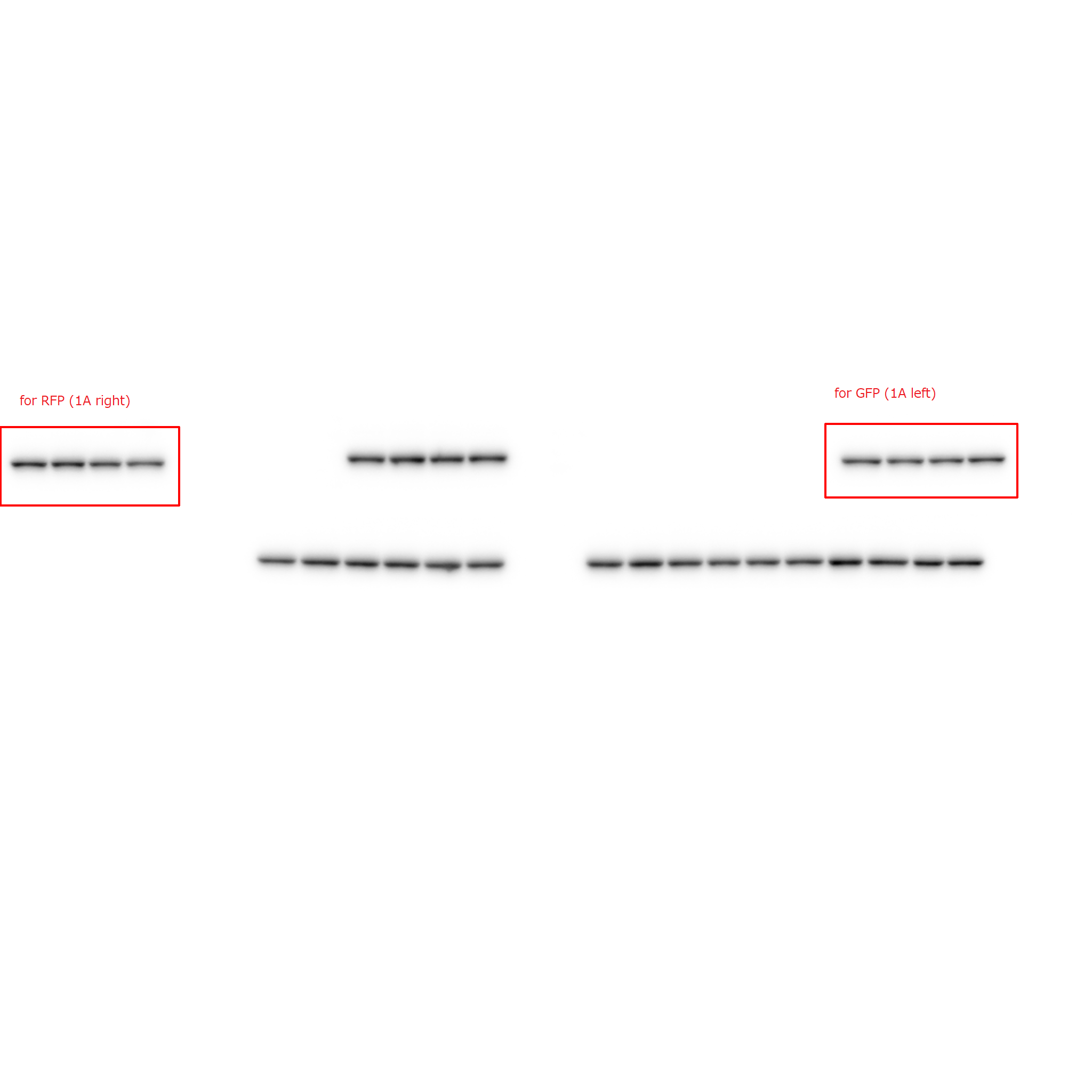

Supplement: Figure 1—source data 1. [file elife-78923-fig1-data1.zip › Figure 1-source data 1/Figure 1a_Hsp90 blot_annotated.tif]

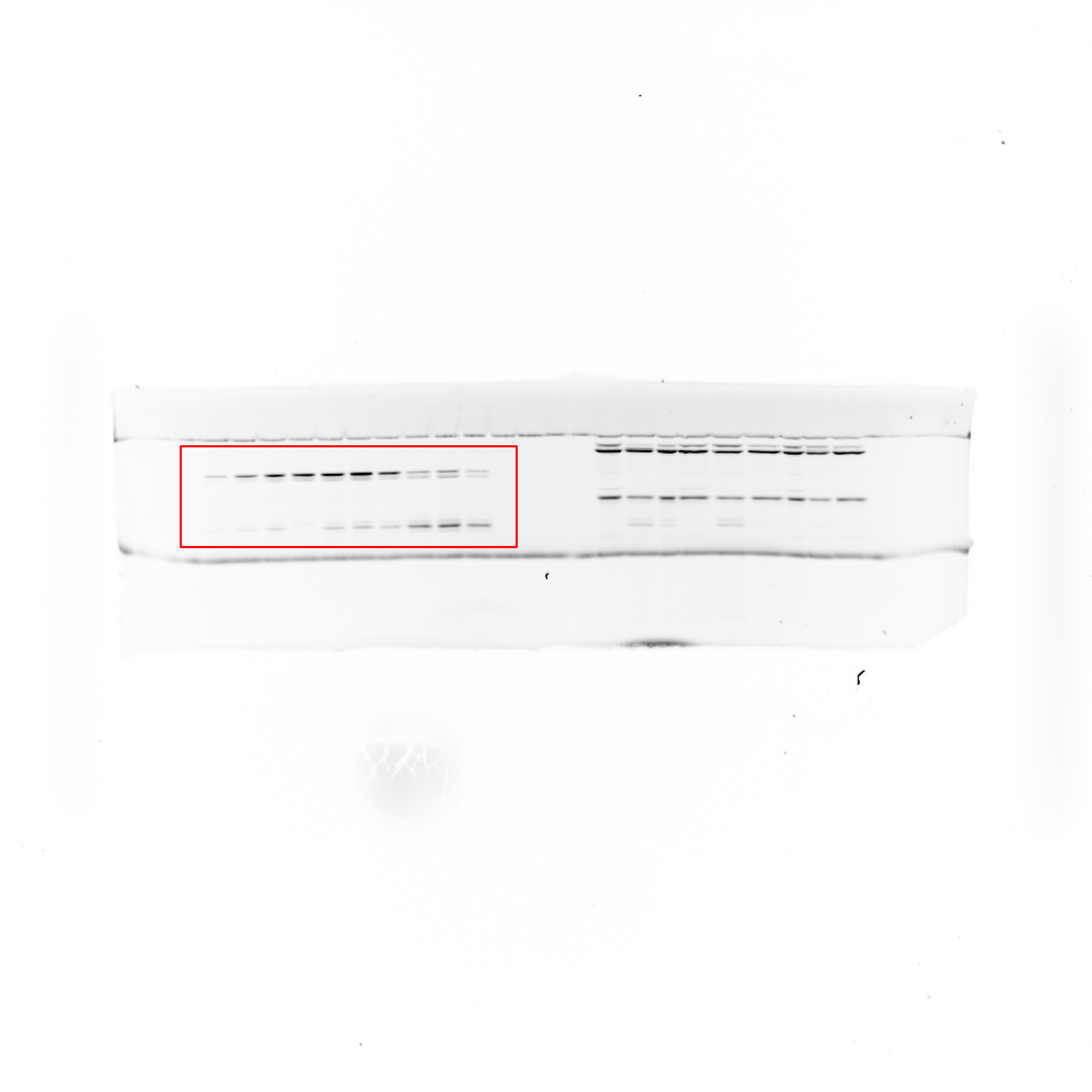

Supplement: Figure 1—source data 1. [file elife-78923-fig1-data1.zip › Figure 1-source data 1/Figure 1-S1_TMR in-gel fluorescence_annotated.tif]

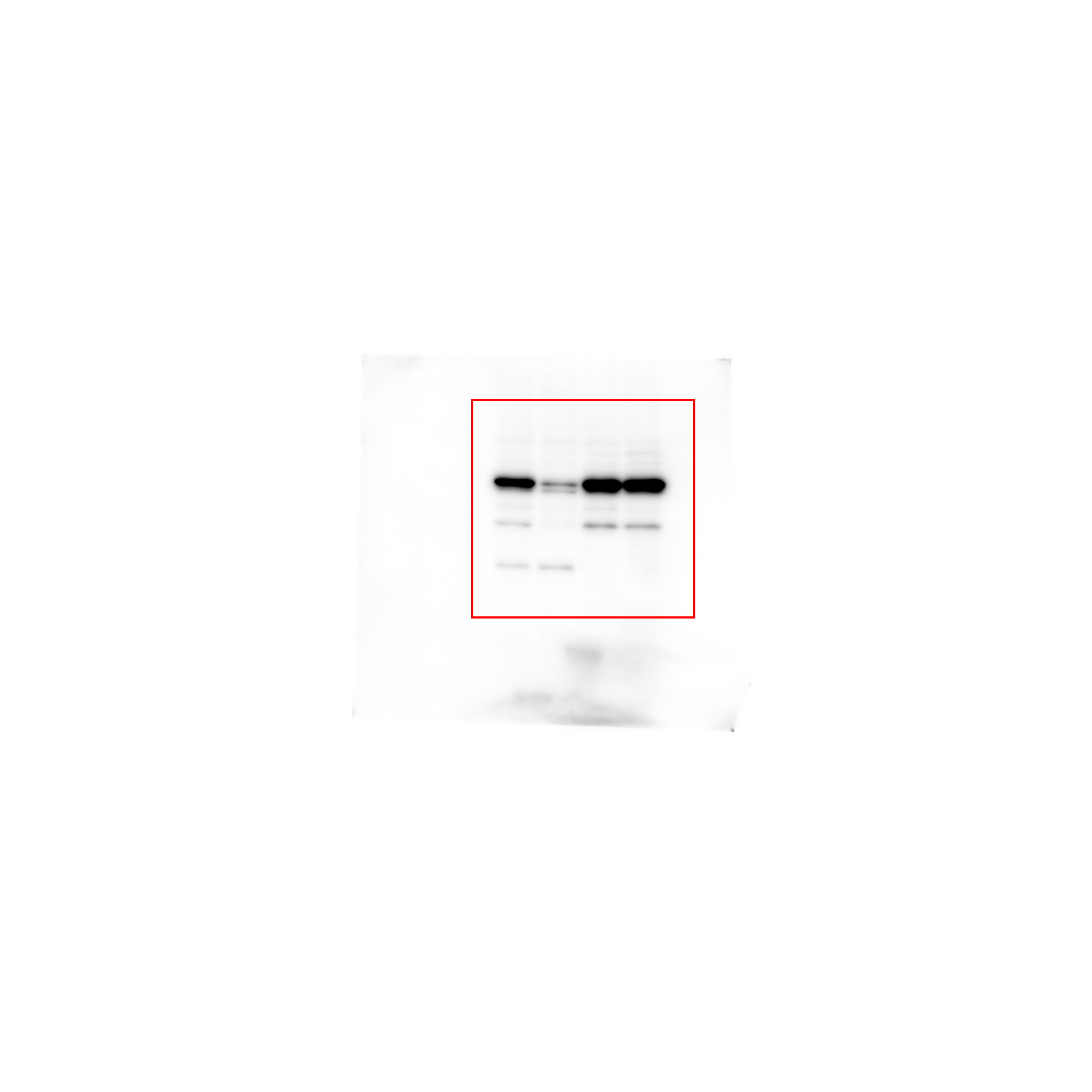

Supplement: Figure 1—source data 1. [file elife-78923-fig1-data1.zip › Figure 1-source data 1/Figure 1a_GFP blot_annotated.tif]

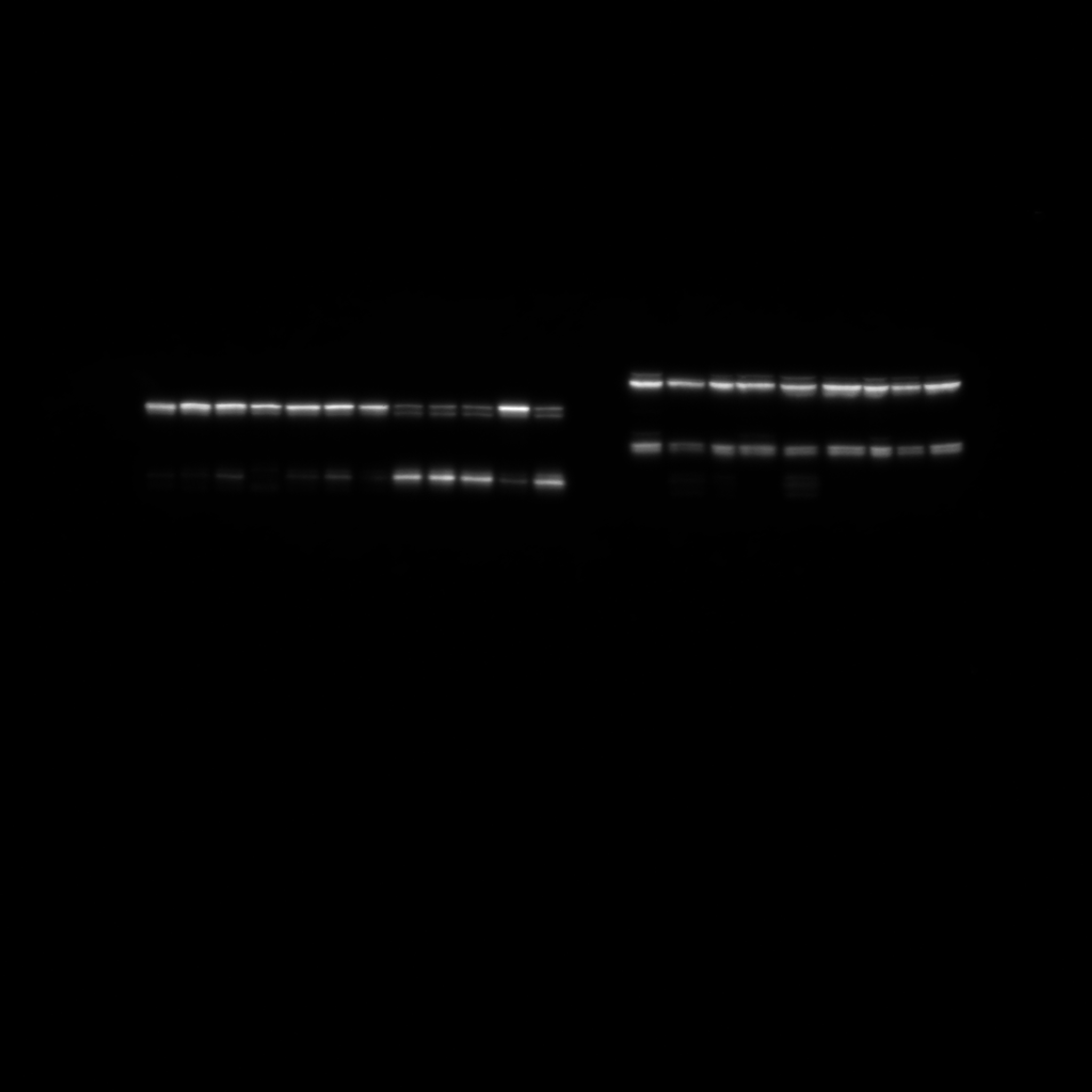

Supplement: Figure 1—source data 1. [file elife-78923-fig1-data1.zip › Figure 1-source data 1/Figure 1-S1_HaloTag blot_raw.Tif]

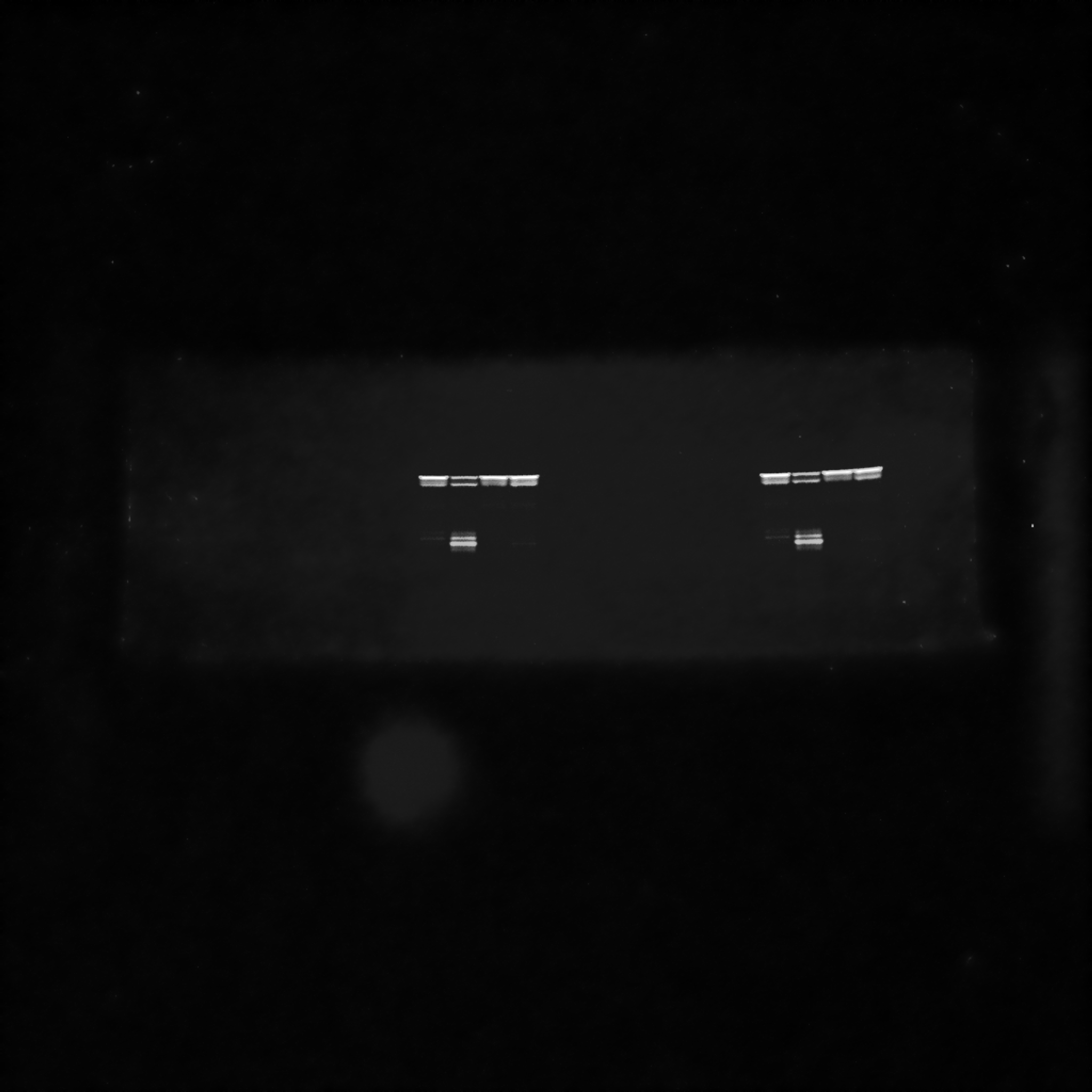

Supplement: Figure 1—source data 1. [file elife-78923-fig1-data1.zip › Figure 1-source data 1/Figure 1b_TMR in-gel fluorescence_raw.TIF]

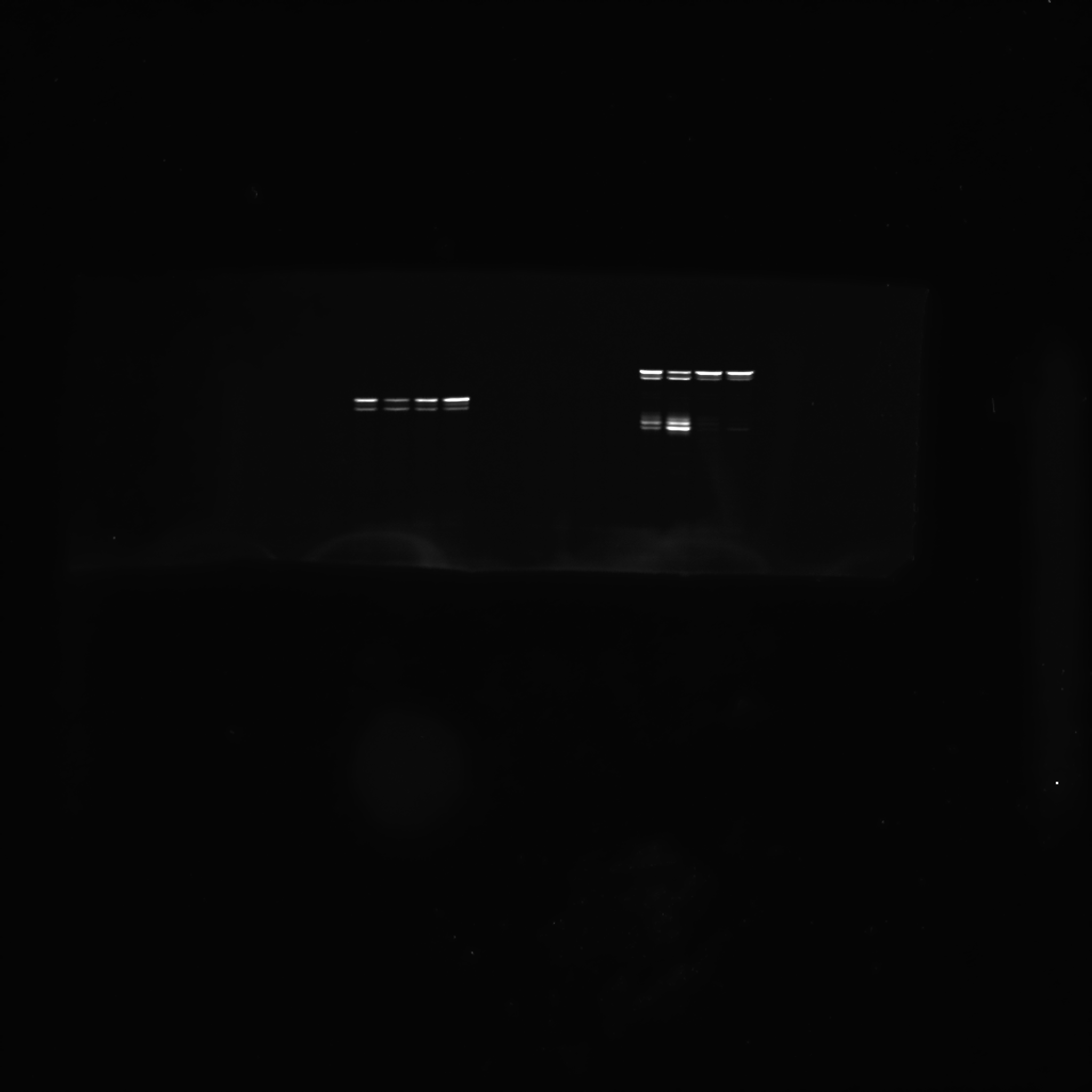

Supplement: Figure 1—source data 1. [file elife-78923-fig1-data1.zip › Figure 1-source data 1/Figure 1d_TMR in-gel fluorescence_raw.TIF]

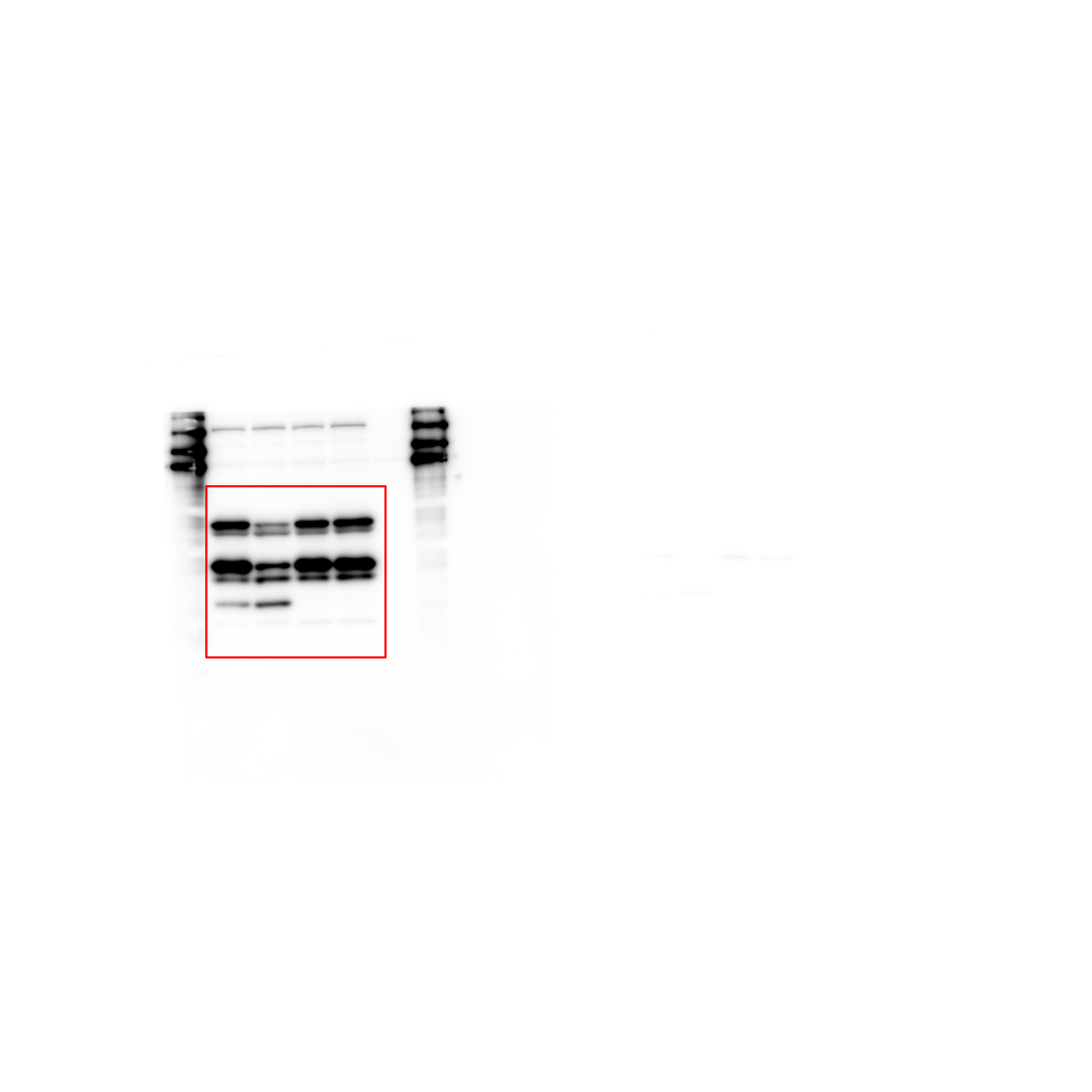

Supplement: Figure 1—source data 1. [file elife-78923-fig1-data1.zip › Figure 1-source data 1/Figure 1a_RFP blot_annotated.tif]

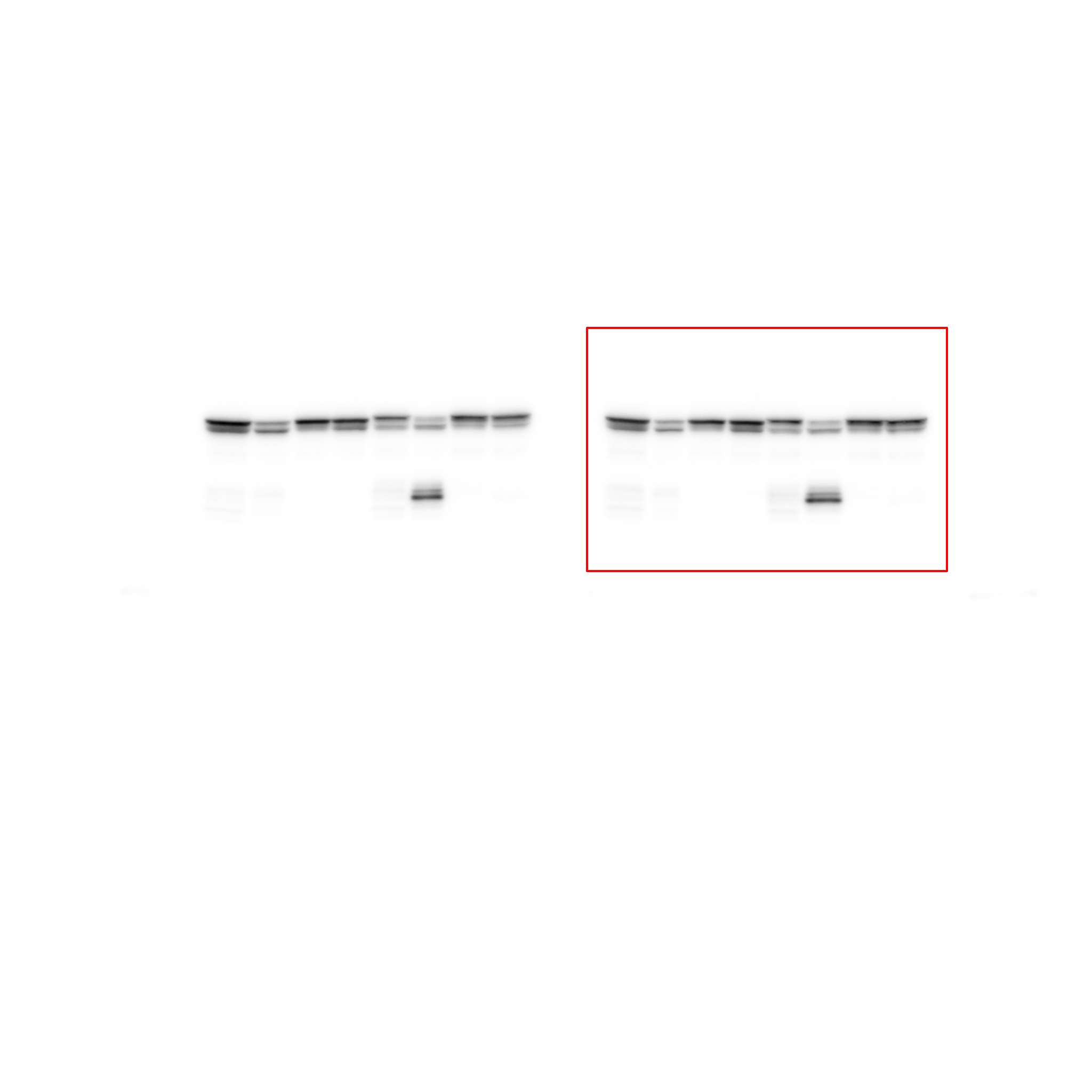

Supplement: Figure 1—source data 1. [file elife-78923-fig1-data1.zip › Figure 1-source data 1/Figure 1b_HaloTag blot_annotated.tif]

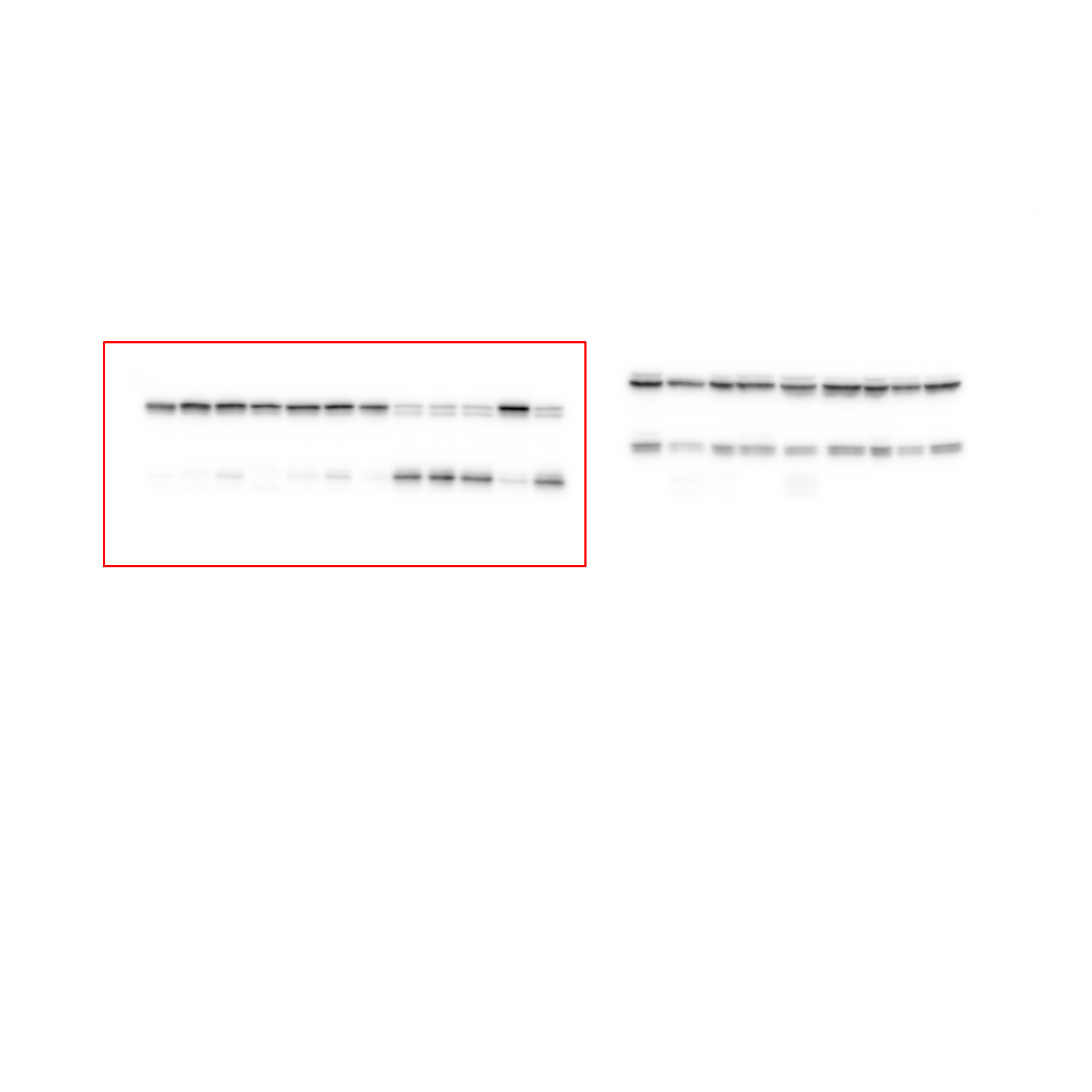

Supplement: Figure 1—source data 1. [file elife-78923-fig1-data1.zip › Figure 1-source data 1/Figure 1-S1_HaloTag blot_annotated.tif]

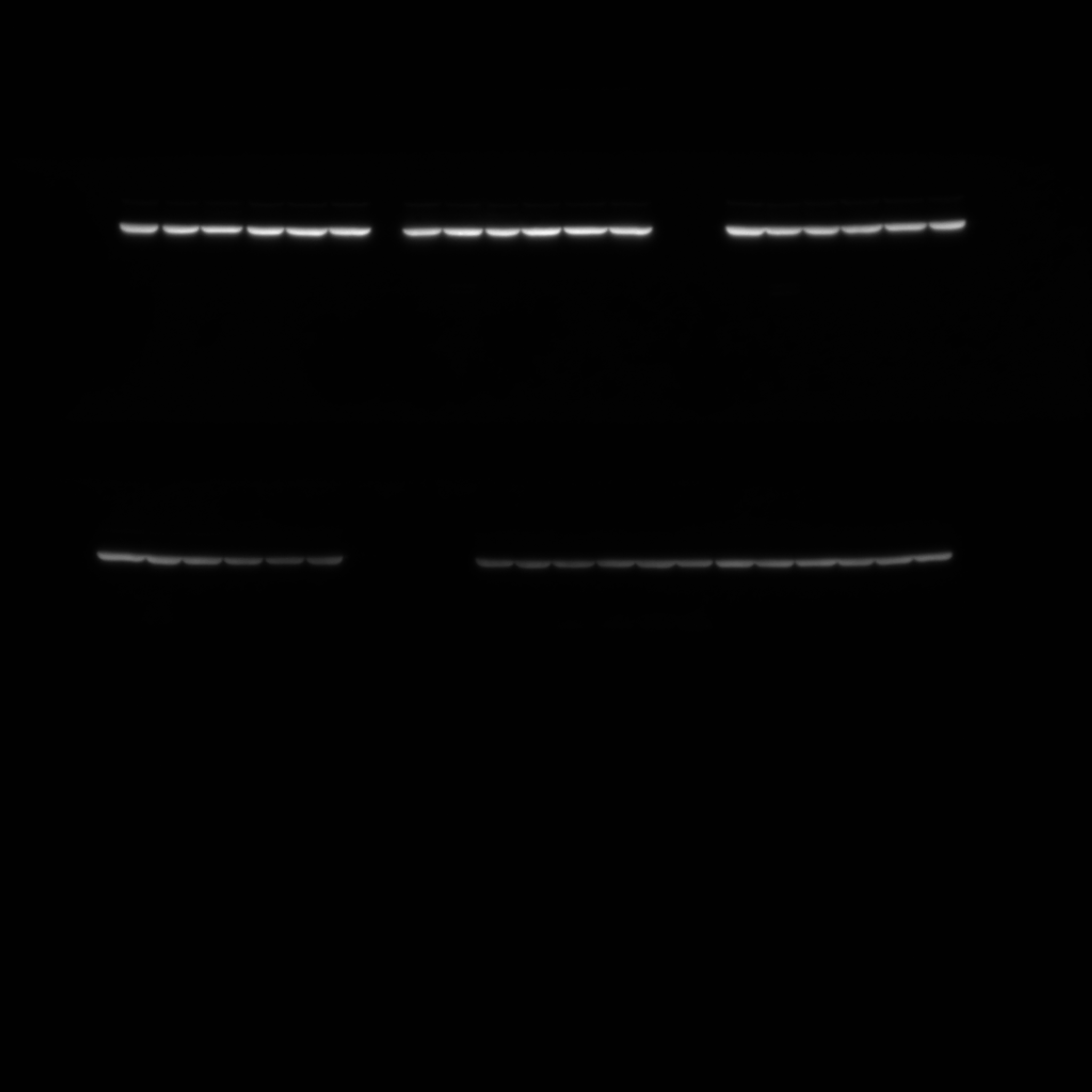

Supplement: Figure 1—source data 1. [file elife-78923-fig1-data1.zip › Figure 1-source data 1/Figure 1c_beta-actin blot_raw.Tif]

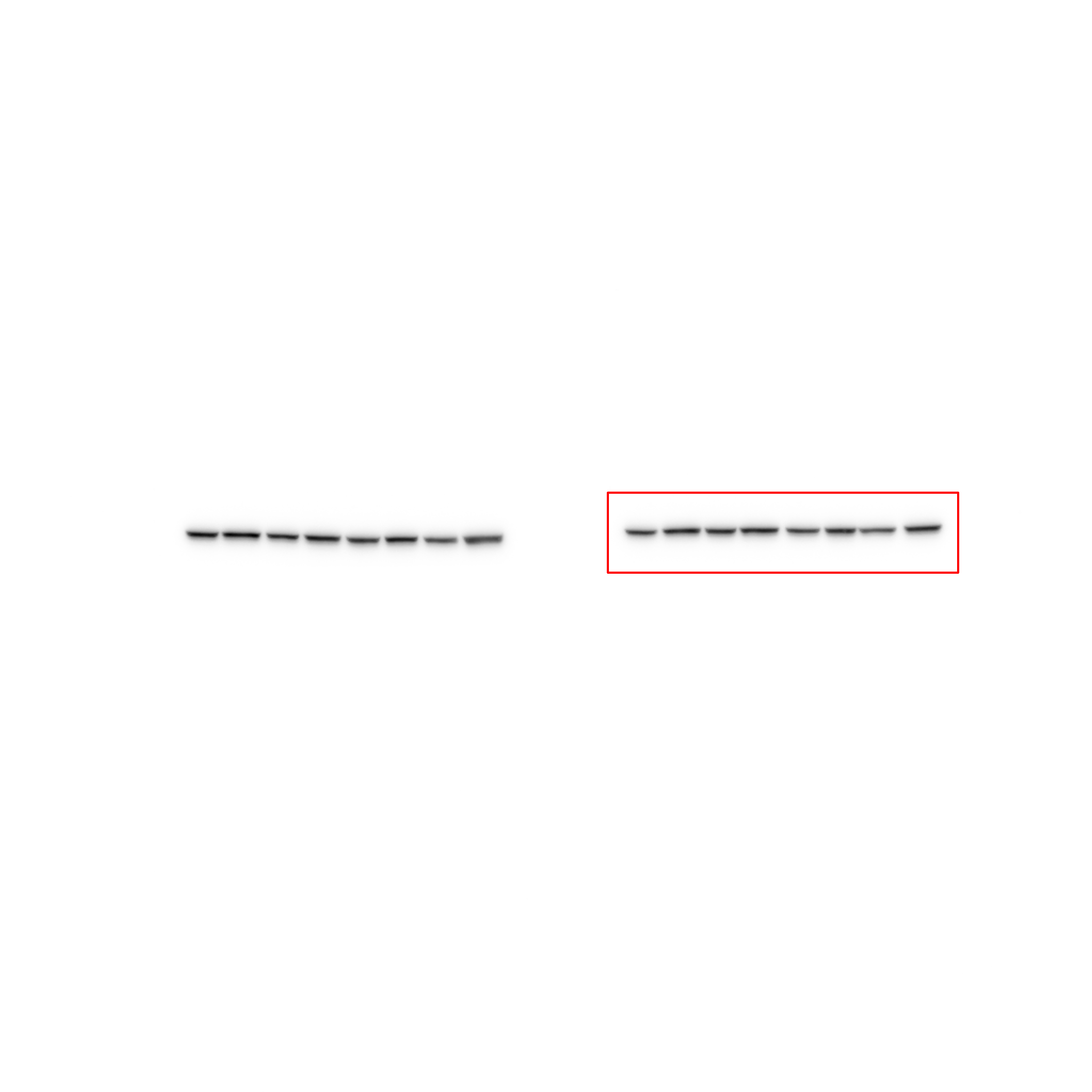

Supplement: Figure 1—source data 1. [file elife-78923-fig1-data1.zip › Figure 1-source data 1/Figure 1d_Hsp90 blot_annotated.tif]

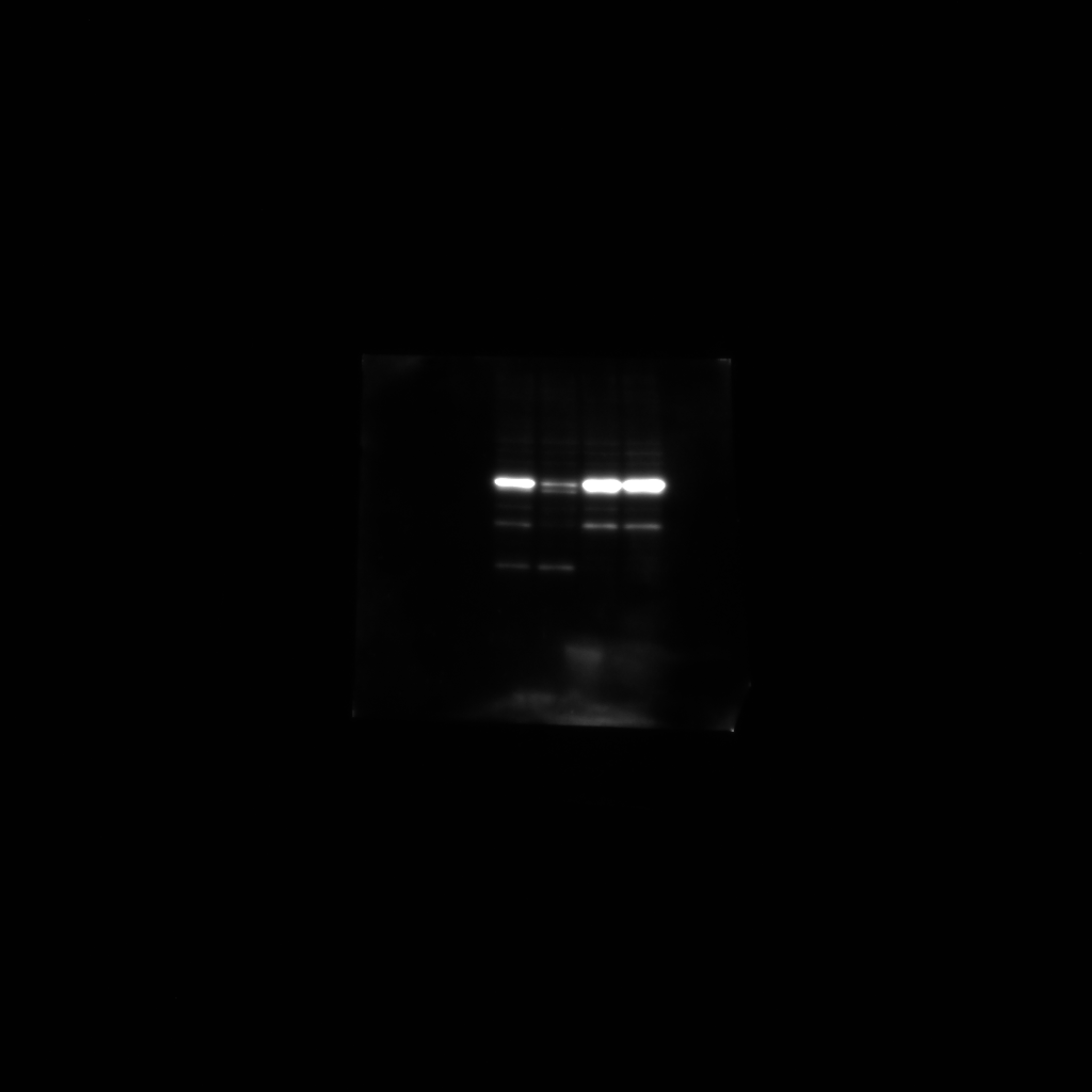

Supplement: Figure 1—source data 1. [file elife-78923-fig1-data1.zip › Figure 1-source data 1/Figure 1a_GFP blot_raw.Tif]

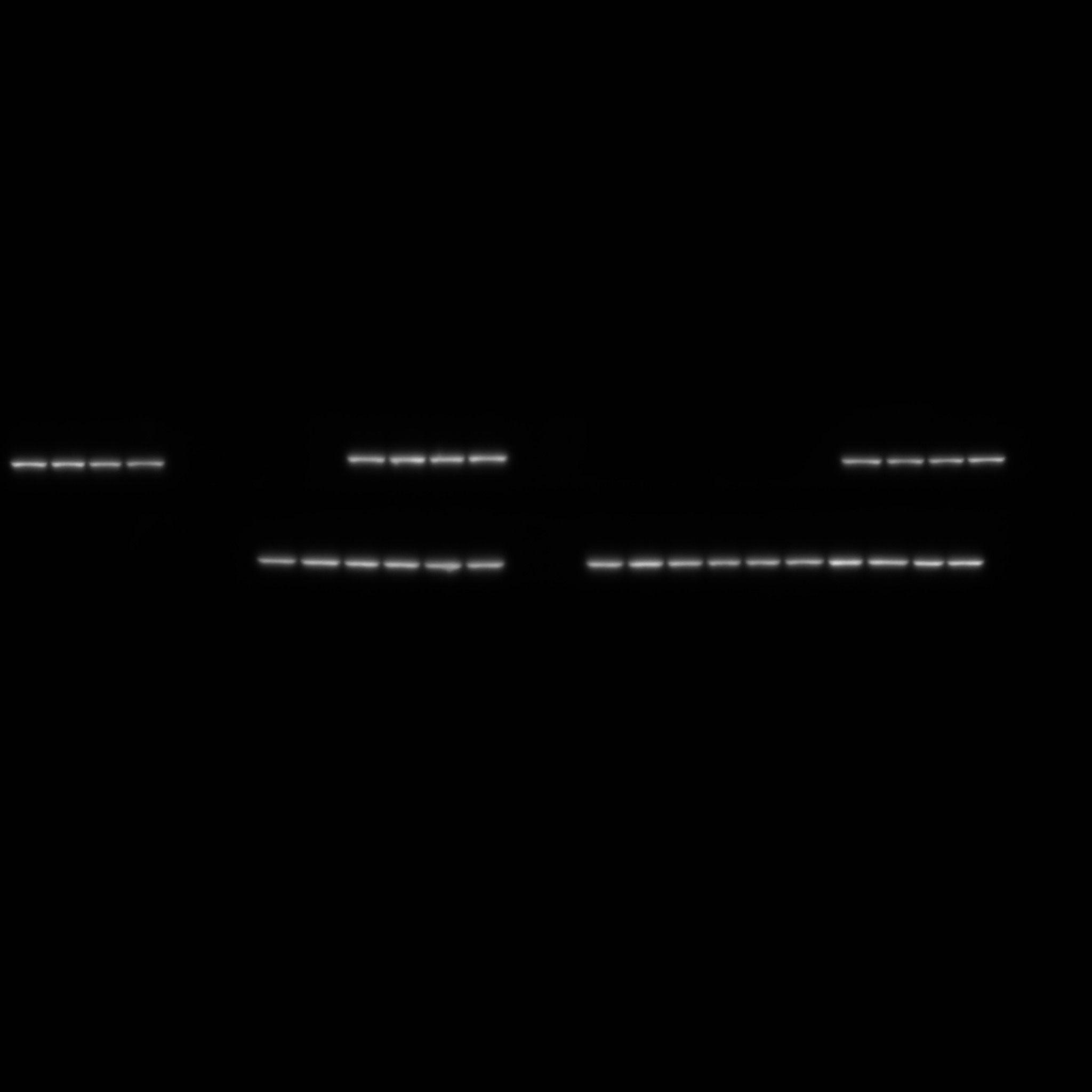

Supplement: Figure 1—source data 1. [file elife-78923-fig1-data1.zip › Figure 1-source data 1/Figure 1a_Hsp90 blot_raw.Tif]

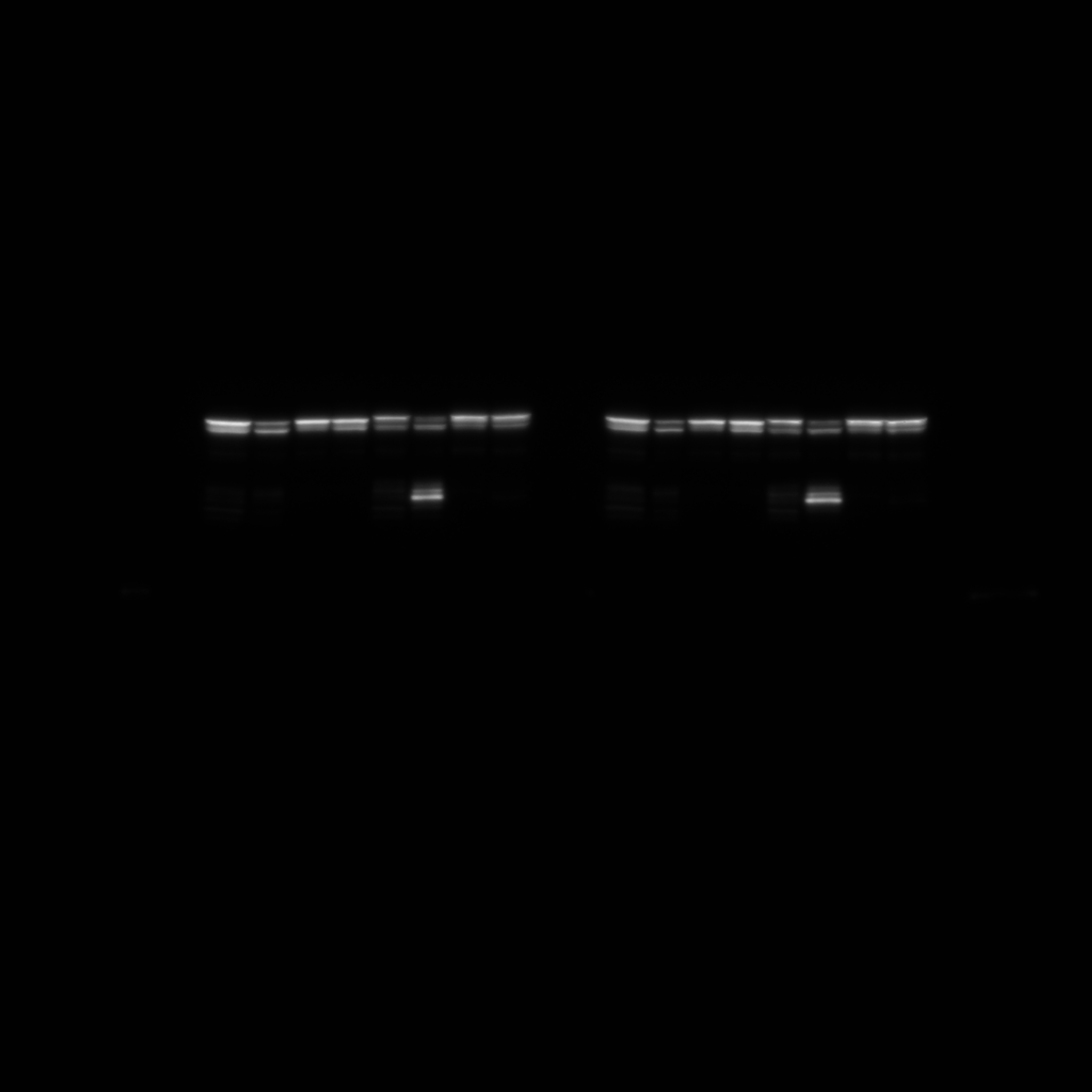

Supplement: Figure 1—source data 1. [file elife-78923-fig1-data1.zip › Figure 1-source data 1/Figure 1b_HaloTag blot_raw.Tif]

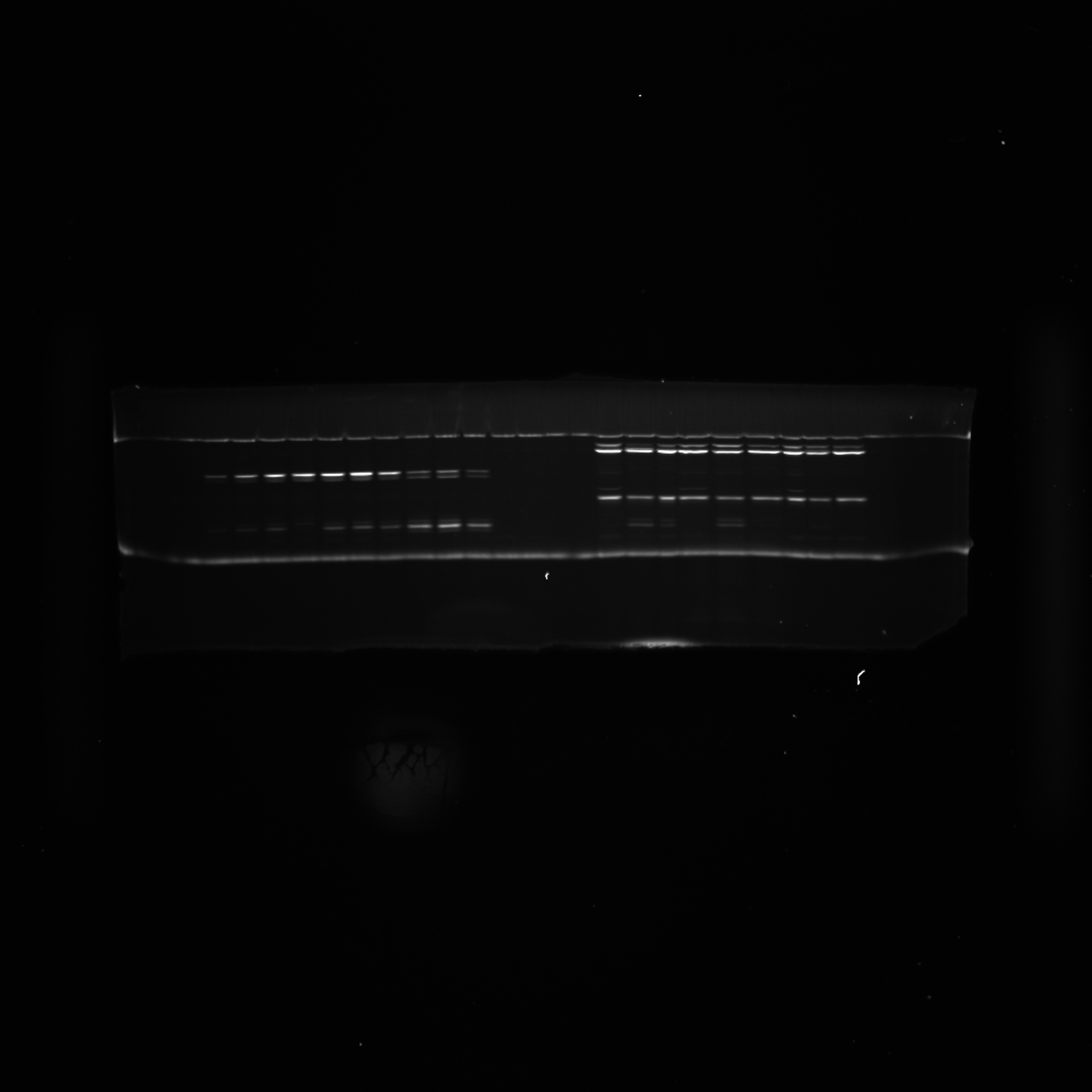

Supplement: Figure 1—source data 1. [file elife-78923-fig1-data1.zip › Figure 1-source data 1/Figure 1-S1_TMR in-gel fluorescence_raw.TIF]

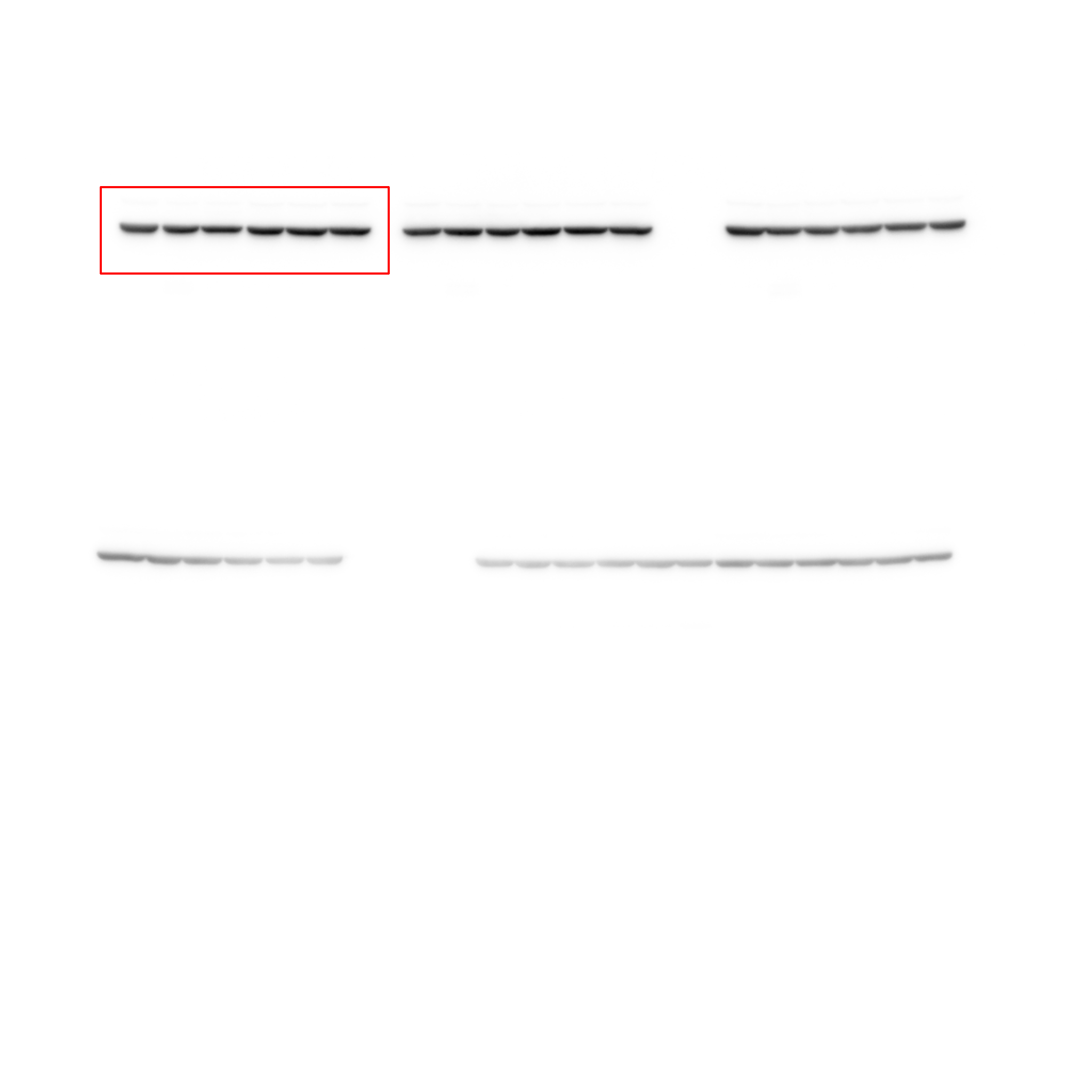

Supplement: Figure 1—source data 1. [file elife-78923-fig1-data1.zip › Figure 1-source data 1/Figure 1c_beta-actin blot_annotated.tif]

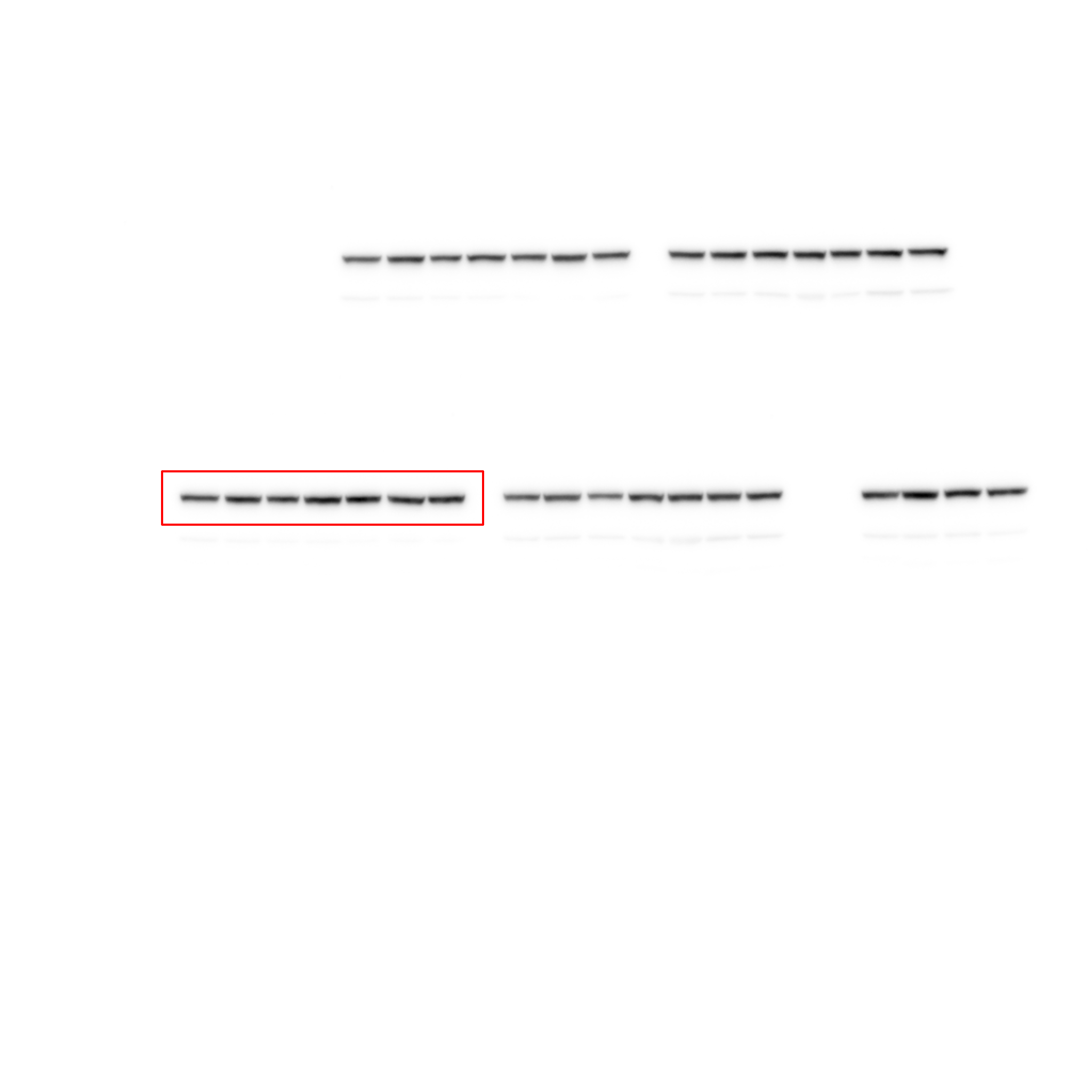

Supplement: Figure 1—source data 1. [file elife-78923-fig1-data1.zip › Figure 1-source data 1/Figure 1f_Hsp90 blot_annotated.tif]

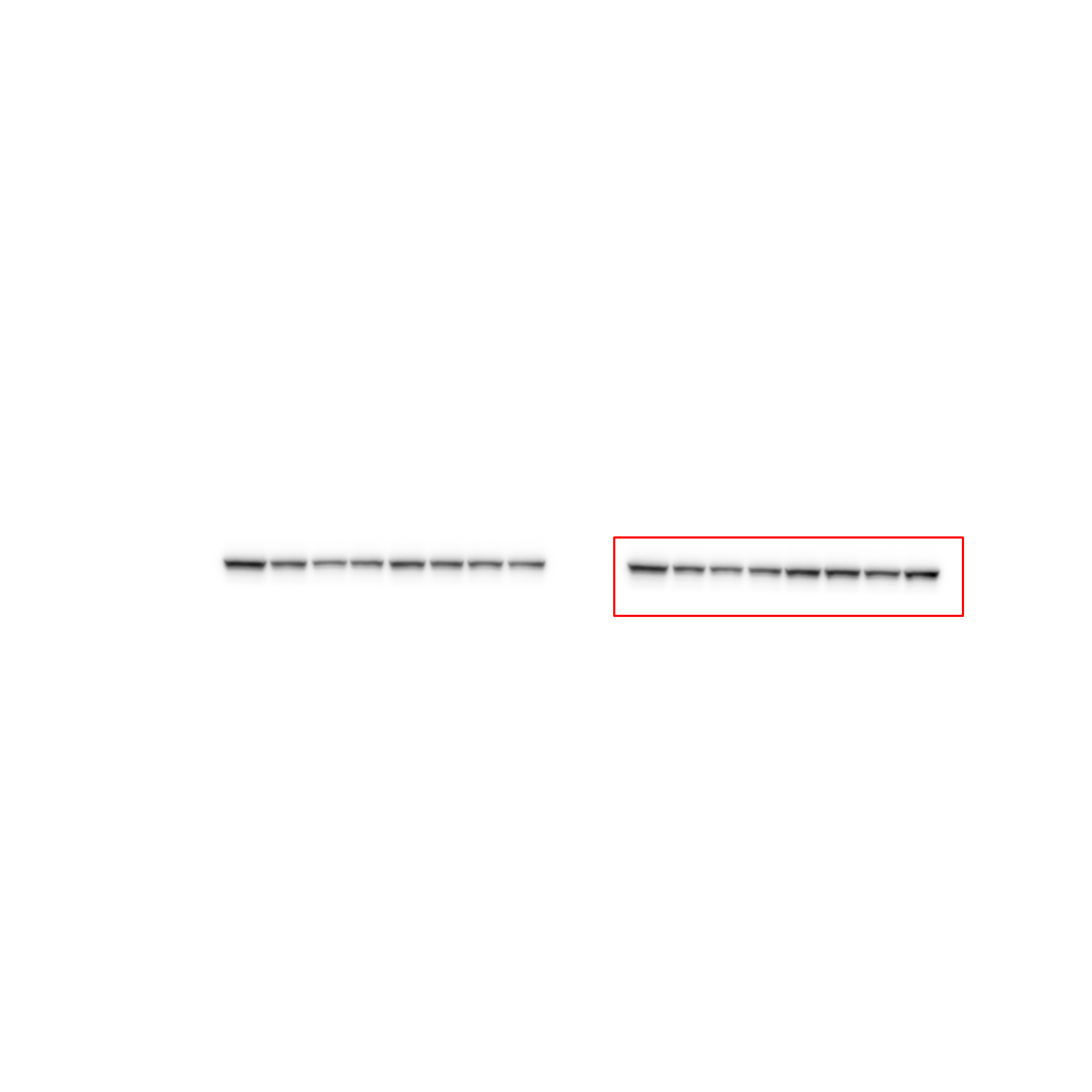

Supplement: Figure 1—source data 1. [file elife-78923-fig1-data1.zip › Figure 1-source data 1/Figure 1b_Hsp90 blot_annotated.tif]

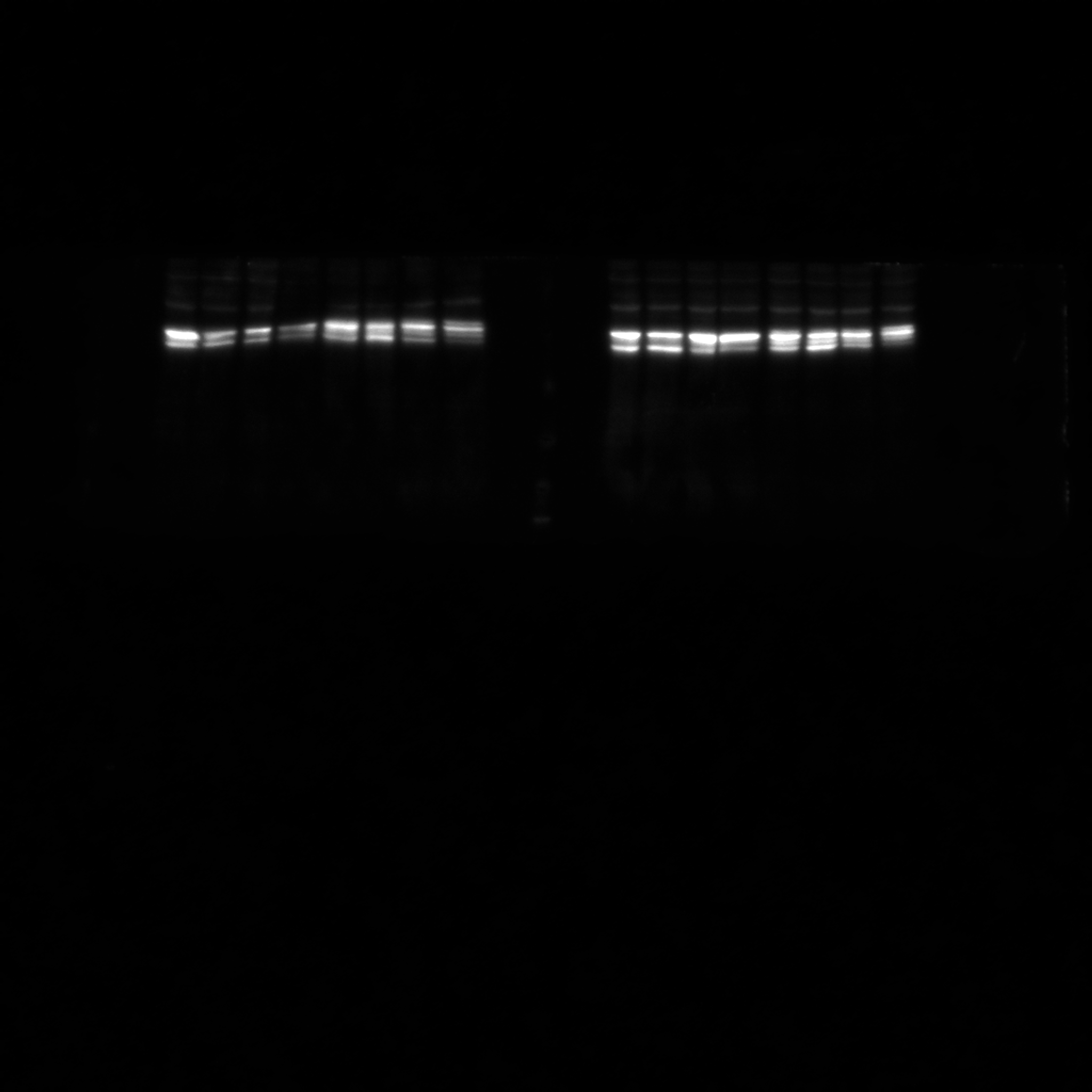

Supplement: Figure 1—source data 1. [file elife-78923-fig1-data1.zip › Figure 1-source data 1/Figure 1d_SNAP blot_raw.Tif]

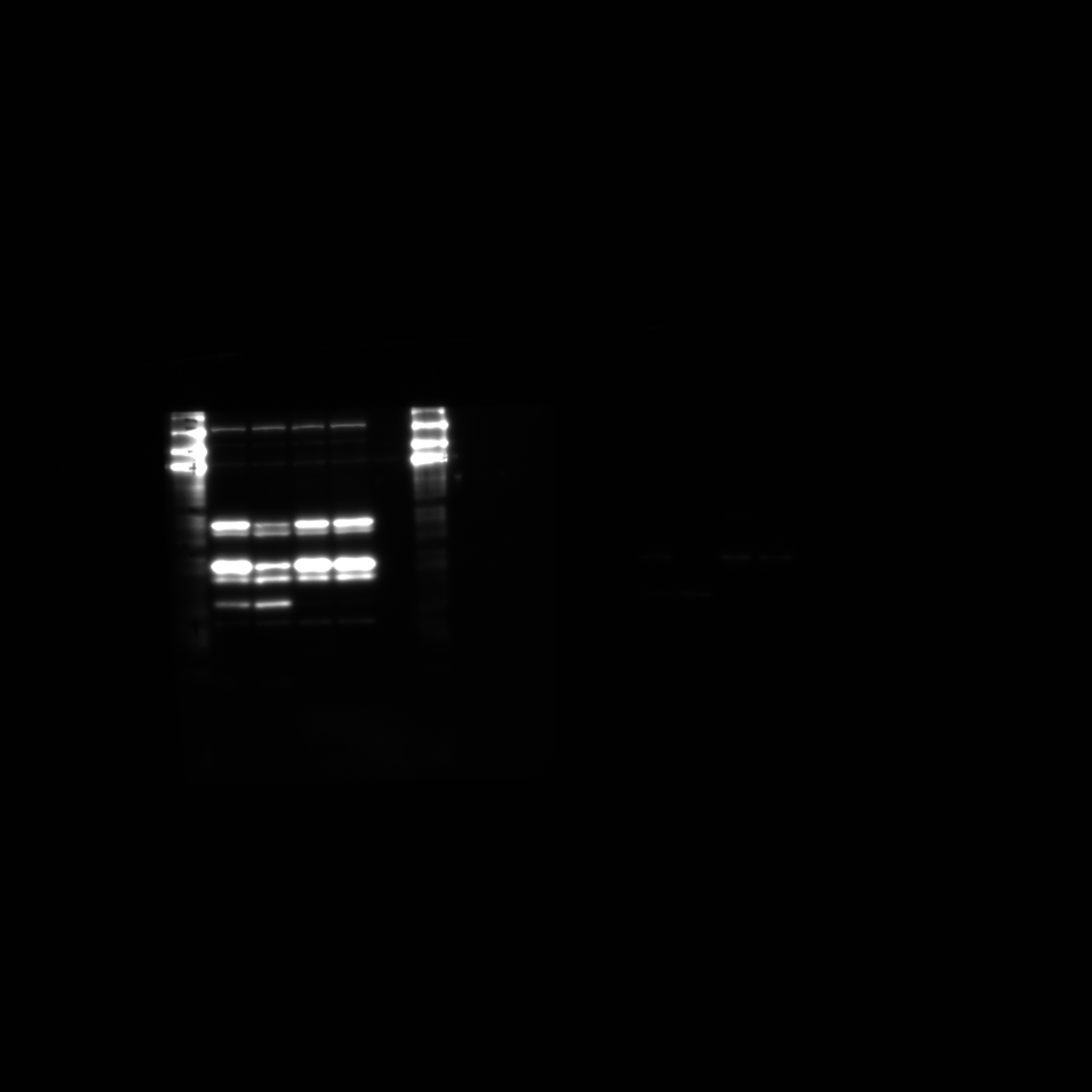

Supplement: Figure 1—source data 1. [file elife-78923-fig1-data1.zip › Figure 1-source data 1/Figure 1a_RFP blot_raw.Tif]

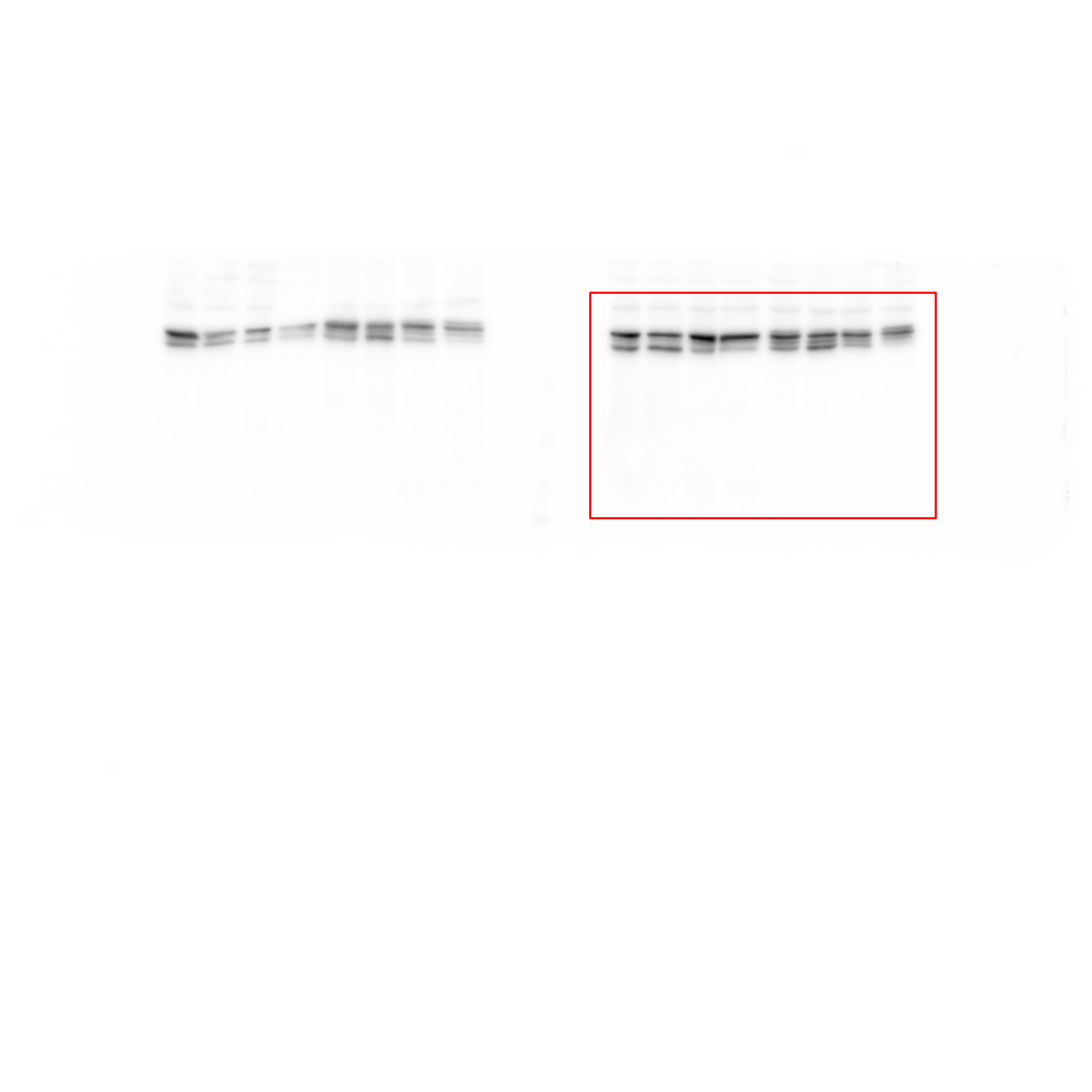

Supplement: Figure 1—source data 1. [file elife-78923-fig1-data1.zip › Figure 1-source data 1/Figure 1d_SNAP blot_annotated.tif]

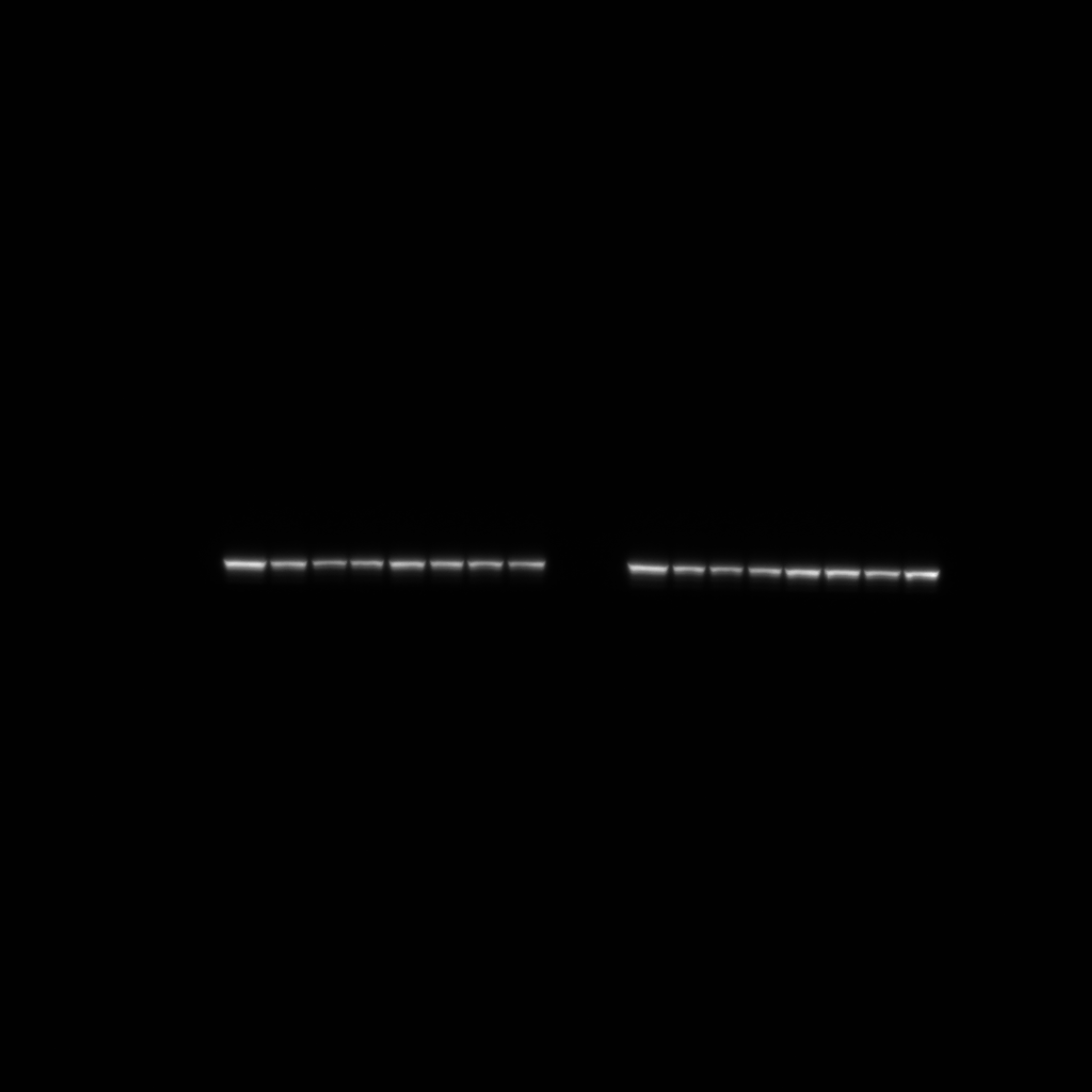

Supplement: Figure 1—source data 1. [file elife-78923-fig1-data1.zip › Figure 1-source data 1/Figure 1b_Hsp90 blot_raw.Tif]

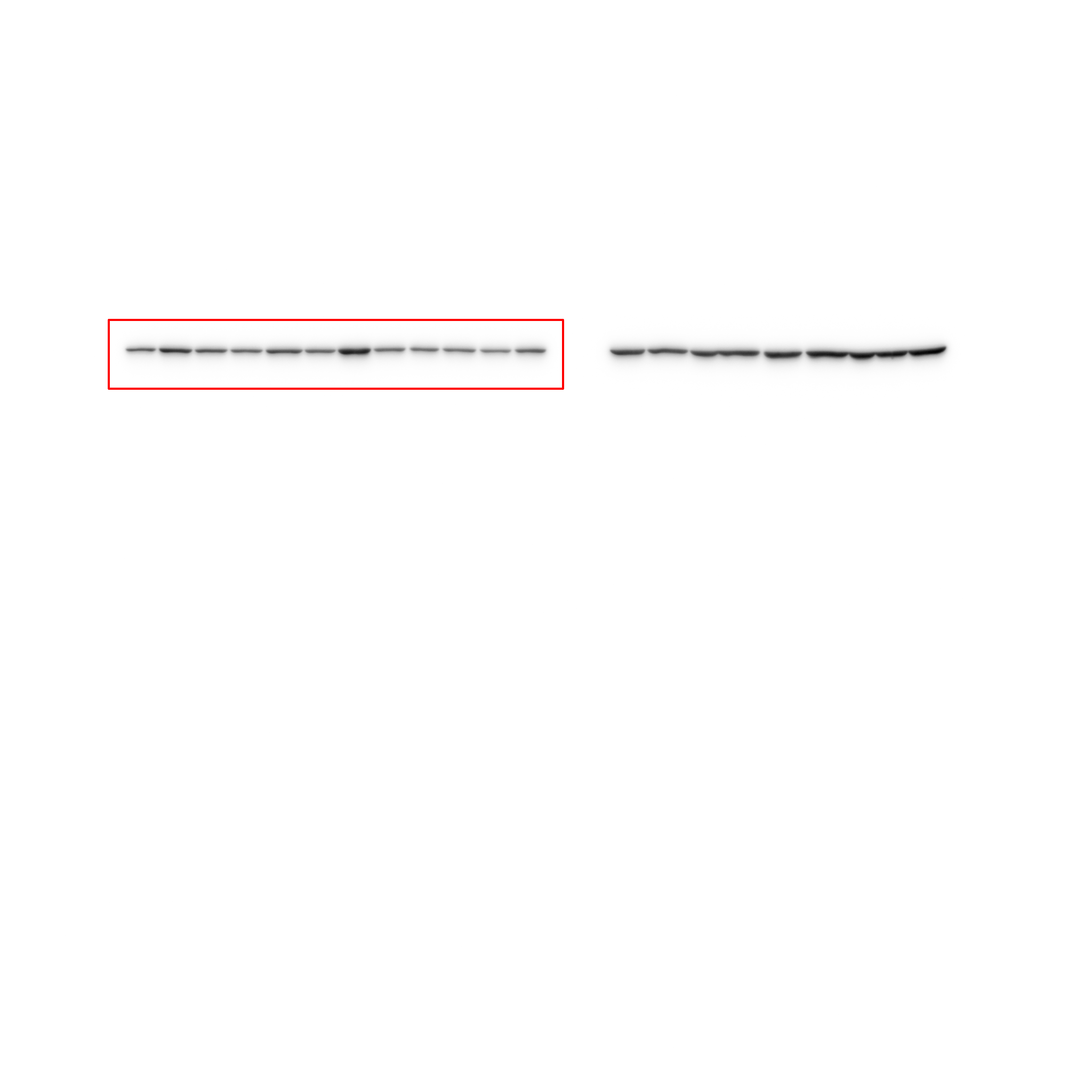

Supplement: Figure 1—source data 1. [file elife-78923-fig1-data1.zip › Figure 1-source data 1/Figure 1-S1_beta-actin blot_annotated.tif]

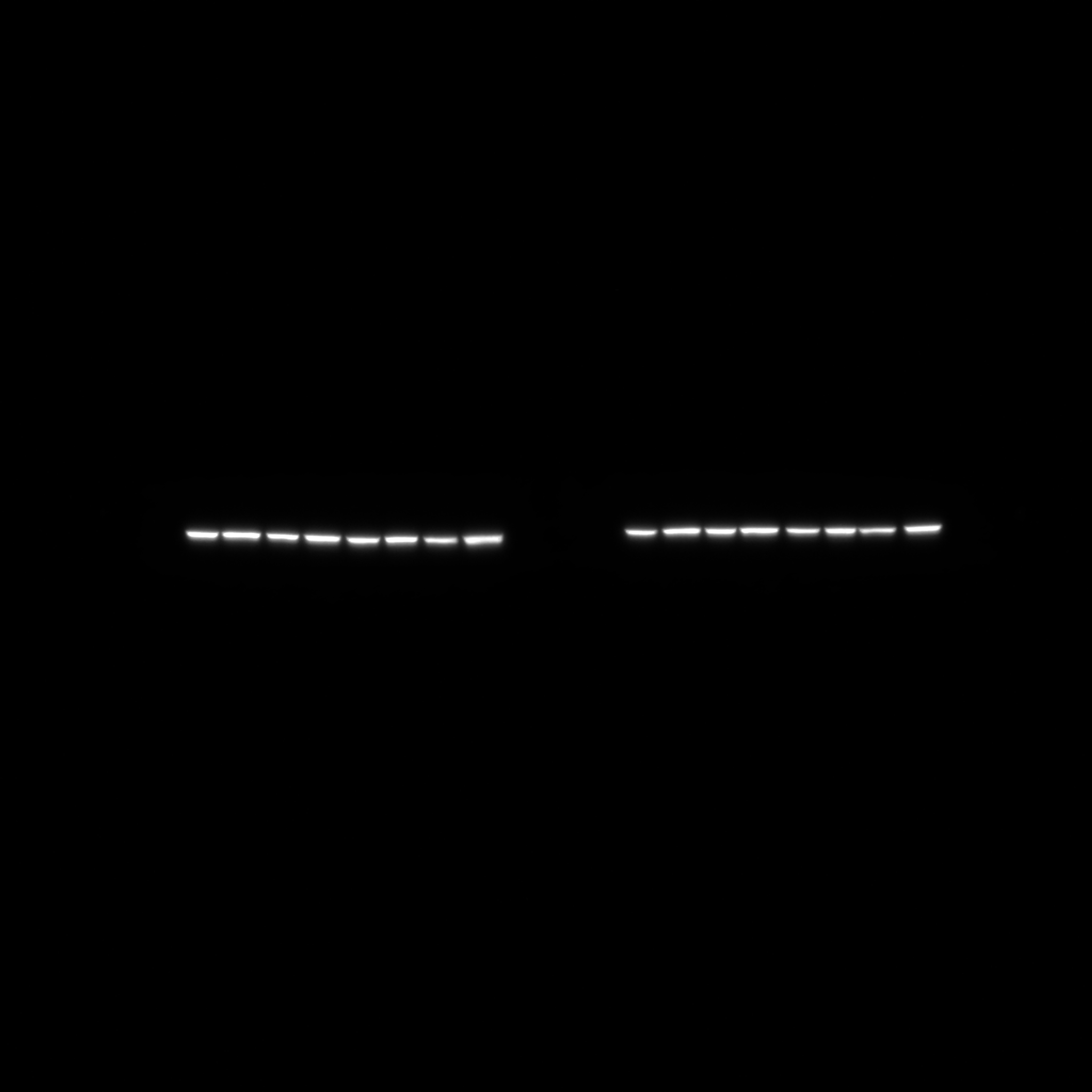

Supplement: Figure 1—source data 1. [file elife-78923-fig1-data1.zip › Figure 1-source data 1/Figure 1d_Hsp90 blot_raw.Tif]

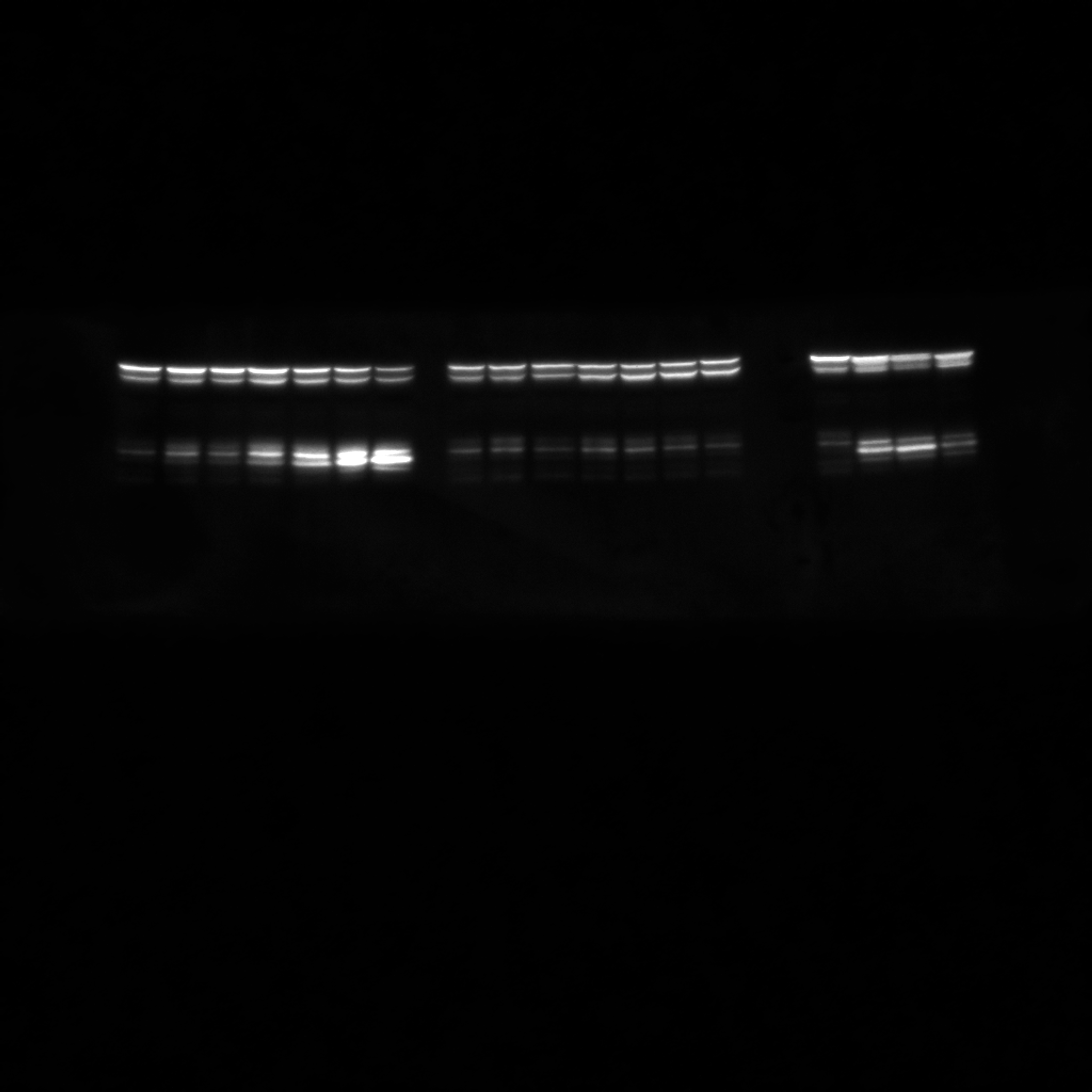

Supplement: Figure 1—source data 1. [file elife-78923-fig1-data1.zip › Figure 1-source data 1/Figure 1f_HaloTag blot_raw.TIF]

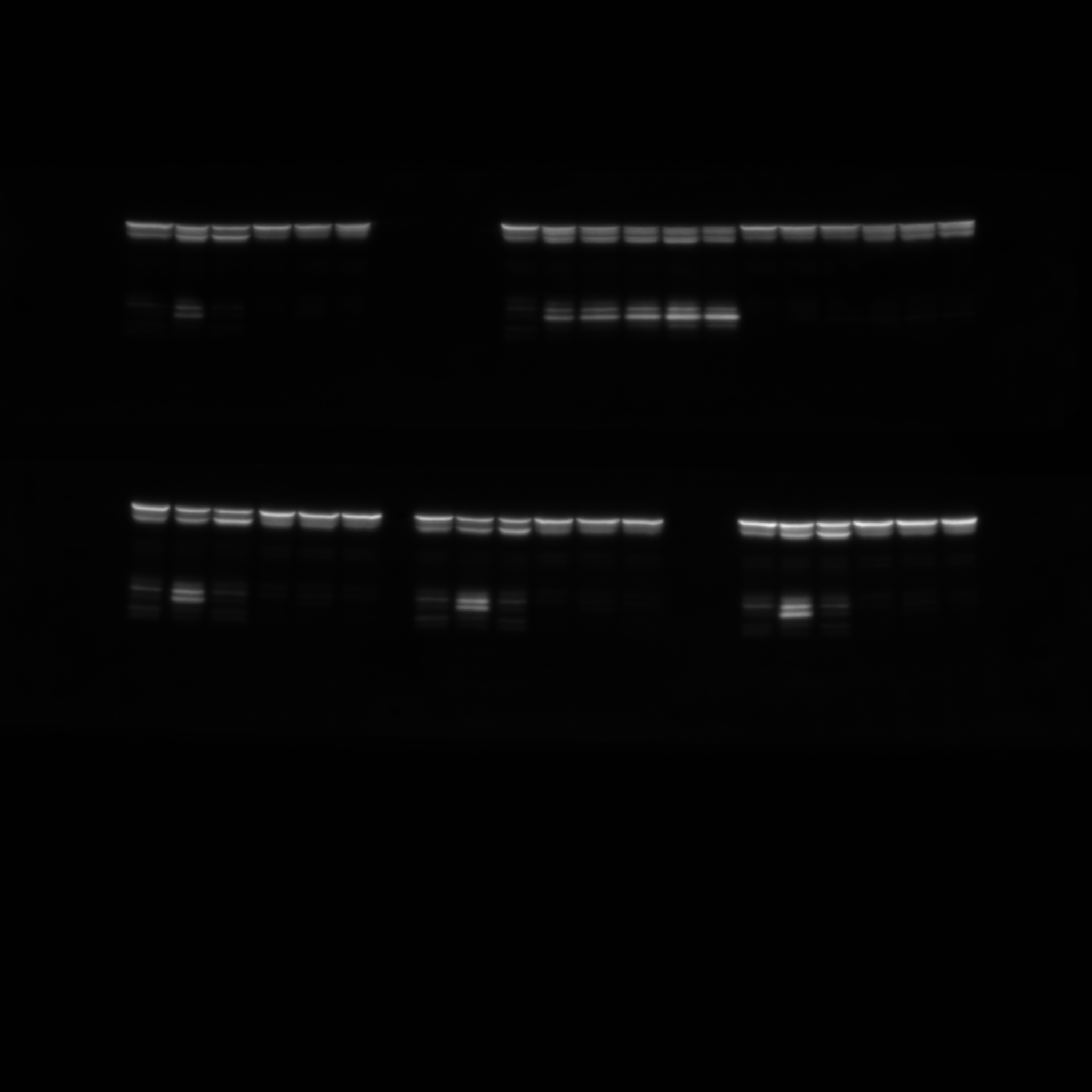

Supplement: Figure 1—source data 1. [file elife-78923-fig1-data1.zip › Figure 1-source data 1/Figure 1c_HaloTag blot_raw.Tif]

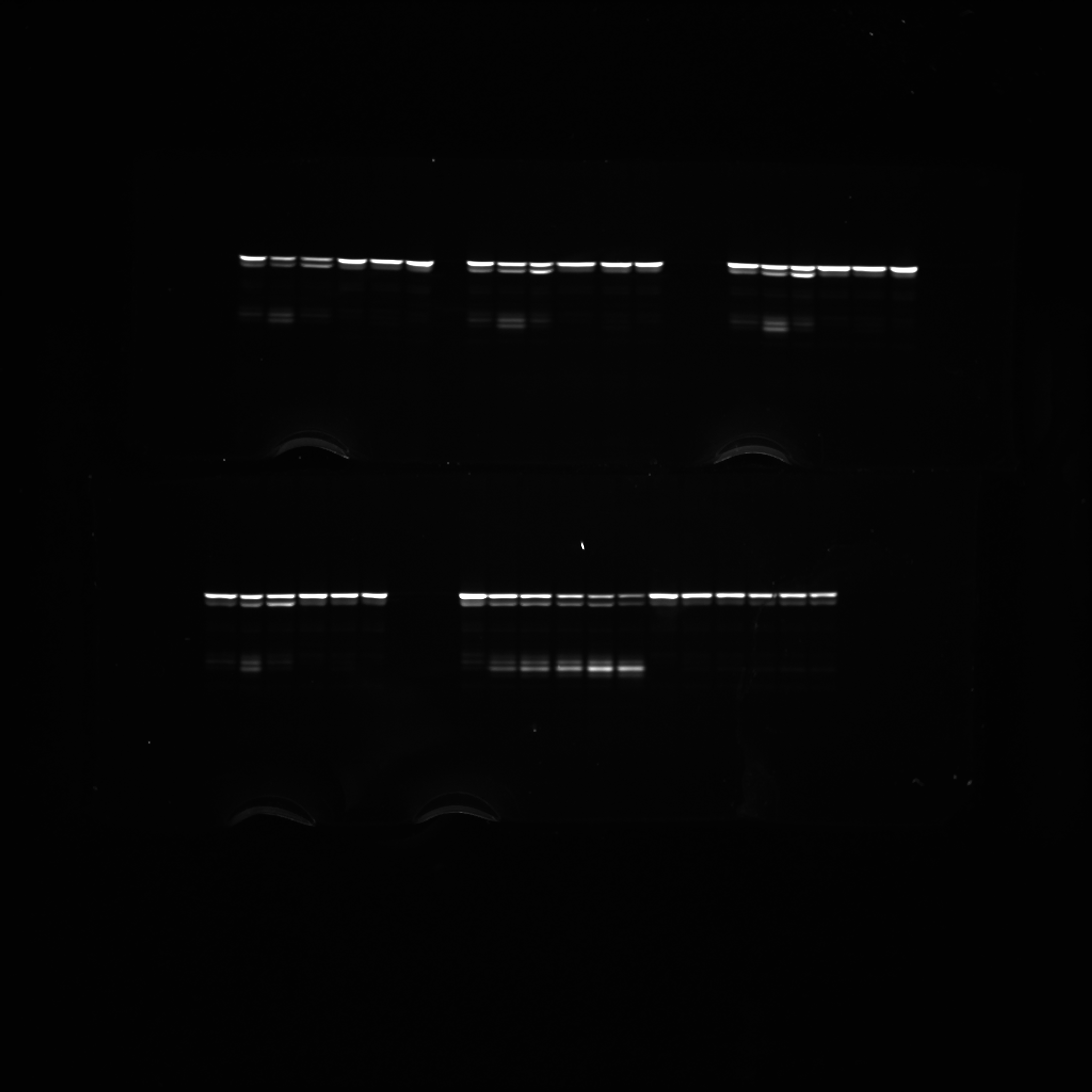

Supplement: Figure 1—source data 1. [file elife-78923-fig1-data1.zip › Figure 1-source data 1/Figure 1c_TMR in-gel fluorescence_raw.TIF]

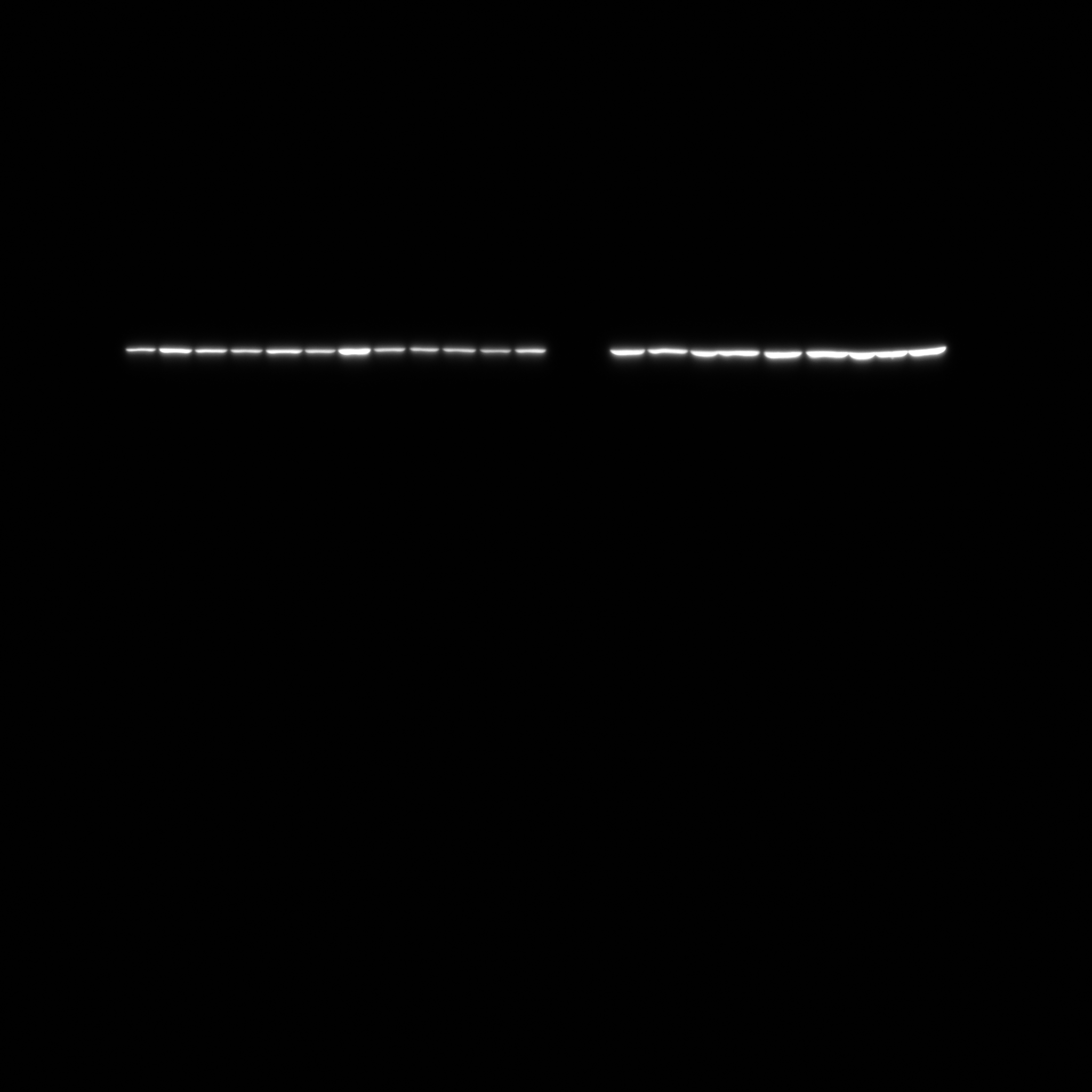

Supplement: Figure 1—source data 1. [file elife-78923-fig1-data1.zip › Figure 1-source data 1/Figure 1-S1_beta-actin blot_raw.Tif]

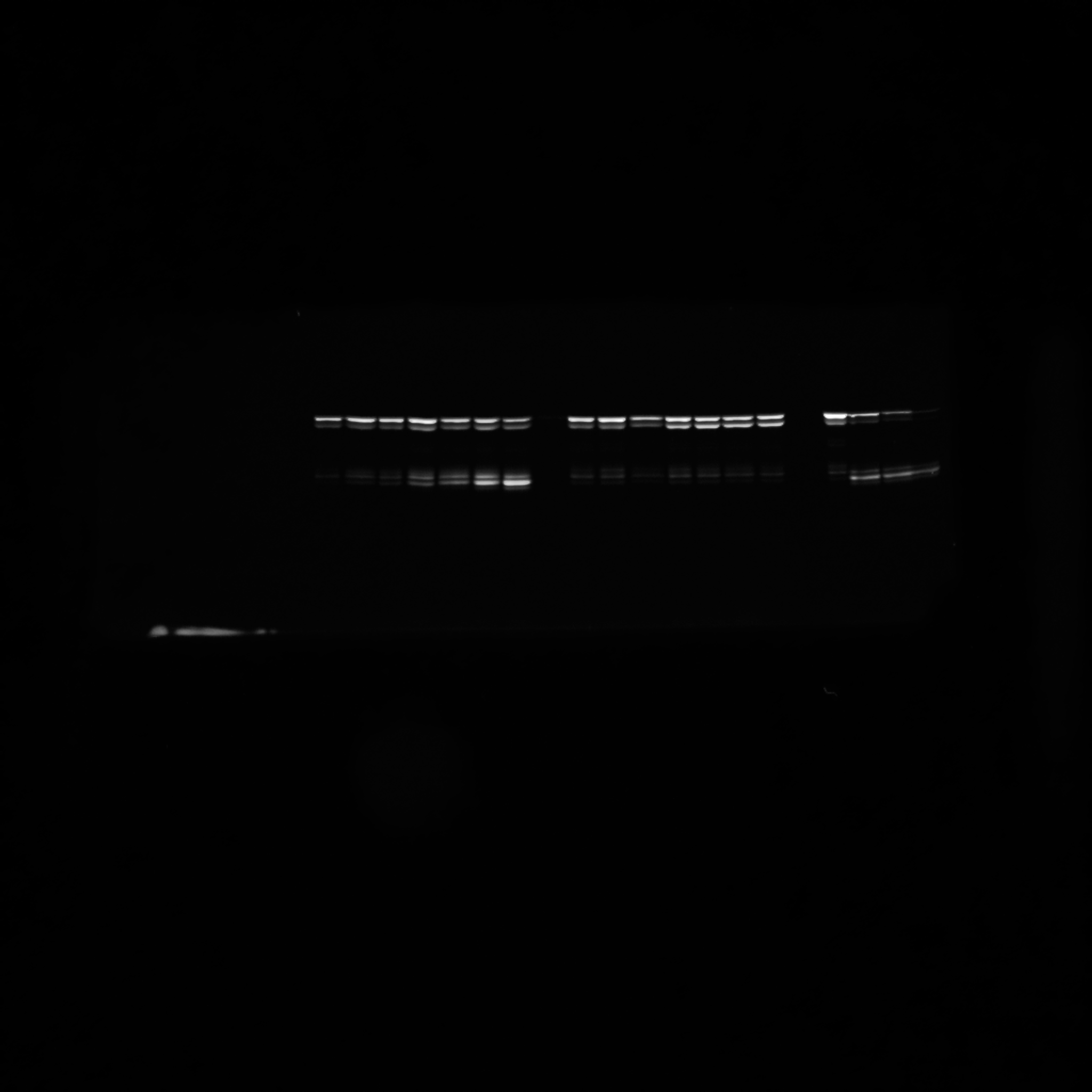

Supplement: Figure 1—source data 1. [file elife-78923-fig1-data1.zip › Figure 1-source data 1/Figure 1f_TMR in-gel fluorescence_raw.TIF]

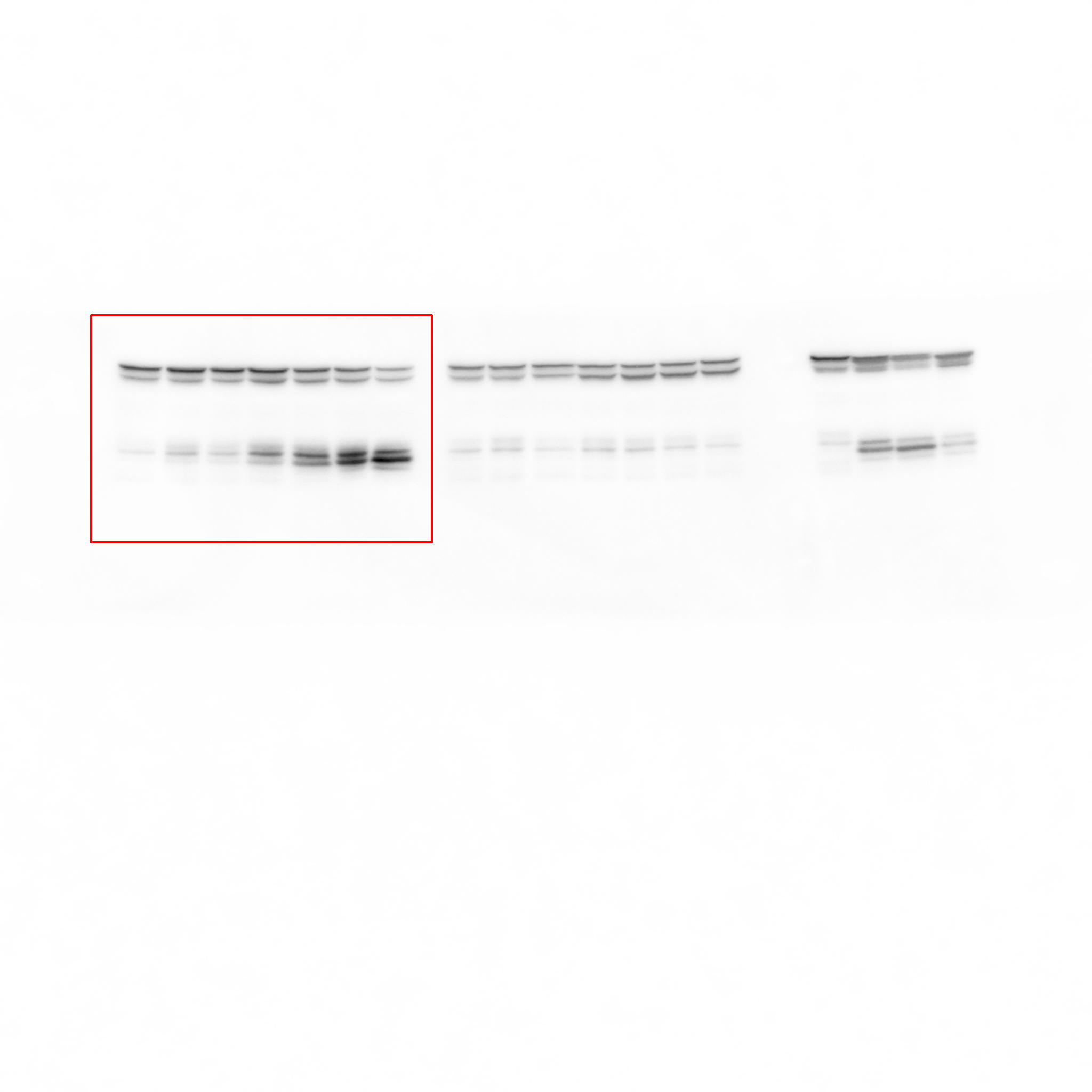

Supplement: Figure 1—source data 1. [file elife-78923-fig1-data1.zip › Figure 1-source data 1/Figure 1f_HaloTag blot_annotated.tif]

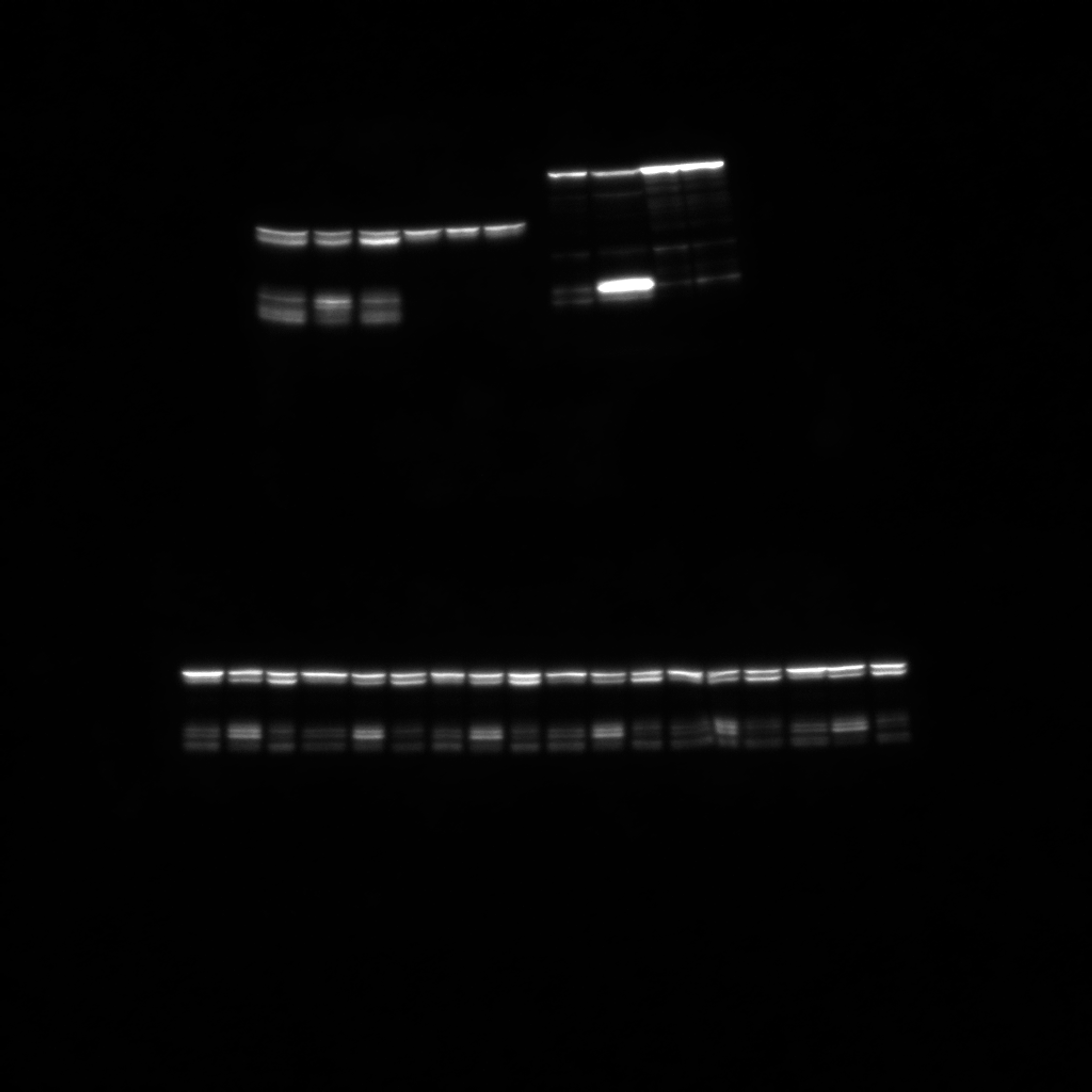

Supplement: Figure 2—figure supplement 1—source data 1. [file elife-78923-fig2-figsupp1-data1.zip › Figure 2-source data 1/Figure 2-S1e_MEF HaloTag blot_raw.Tif]

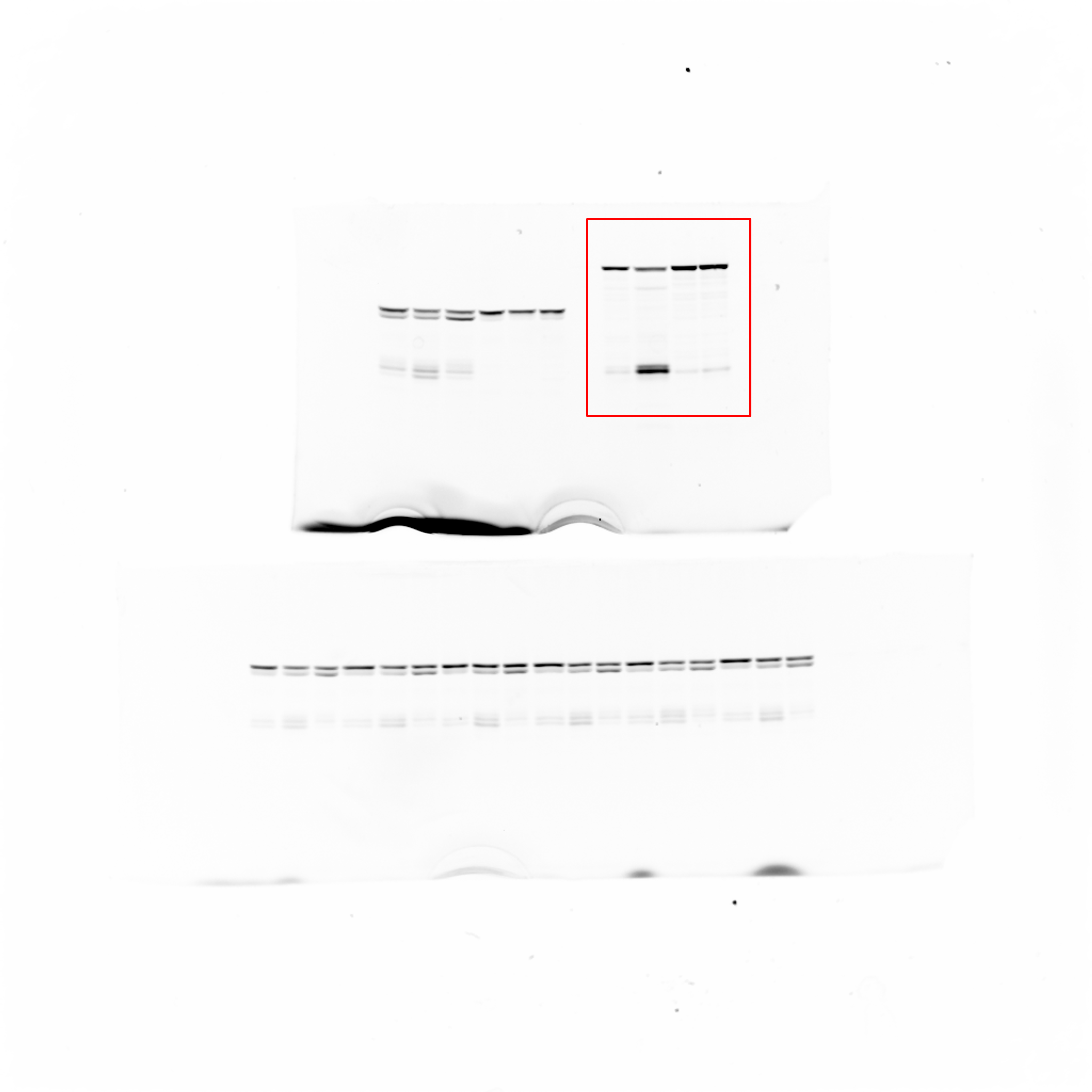

Supplement: Figure 2—figure supplement 1—source data 1. [file elife-78923-fig2-figsupp1-data1.zip › Figure 2-source data 1/Figure 2-S1e_MEF TMR in-gel fluorescence_annotated.tif]

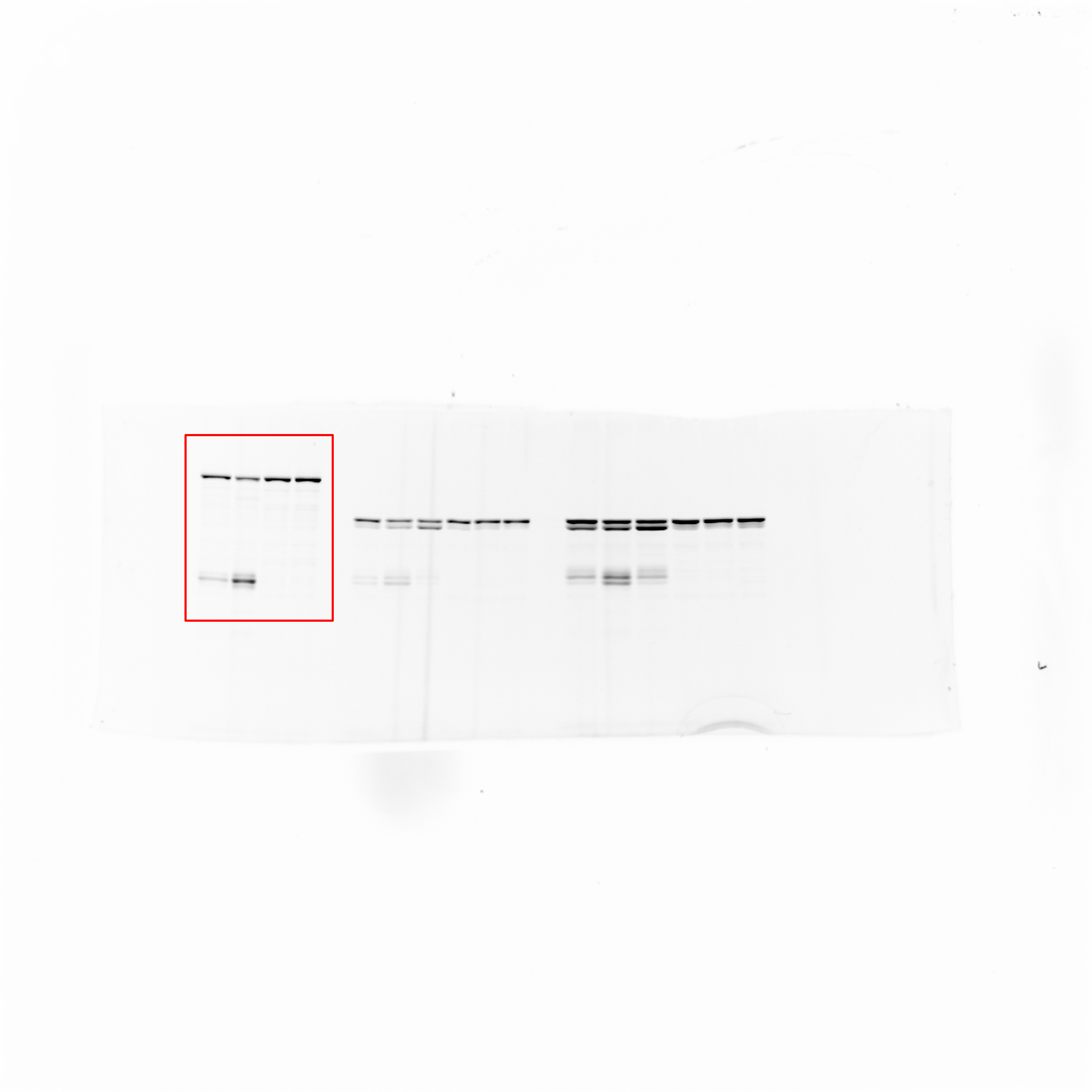

Supplement: Figure 2—figure supplement 1—source data 1. [file elife-78923-fig2-figsupp1-data1.zip › Figure 2-source data 1/Figure 2-S1e_HeLa TMR in-gel fluorescence_annotated.tif]

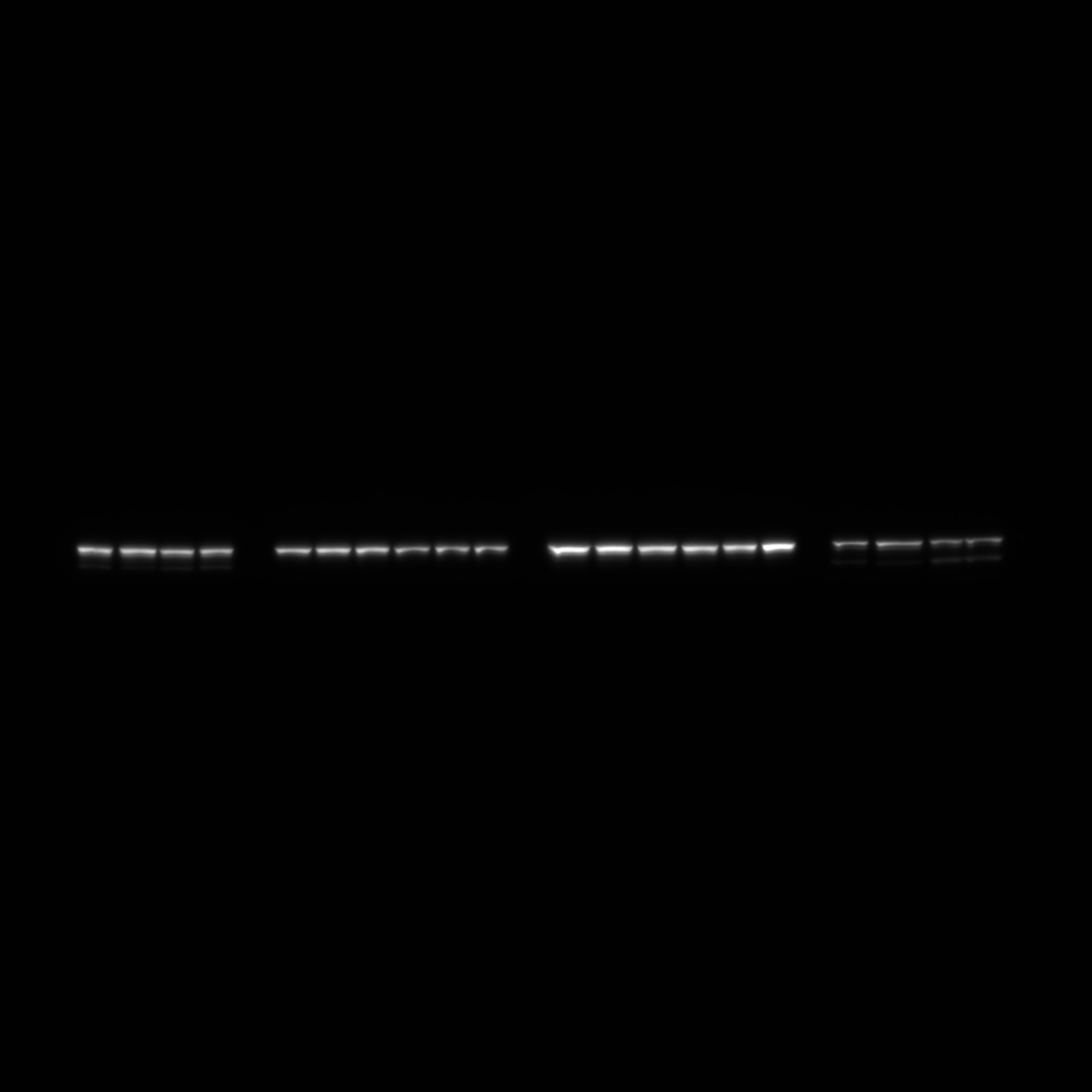

Supplement: Figure 2—figure supplement 1—source data 1. [file elife-78923-fig2-figsupp1-data1.zip › Figure 2-source data 1/Figure 2-S1e_HSP90 blot_raw.Tif]

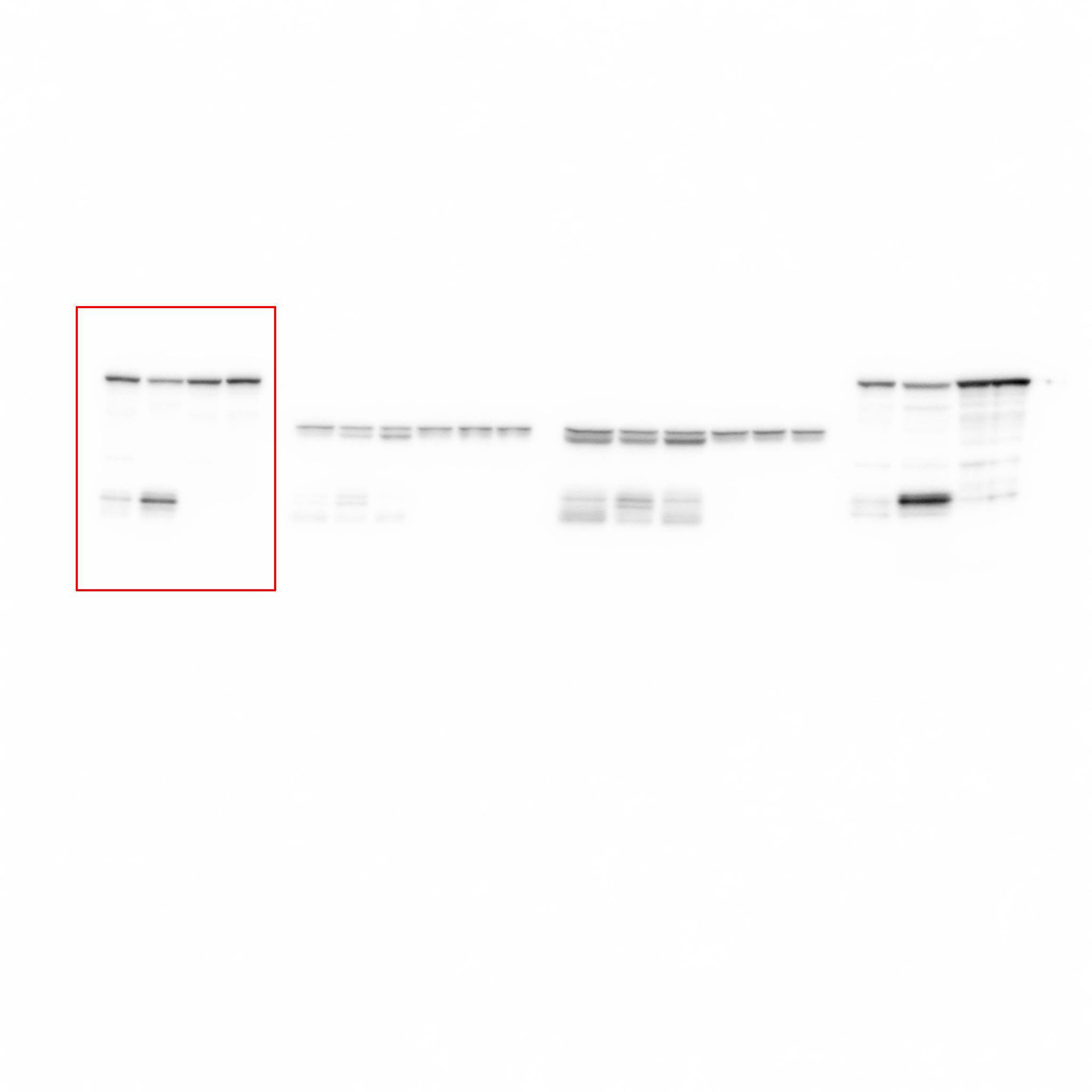

Supplement: Figure 2—figure supplement 1—source data 1. [file elife-78923-fig2-figsupp1-data1.zip › Figure 2-source data 1/Figure 2-S1e_HeLa HaloTag blot_annotated.tif]

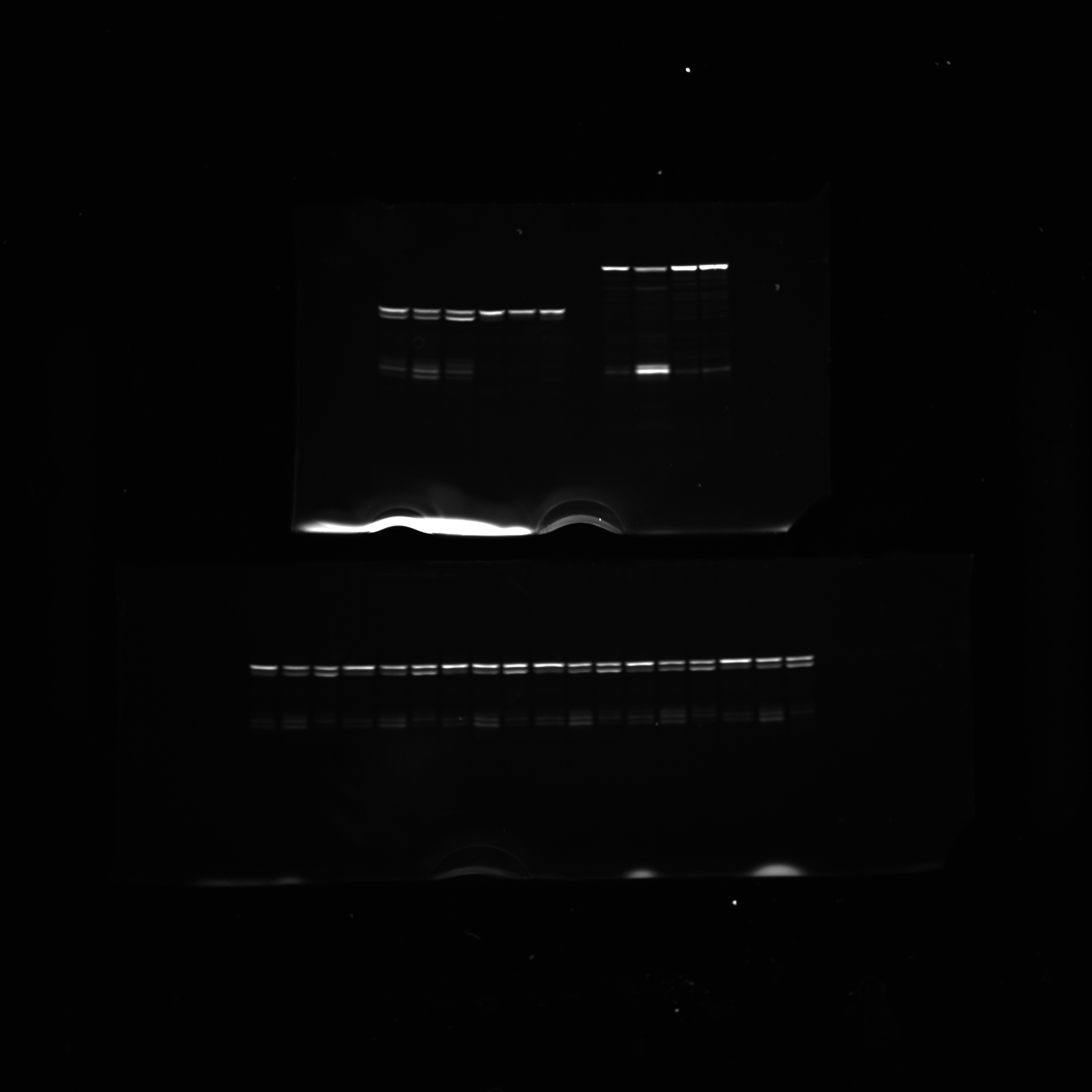

Supplement: Figure 2—figure supplement 1—source data 1. [file elife-78923-fig2-figsupp1-data1.zip › Figure 2-source data 1/Figure 2-S1e_MEF TMR in-gel fluorescence_raw.TIF]

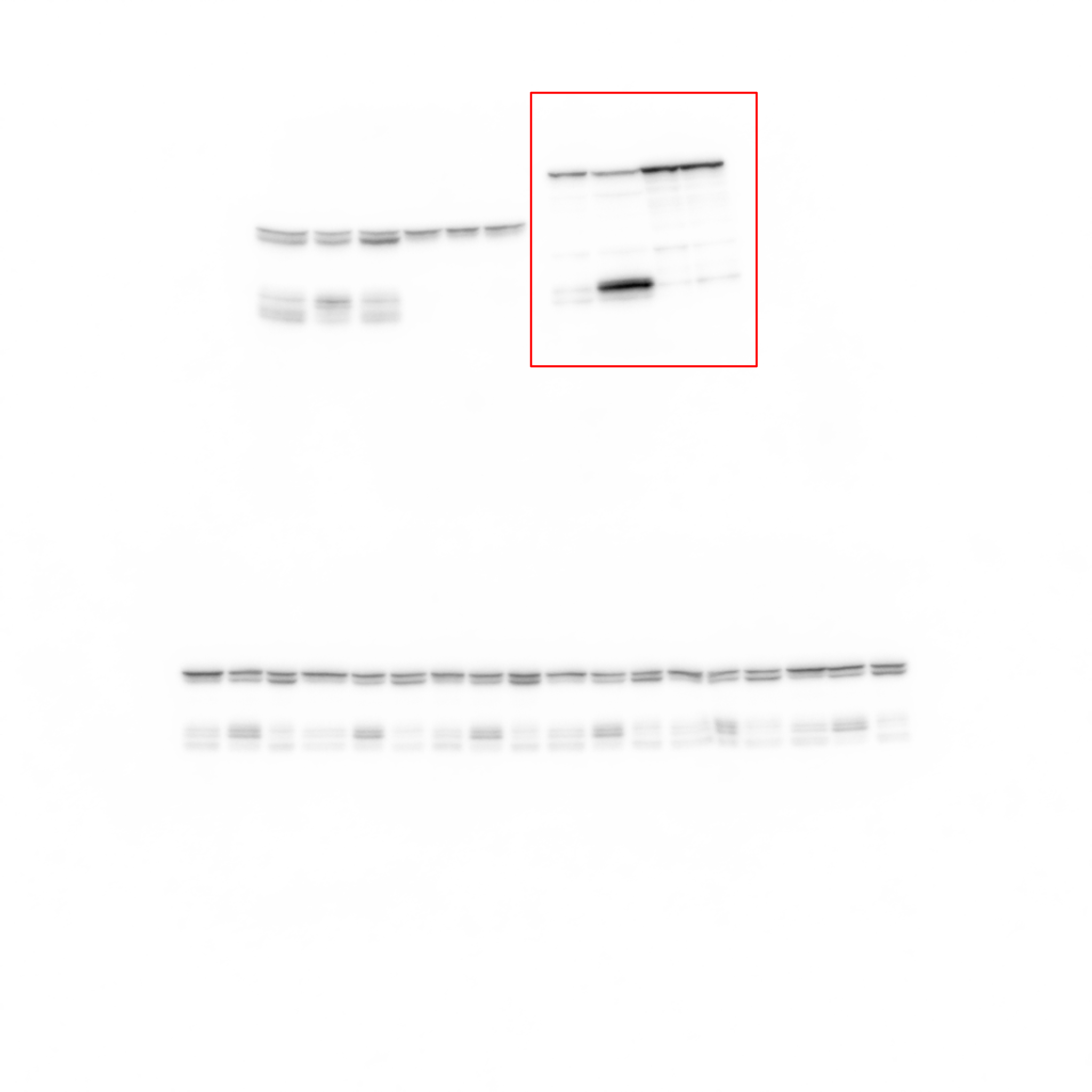

Supplement: Figure 2—figure supplement 1—source data 1. [file elife-78923-fig2-figsupp1-data1.zip › Figure 2-source data 1/Figure 2-S1e_MEF HaloTag blot_annotated.tif]

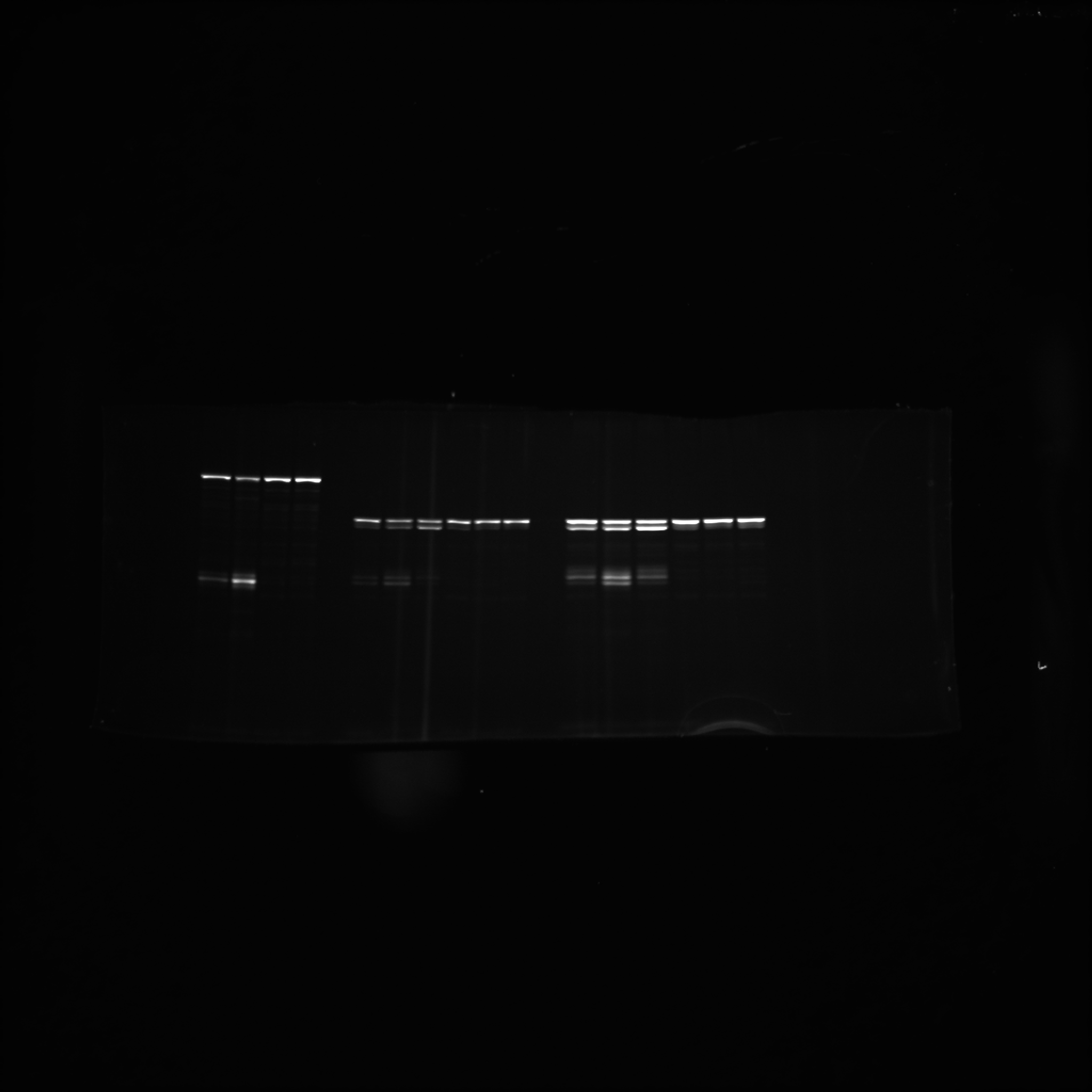

Supplement: Figure 2—figure supplement 1—source data 1. [file elife-78923-fig2-figsupp1-data1.zip › Figure 2-source data 1/Figure 2-S1e_HeLa TMR in-gel fluorescence_raw.TIF]

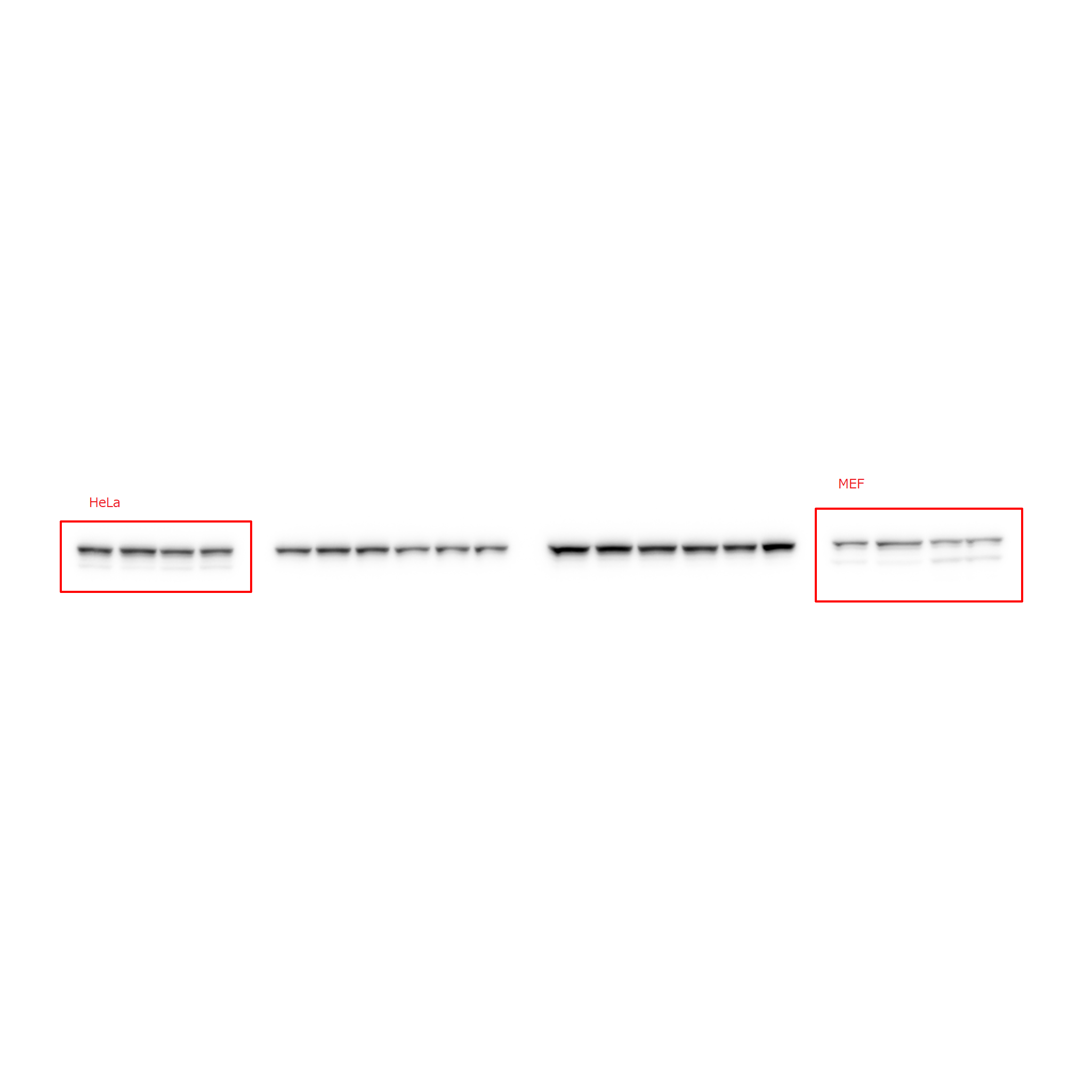

Supplement: Figure 2—figure supplement 1—source data 1. [file elife-78923-fig2-figsupp1-data1.zip › Figure 2-source data 1/Figure 2-S1e_HSP90 blot_annotated.tif]

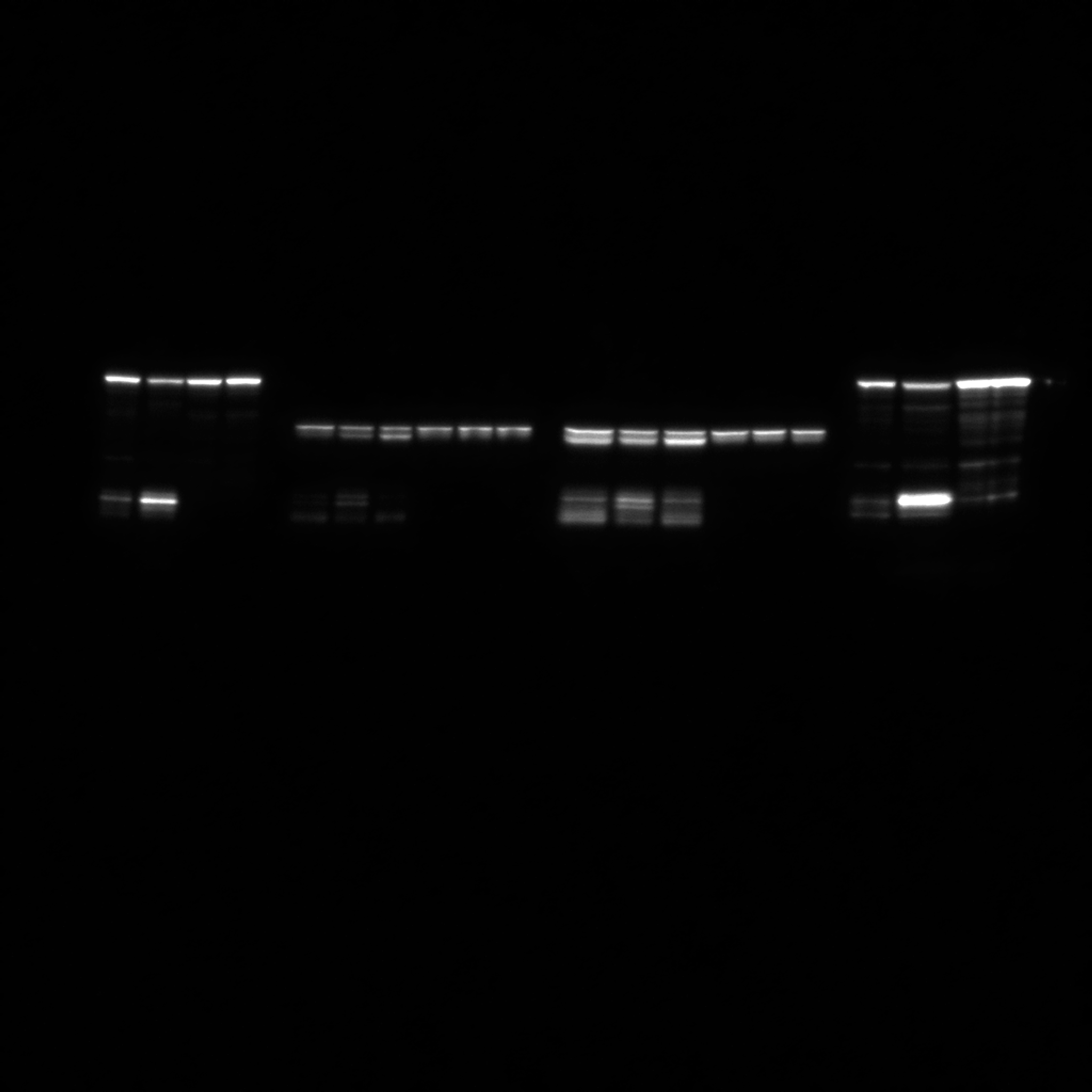

Supplement: Figure 2—figure supplement 1—source data 1. [file elife-78923-fig2-figsupp1-data1.zip › Figure 2-source data 1/Figure 2-S1e_HeLa HaloTag blot_raw.TIF]

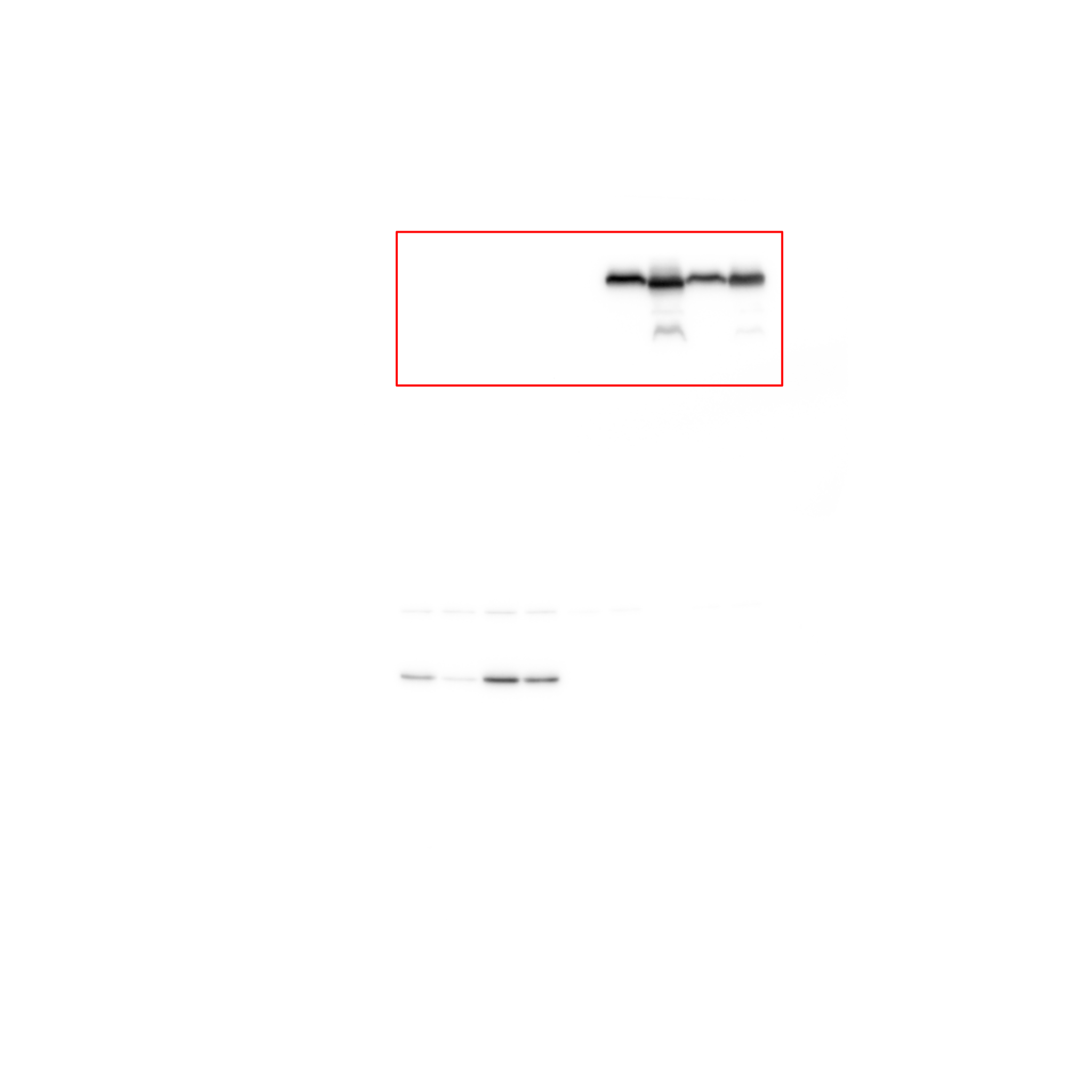

Supplement: Figure 3—source data 1. [file elife-78923-fig3-data1.zip › Figure 3-source data 1/Figure 3g_Parkin blot_annotated.tif]

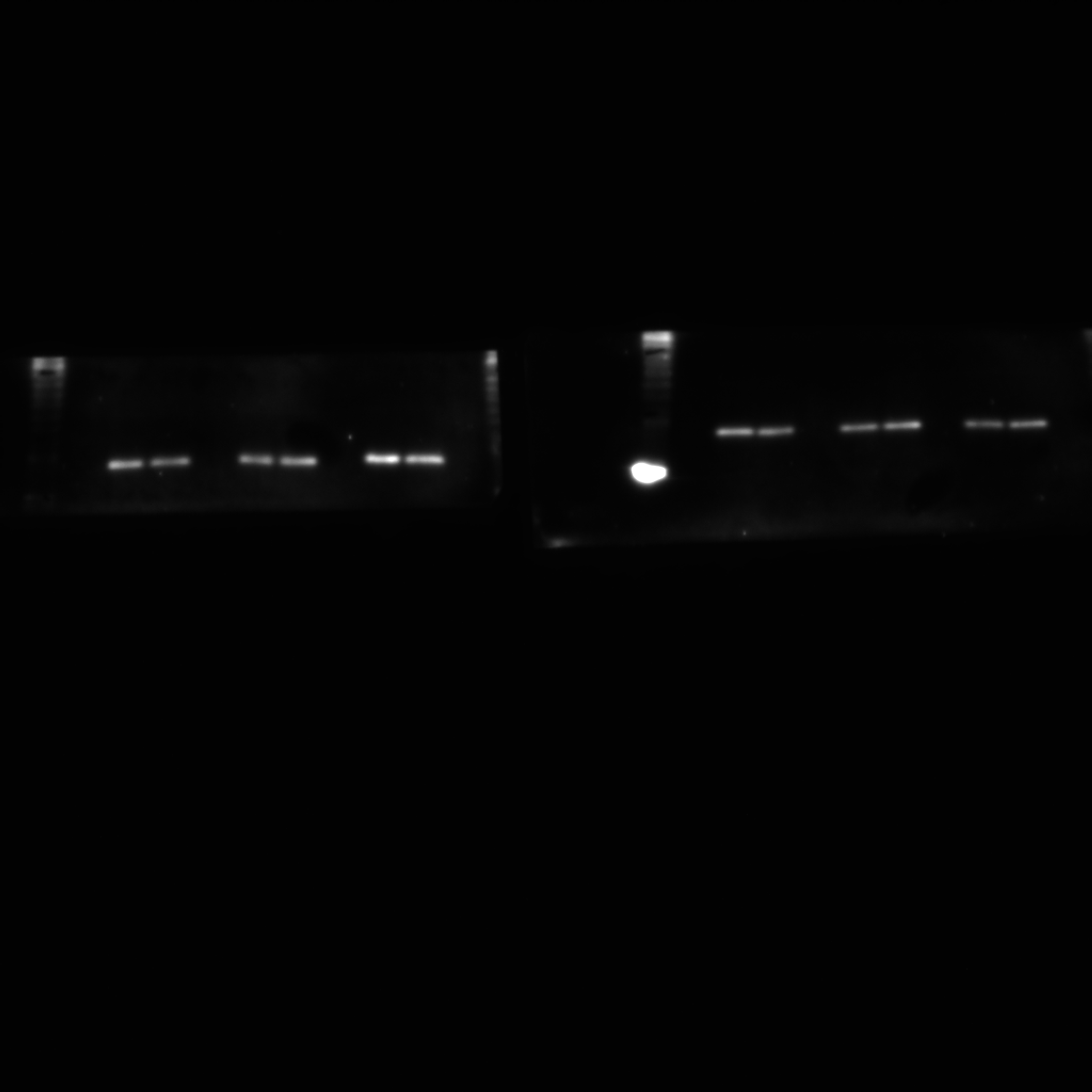

Supplement: Figure 3—source data 1. [file elife-78923-fig3-data1.zip › Figure 3-source data 1/Figure 3c_Sec61b blot_raw.TIF]

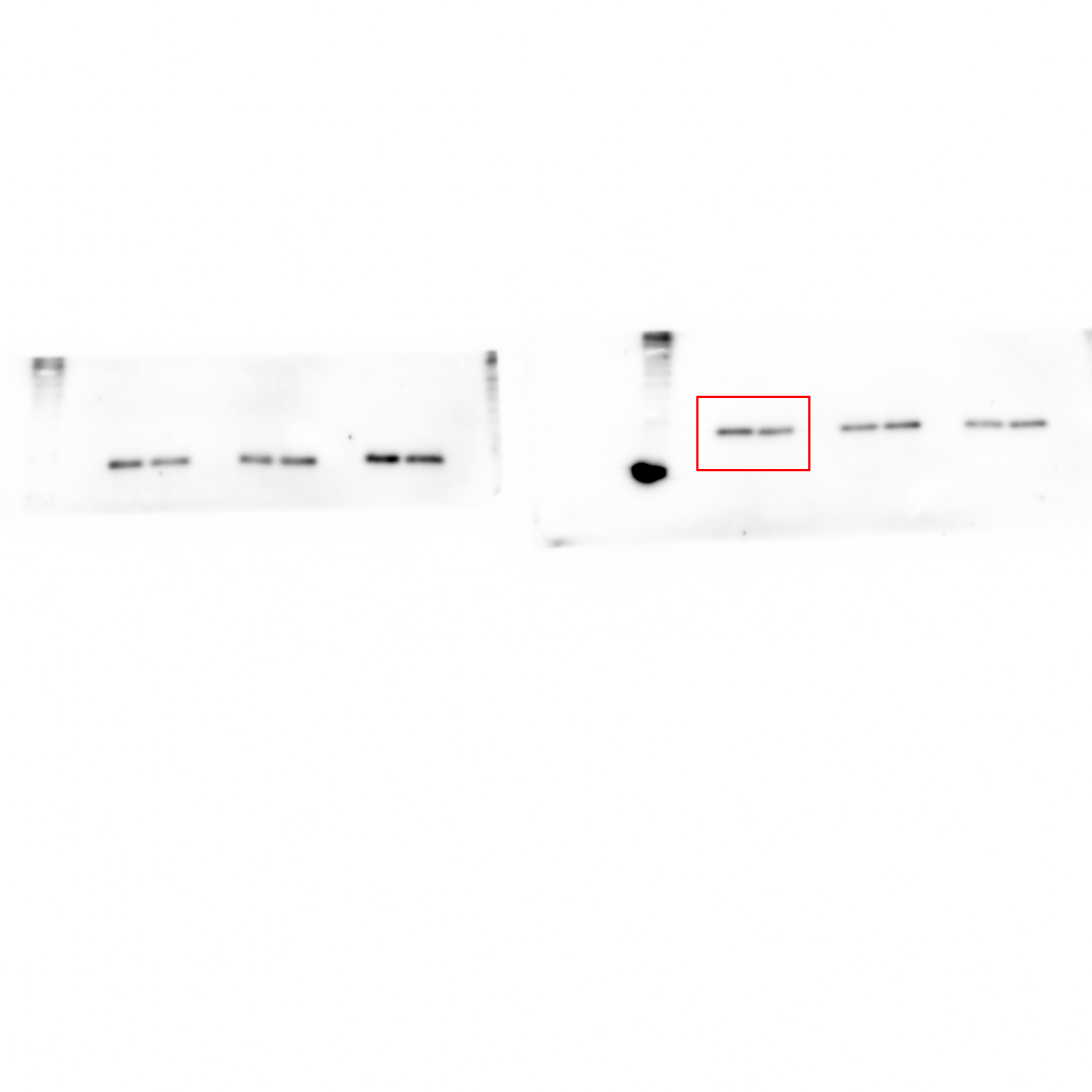

Supplement: Figure 3—source data 1. [file elife-78923-fig3-data1.zip › Figure 3-source data 1/Figure 3c_Sec61b blot_annotated.tif]

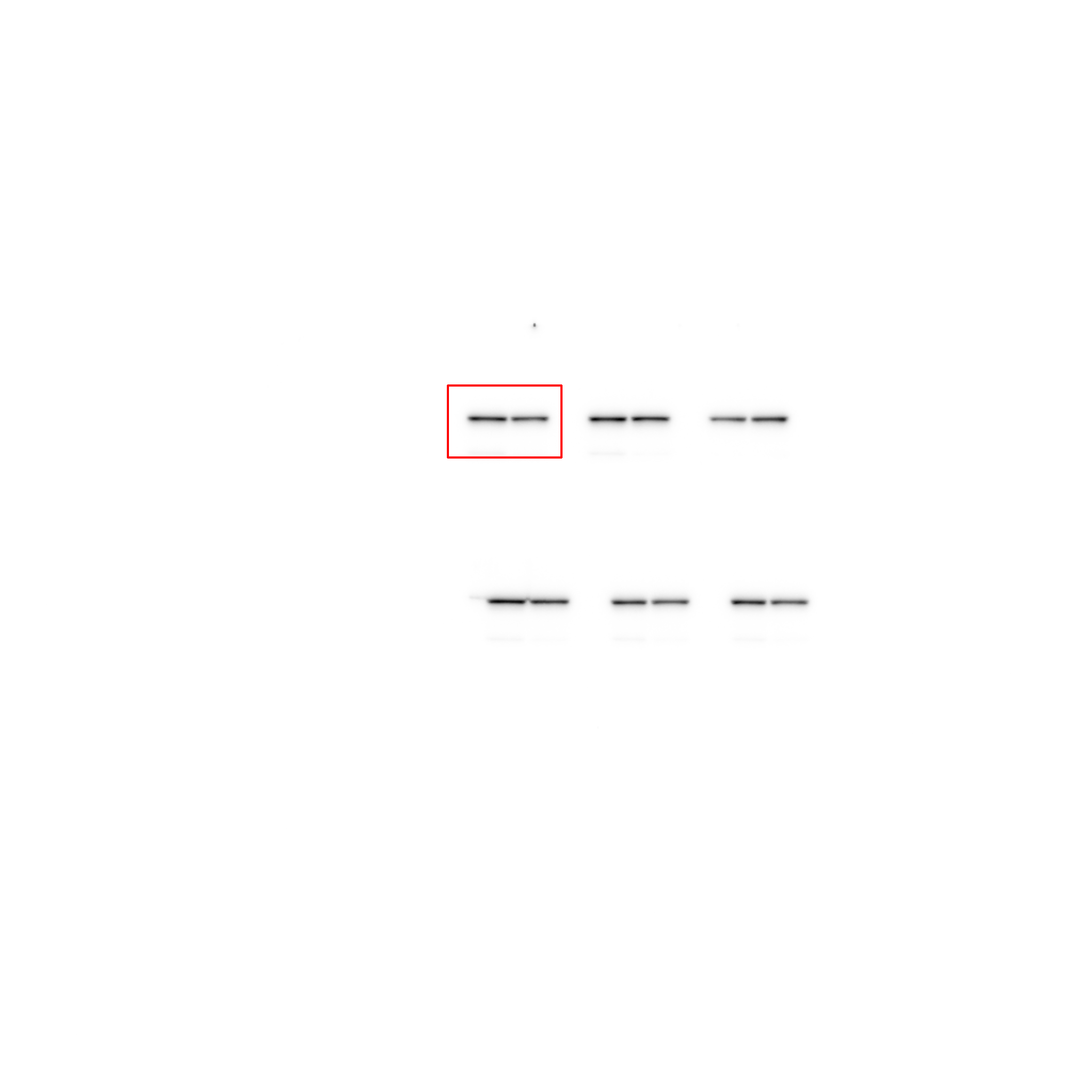

Supplement: Figure 3—source data 1. [file elife-78923-fig3-data1.zip › Figure 3-source data 1/Figure 3c_Calnexin blot_annotated.tif]

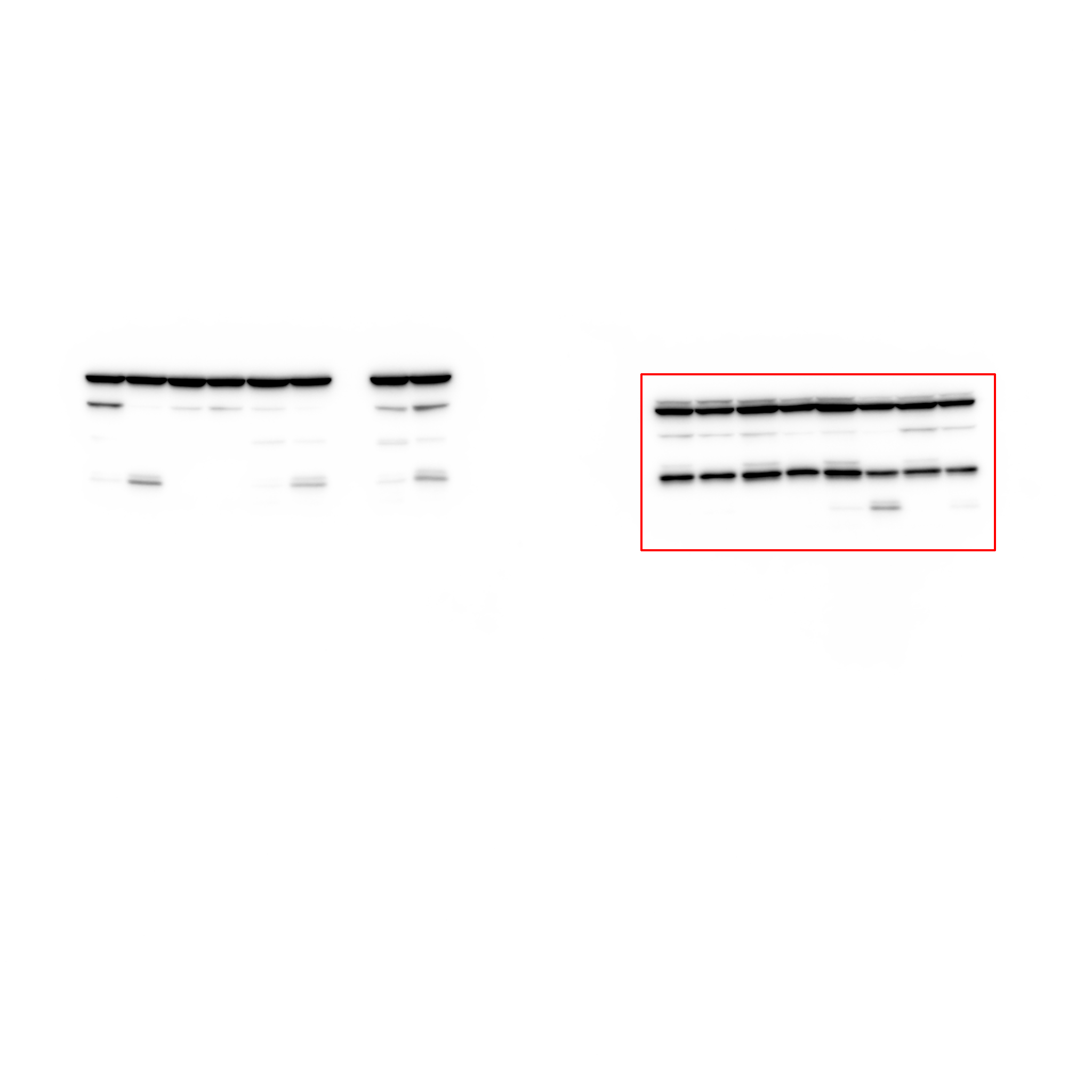

Supplement: Figure 3—source data 1. [file elife-78923-fig3-data1.zip › Figure 3-source data 1/Figure 3g_HaloTag blot_annotated.tif]

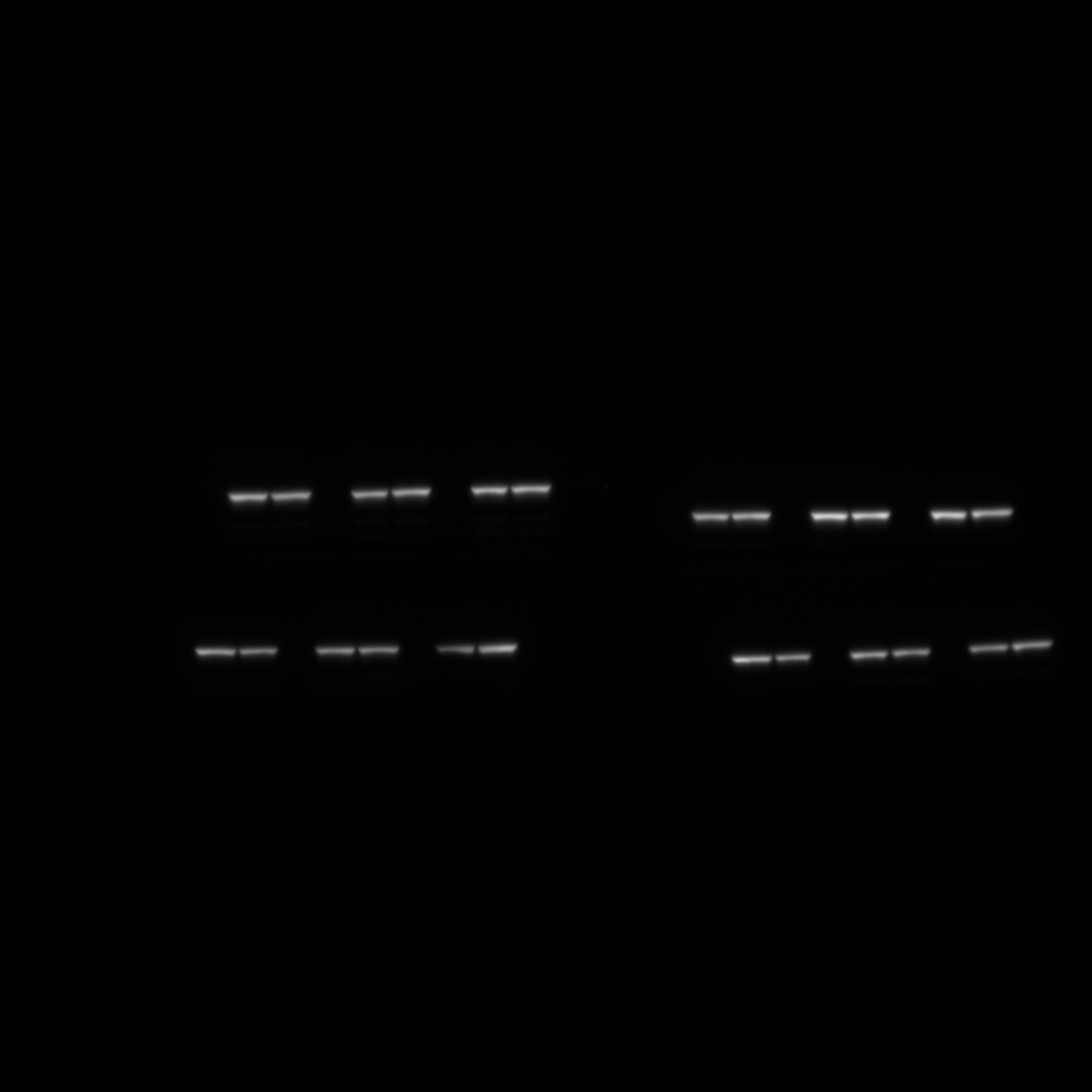

Supplement: Figure 3—source data 1. [file elife-78923-fig3-data1.zip › Figure 3-source data 1/Figure 3c_Hsp90 blot_raw.Tif]

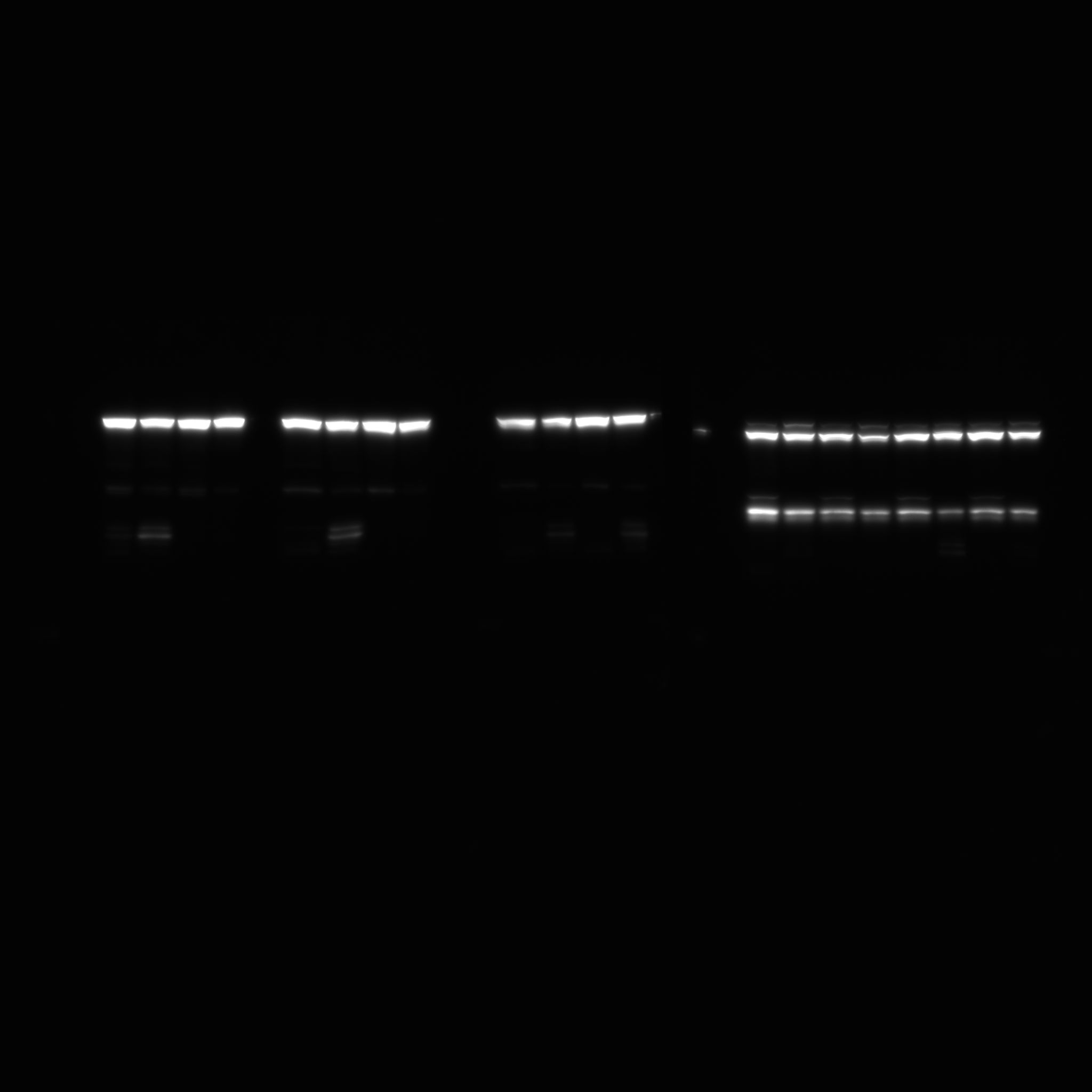

Supplement: Figure 3—source data 1. [file elife-78923-fig3-data1.zip › Figure 3-source data 1/Figure 3a_HaloTag blot_raw.TIF]

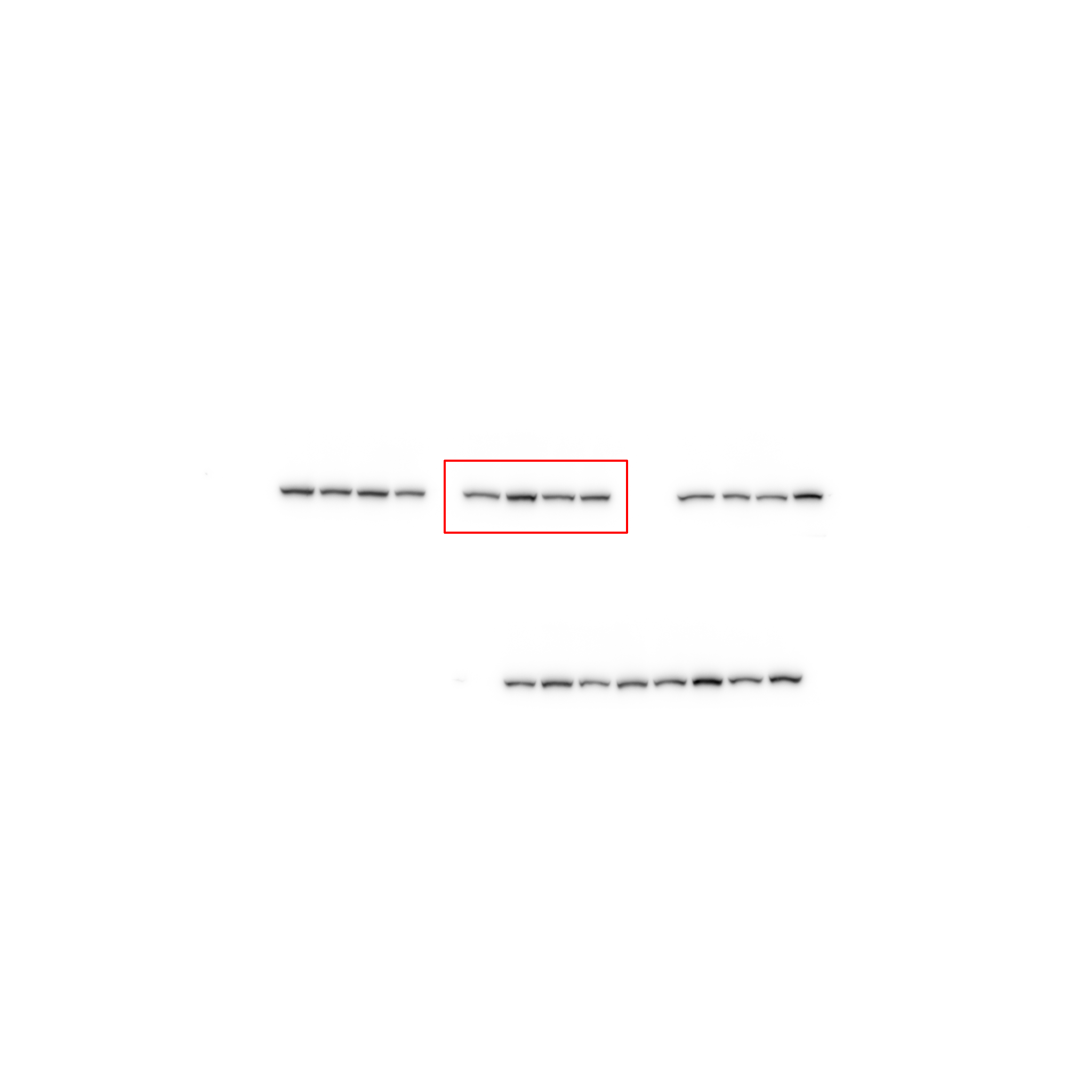

Supplement: Figure 3—source data 1. [file elife-78923-fig3-data1.zip › Figure 3-source data 1/Figure 3a_Hsp90 blot_annotated.tif]

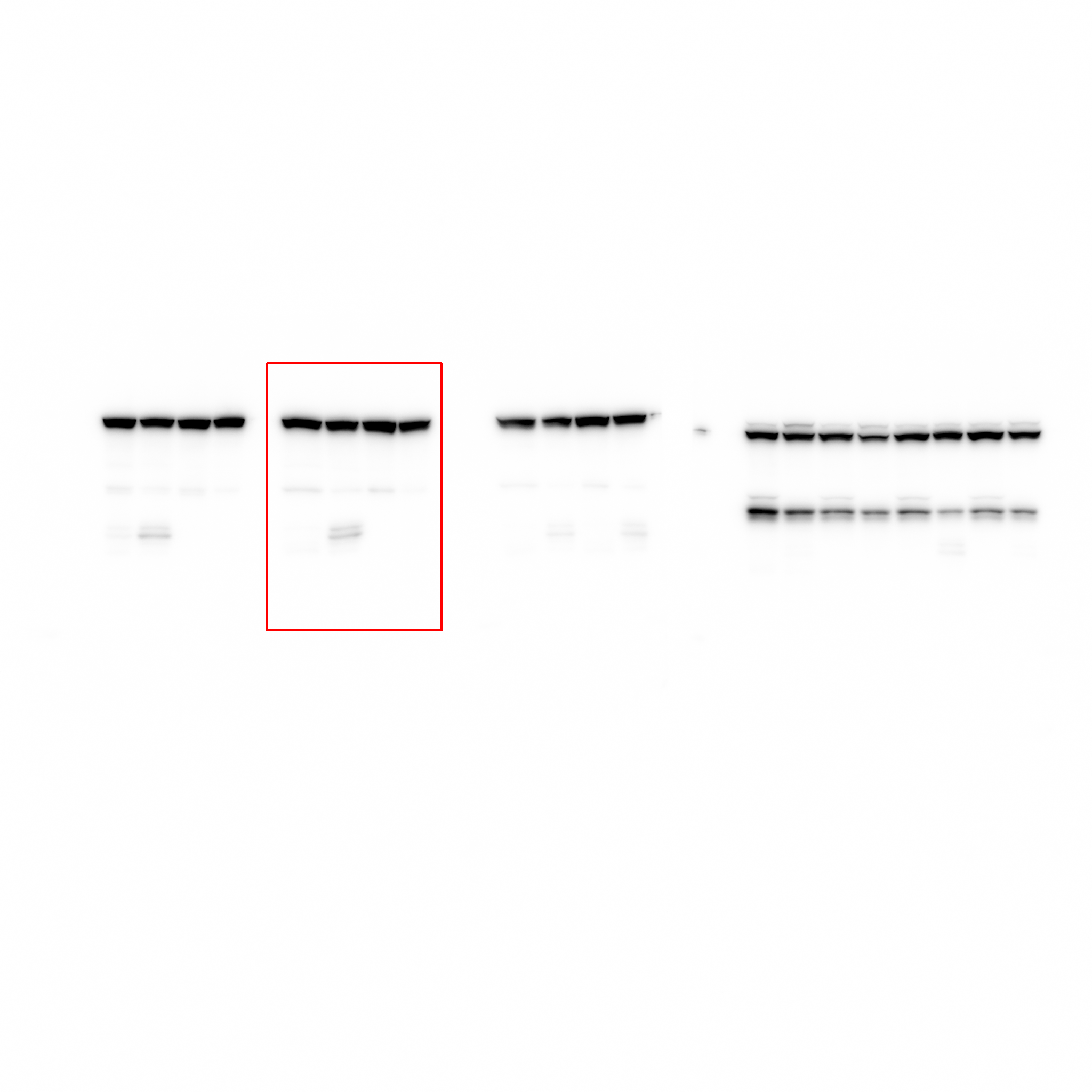

Supplement: Figure 3—source data 1. [file elife-78923-fig3-data1.zip › Figure 3-source data 1/Figure 3a_HaloTag blot_annotated.tif]

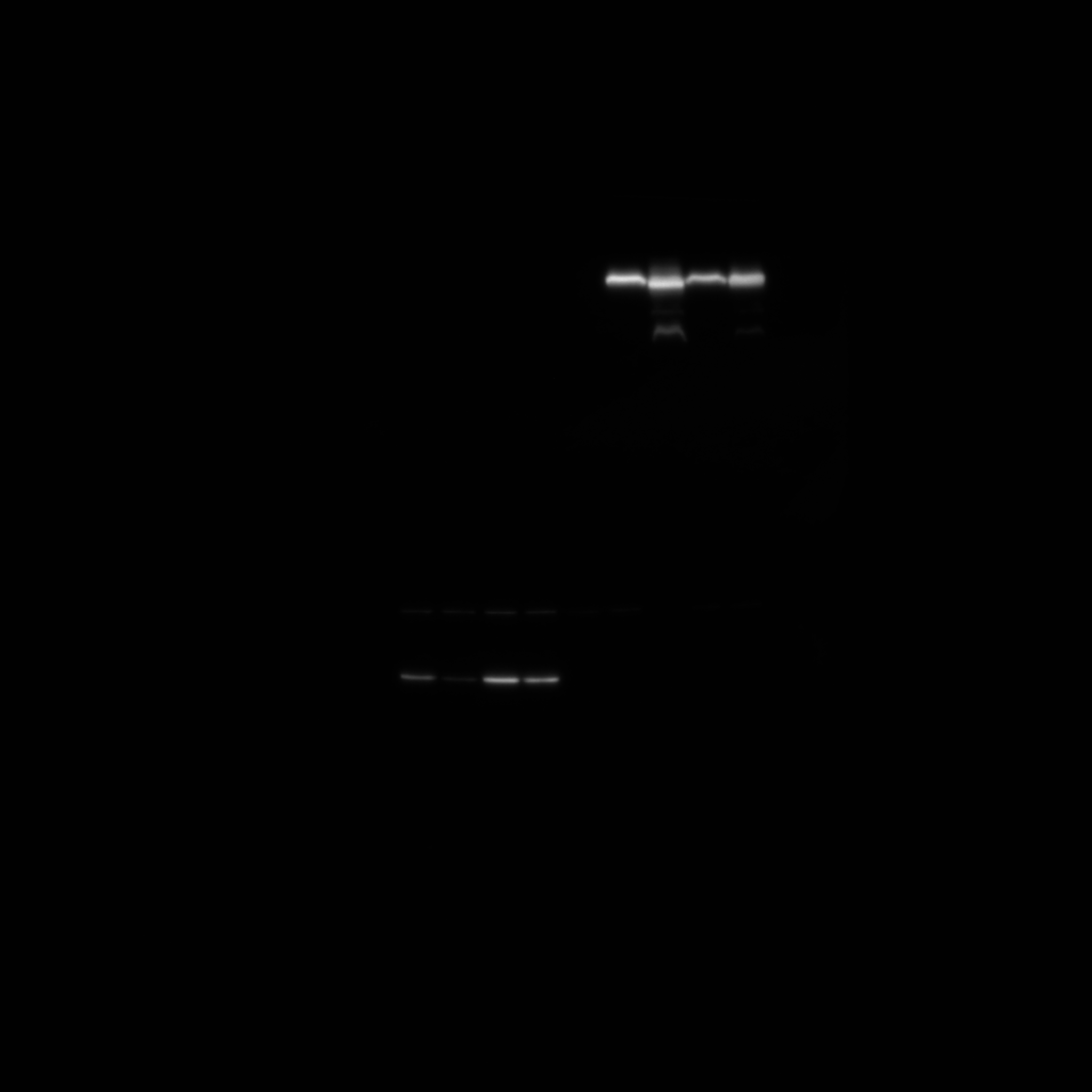

Supplement: Figure 3—source data 1. [file elife-78923-fig3-data1.zip › Figure 3-source data 1/Figure 3g_Parkin blot_raw.Tif]

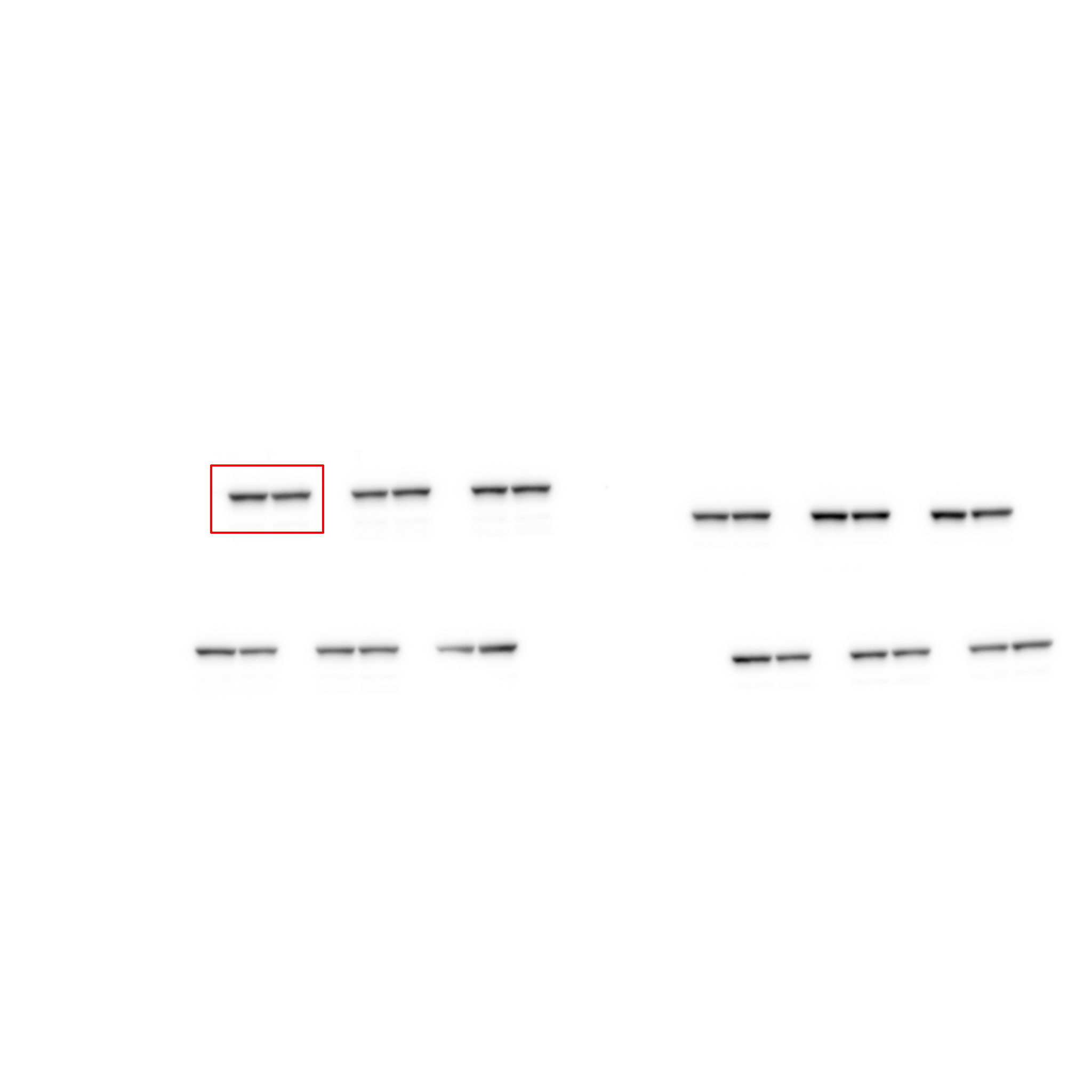

Supplement: Figure 3—source data 1. [file elife-78923-fig3-data1.zip › Figure 3-source data 1/Figure 3c_Hsp90 blot_annotated.tif]

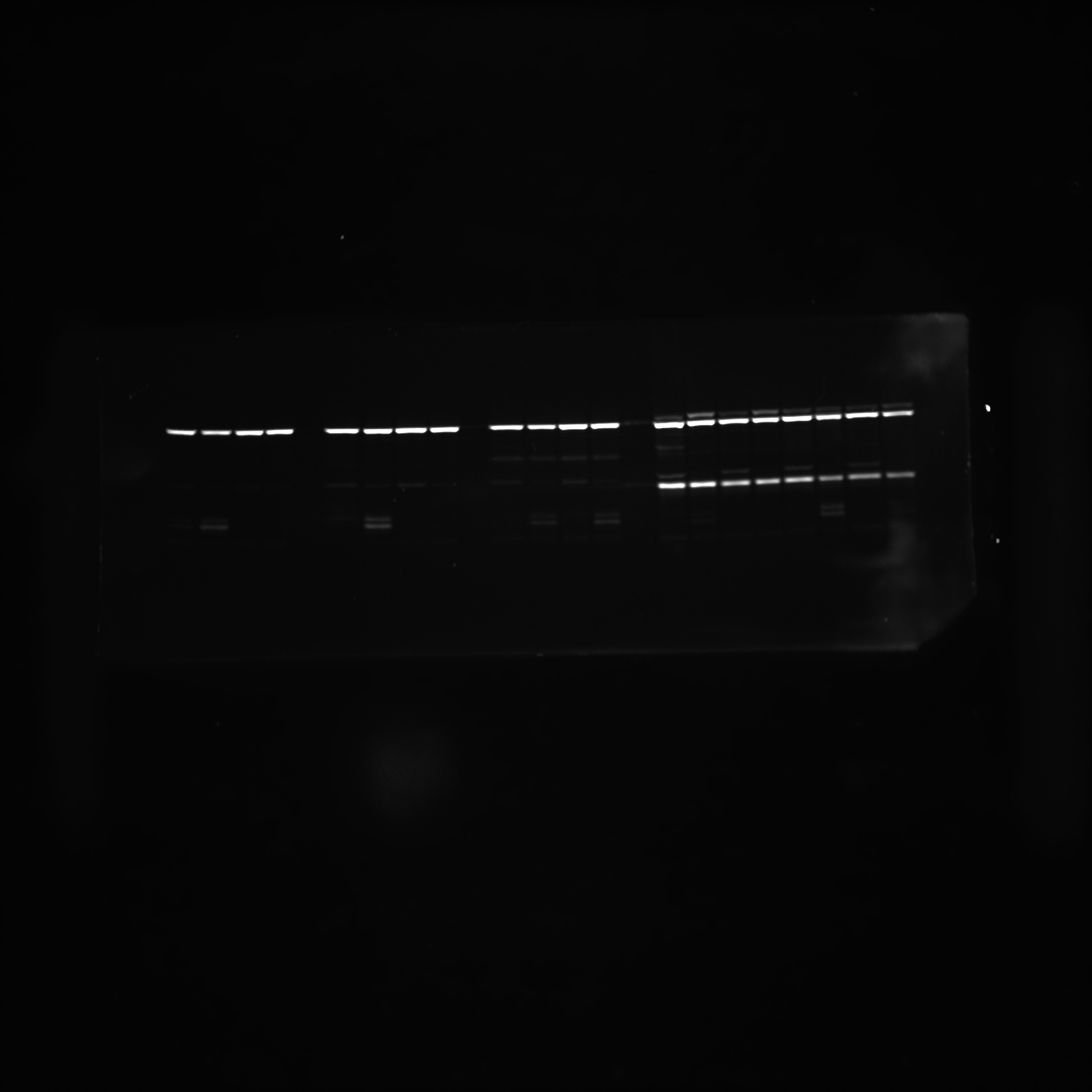

Supplement: Figure 3—source data 1. [file elife-78923-fig3-data1.zip › Figure 3-source data 1/Figure 3a_TMR in-gel fluorescence_raw.TIF]

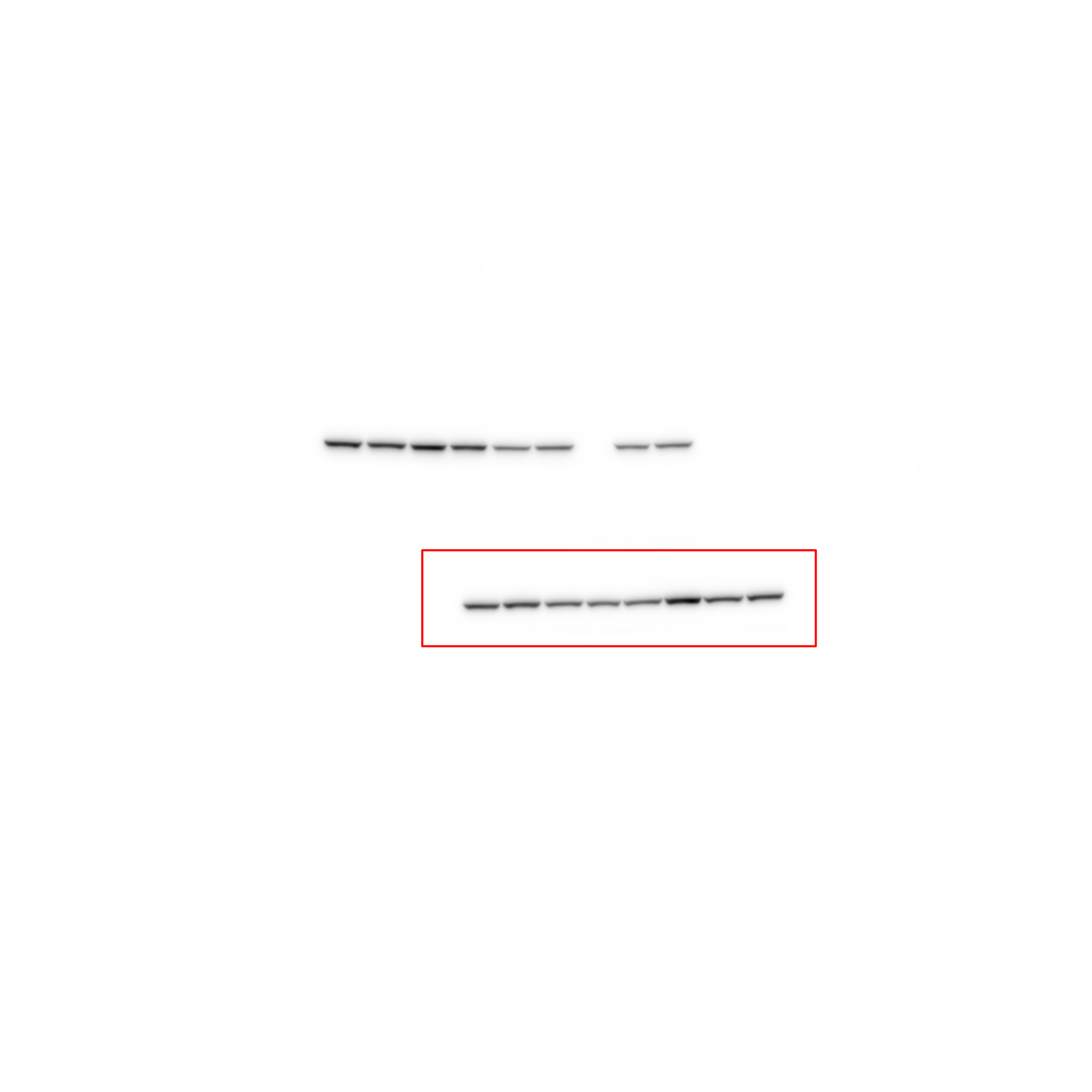

Supplement: Figure 3—source data 1. [file elife-78923-fig3-data1.zip › Figure 3-source data 1/Figure 3g_Hsp90 blot_annotated.tif]

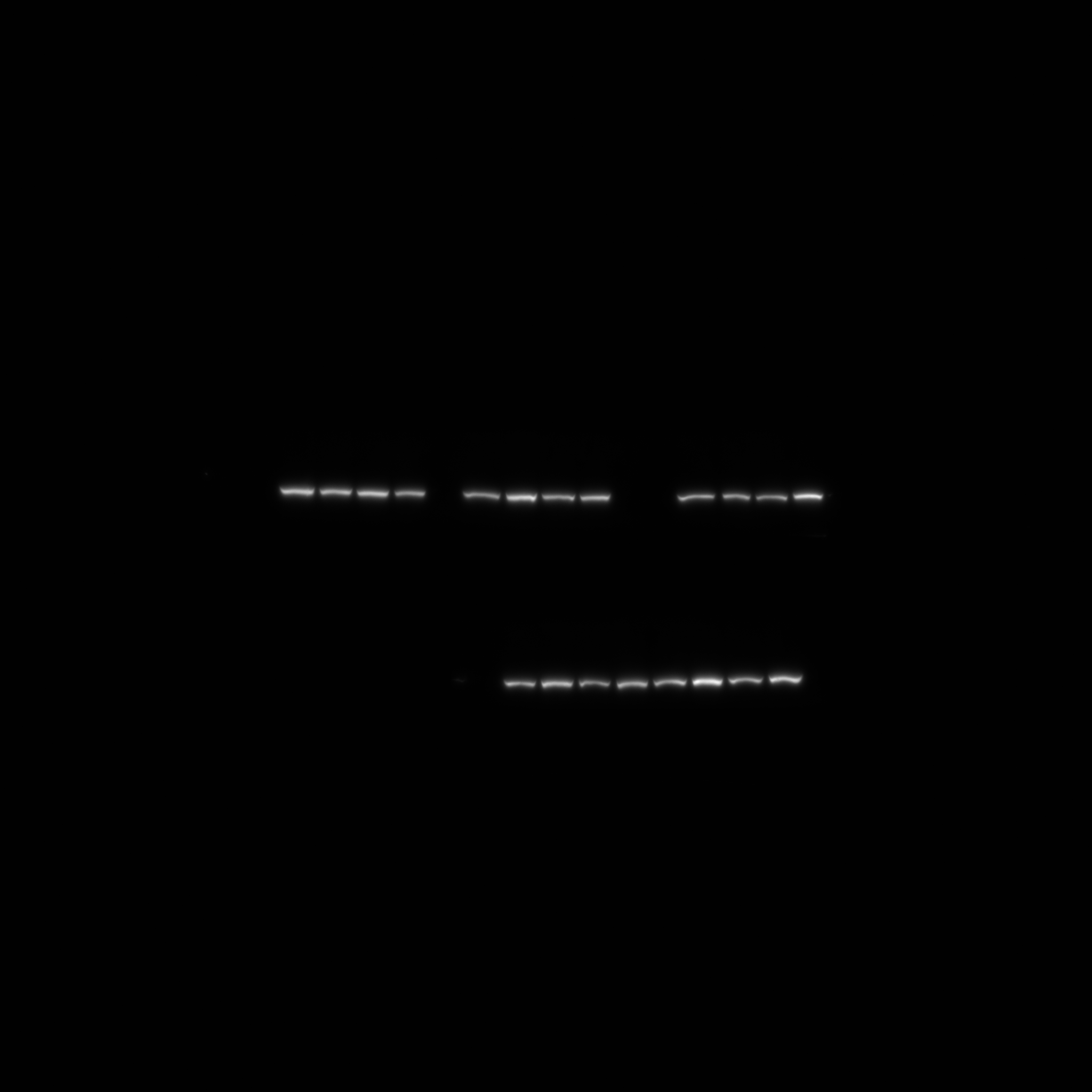

Supplement: Figure 3—source data 1. [file elife-78923-fig3-data1.zip › Figure 3-source data 1/Figure 3a_Hsp90 blot_raw.Tif]

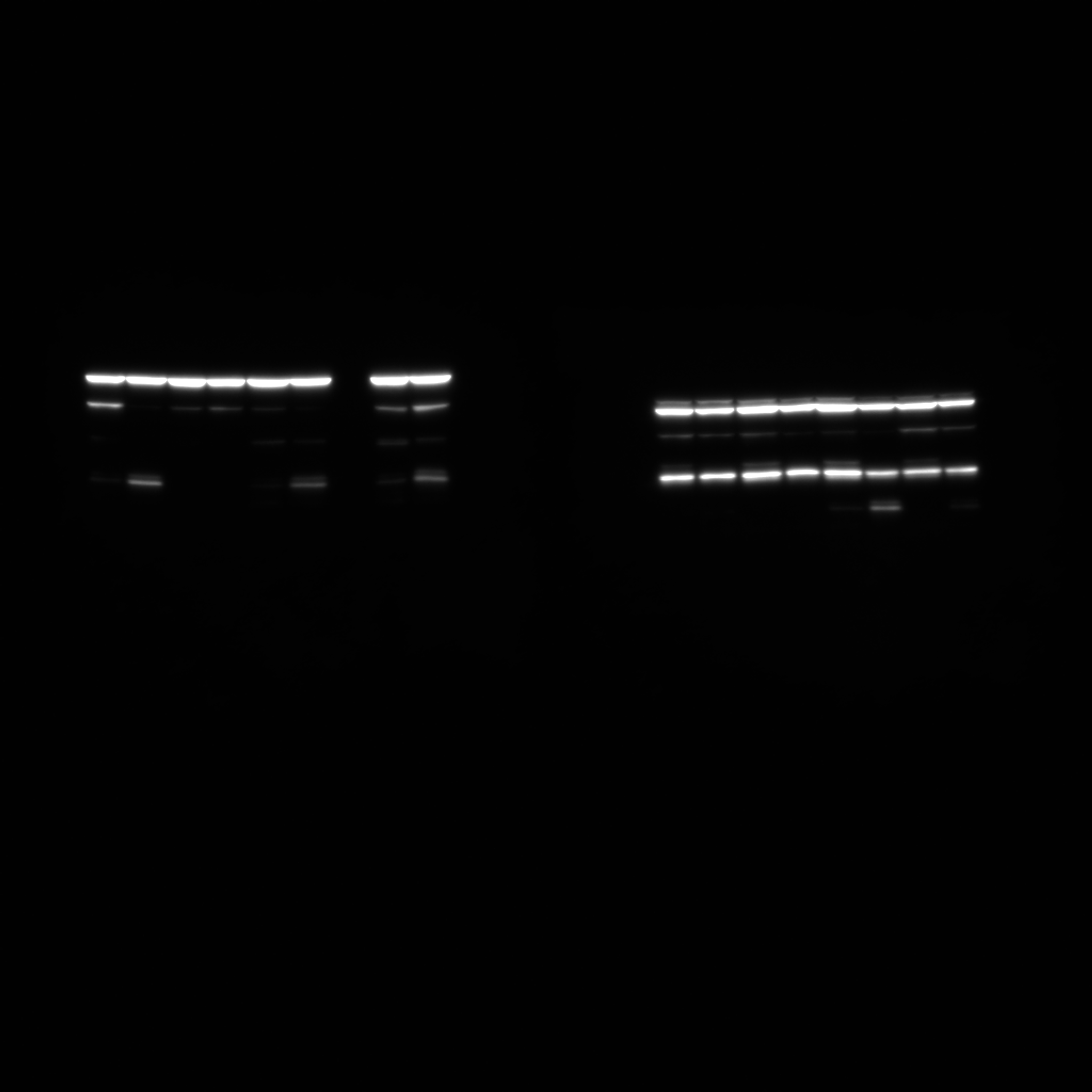

Supplement: Figure 3—source data 1. [file elife-78923-fig3-data1.zip › Figure 3-source data 1/Figure 3g_HaloTag blot_raw.TIF]

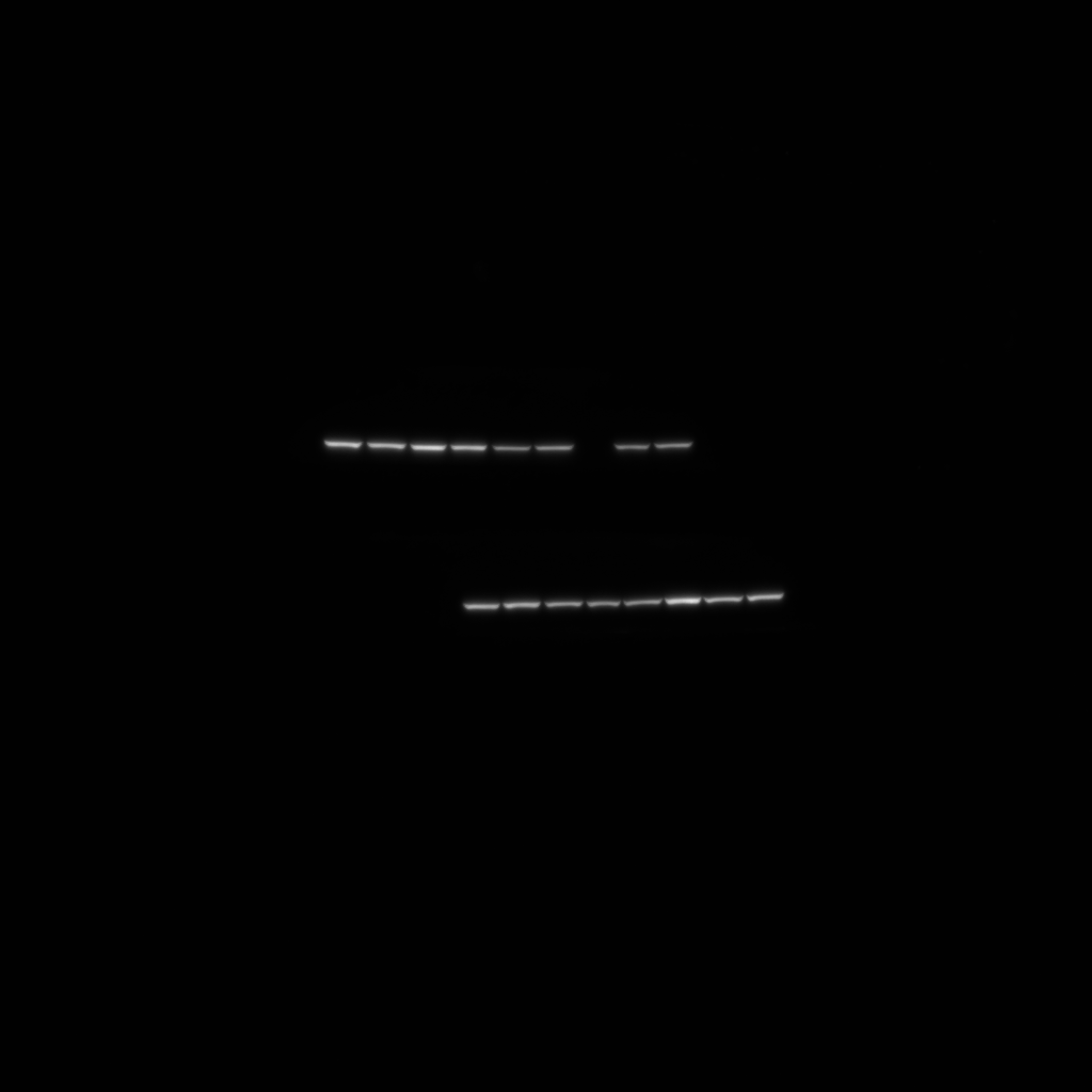

Supplement: Figure 3—source data 1. [file elife-78923-fig3-data1.zip › Figure 3-source data 1/Figure 3g_Hsp90 blot_raw.Tif]

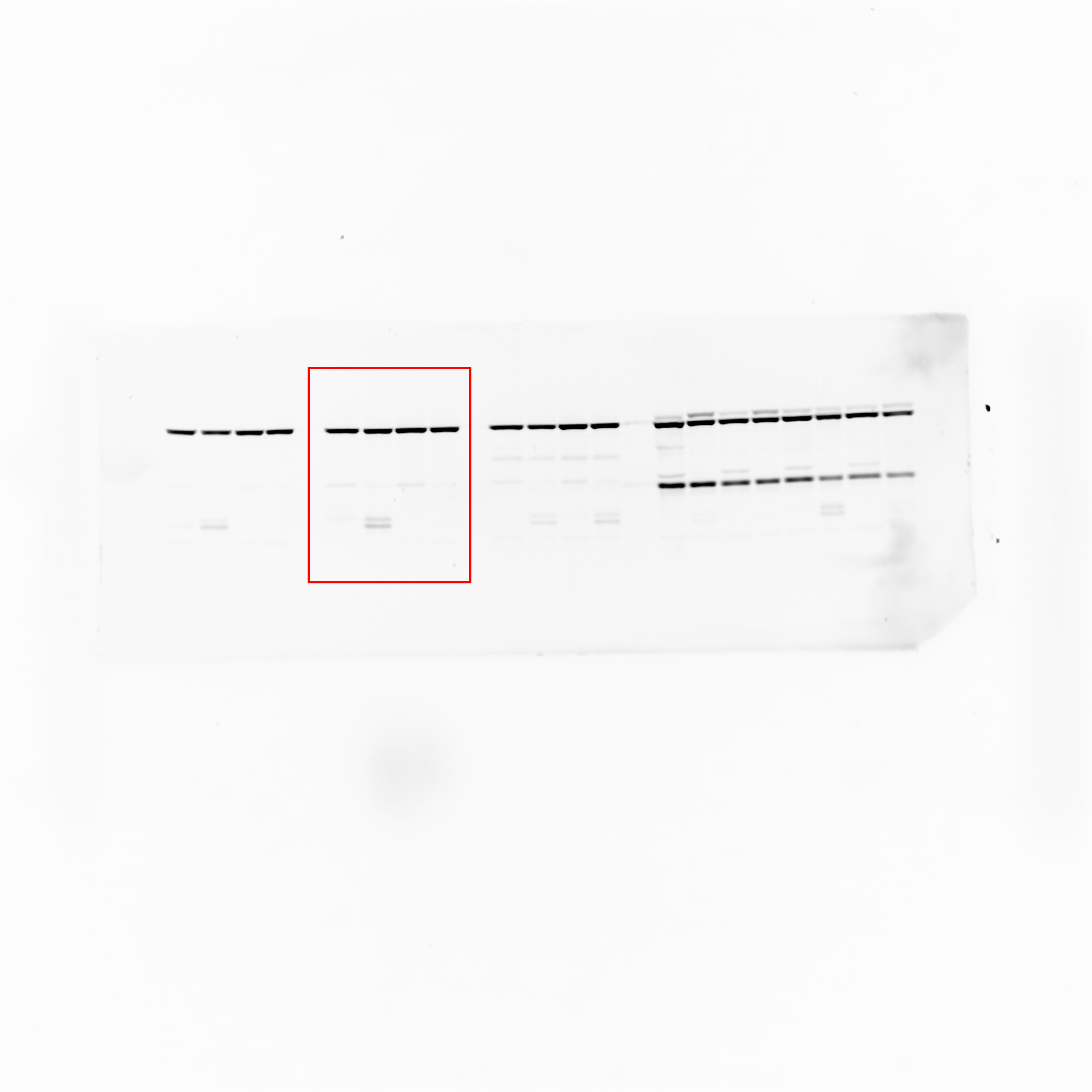

Supplement: Figure 3—source data 1. [file elife-78923-fig3-data1.zip › Figure 3-source data 1/Figure 3a_TMR in-gel fluorescence_annotated.tif]

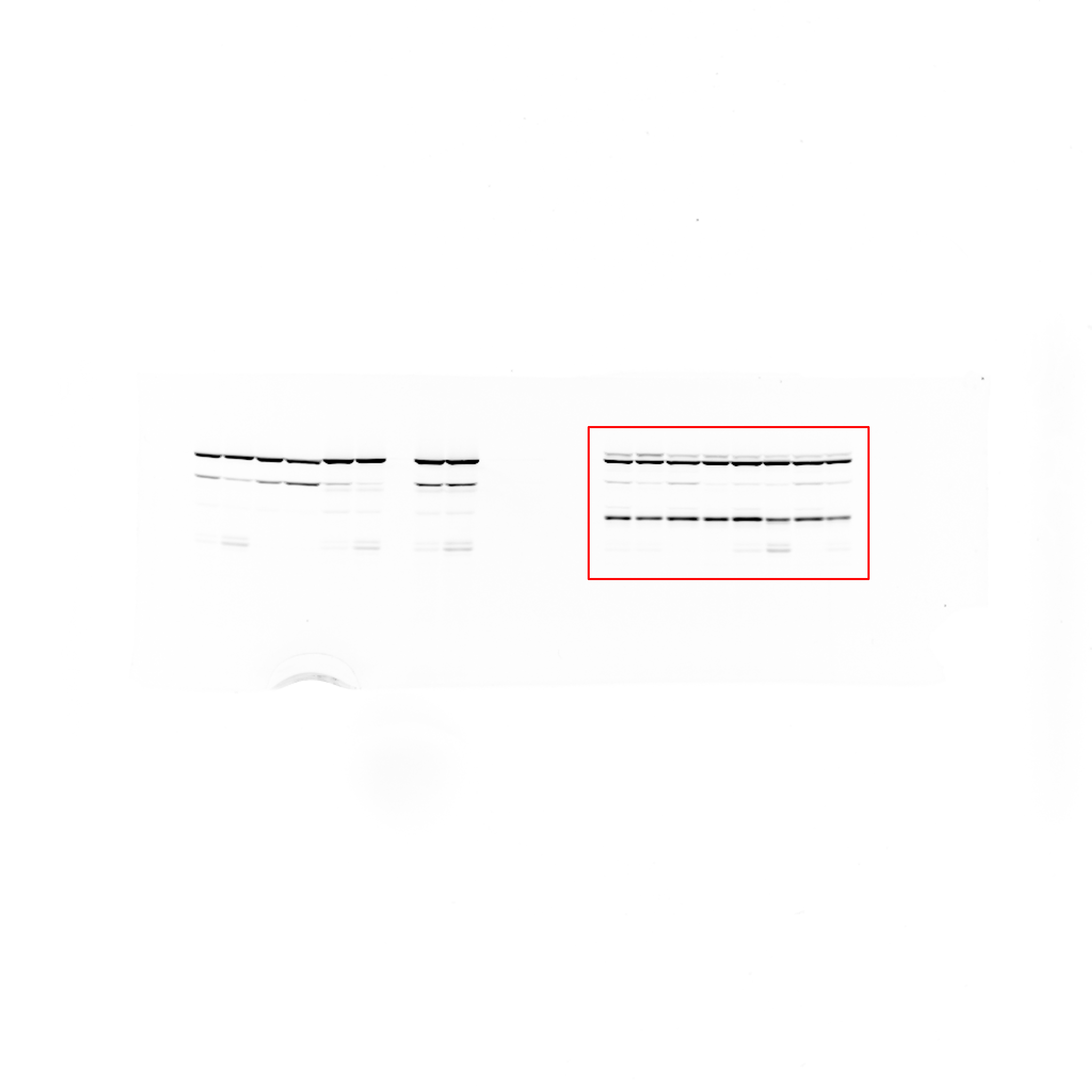

Supplement: Figure 3—source data 1. [file elife-78923-fig3-data1.zip › Figure 3-source data 1/Figure 3g_TMR in-gel fluorescence_annotated.tif]

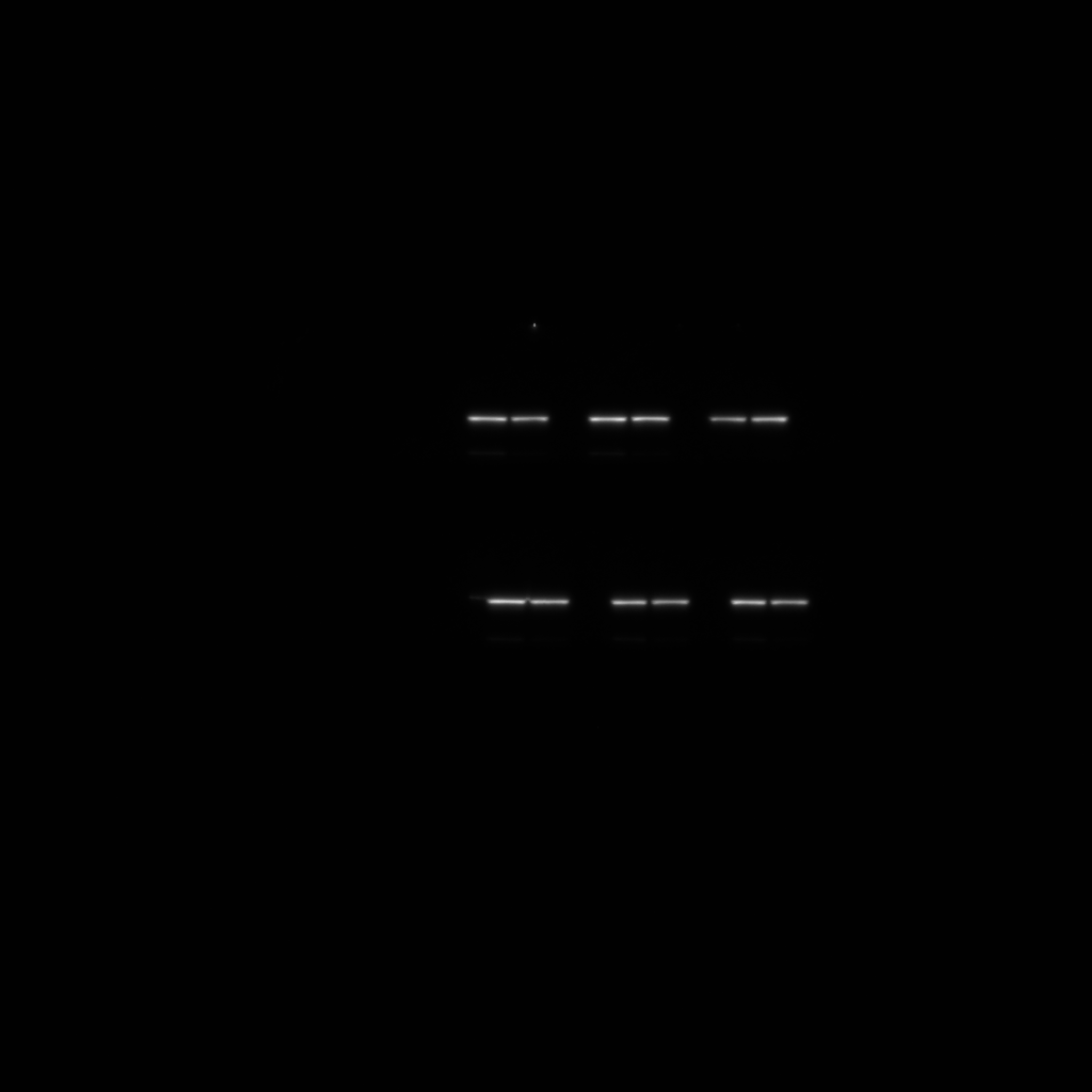

Supplement: Figure 3—source data 1. [file elife-78923-fig3-data1.zip › Figure 3-source data 1/Figure 3c_Calnexin blot_raw.Tif]

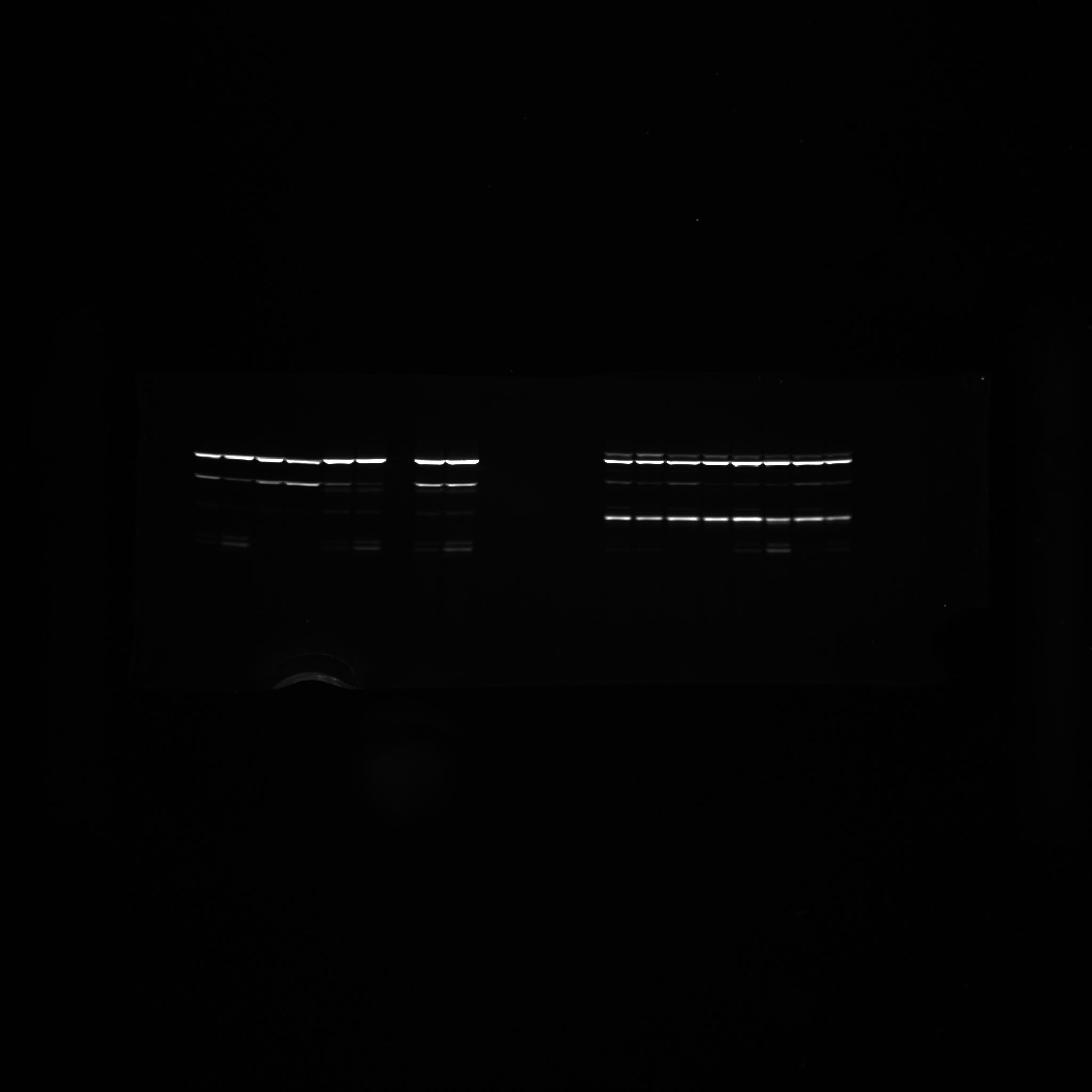

Supplement: Figure 3—source data 1. [file elife-78923-fig3-data1.zip › Figure 3-source data 1/Figure 3g_TMR in-gel fluorescence_raw.Tif]

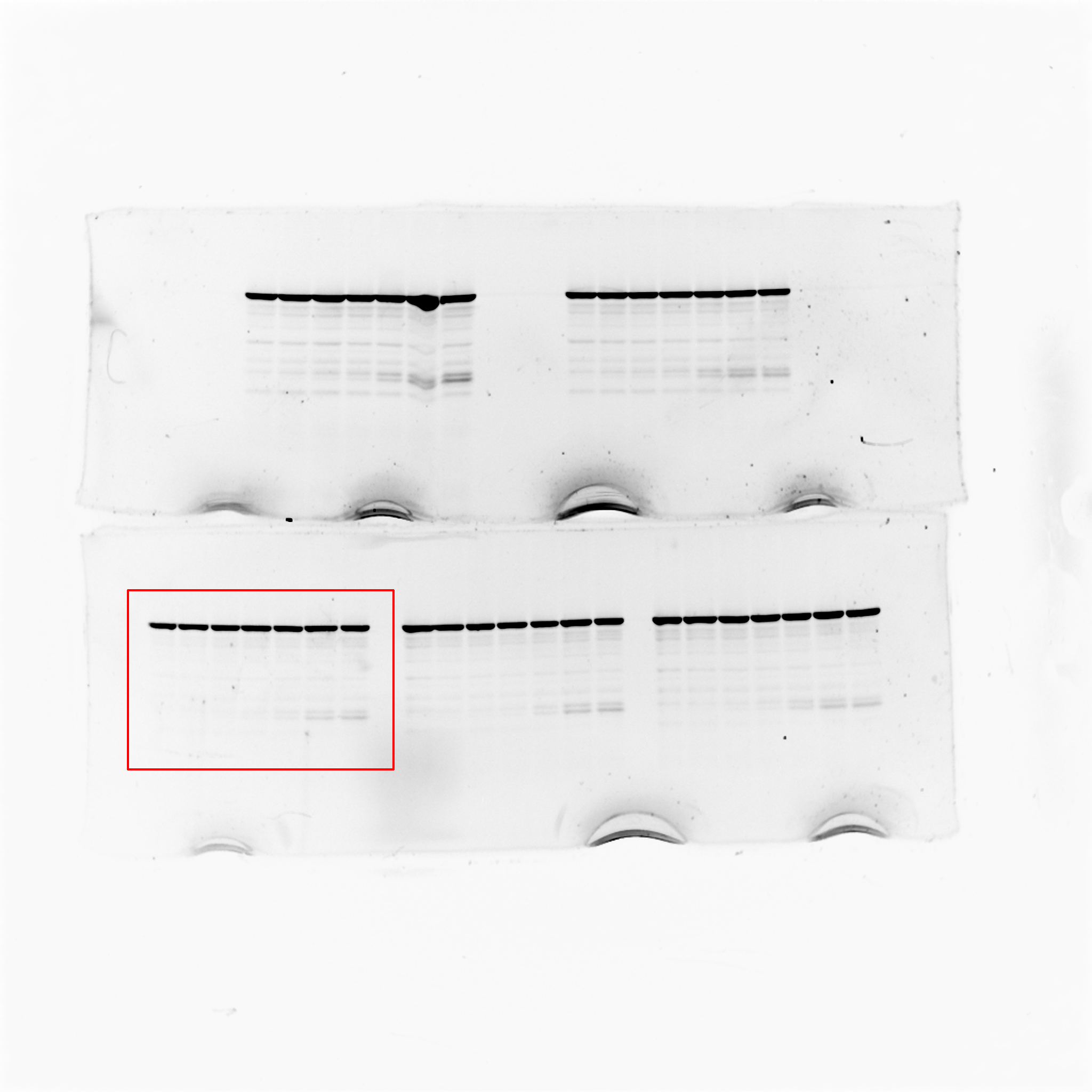

Supplement: Figure 4—source data 1. [file elife-78923-fig4-data1.zip › Figure 4-source data 1/Figure 4b_TMR in-gel fluorescence_annotated.tif]

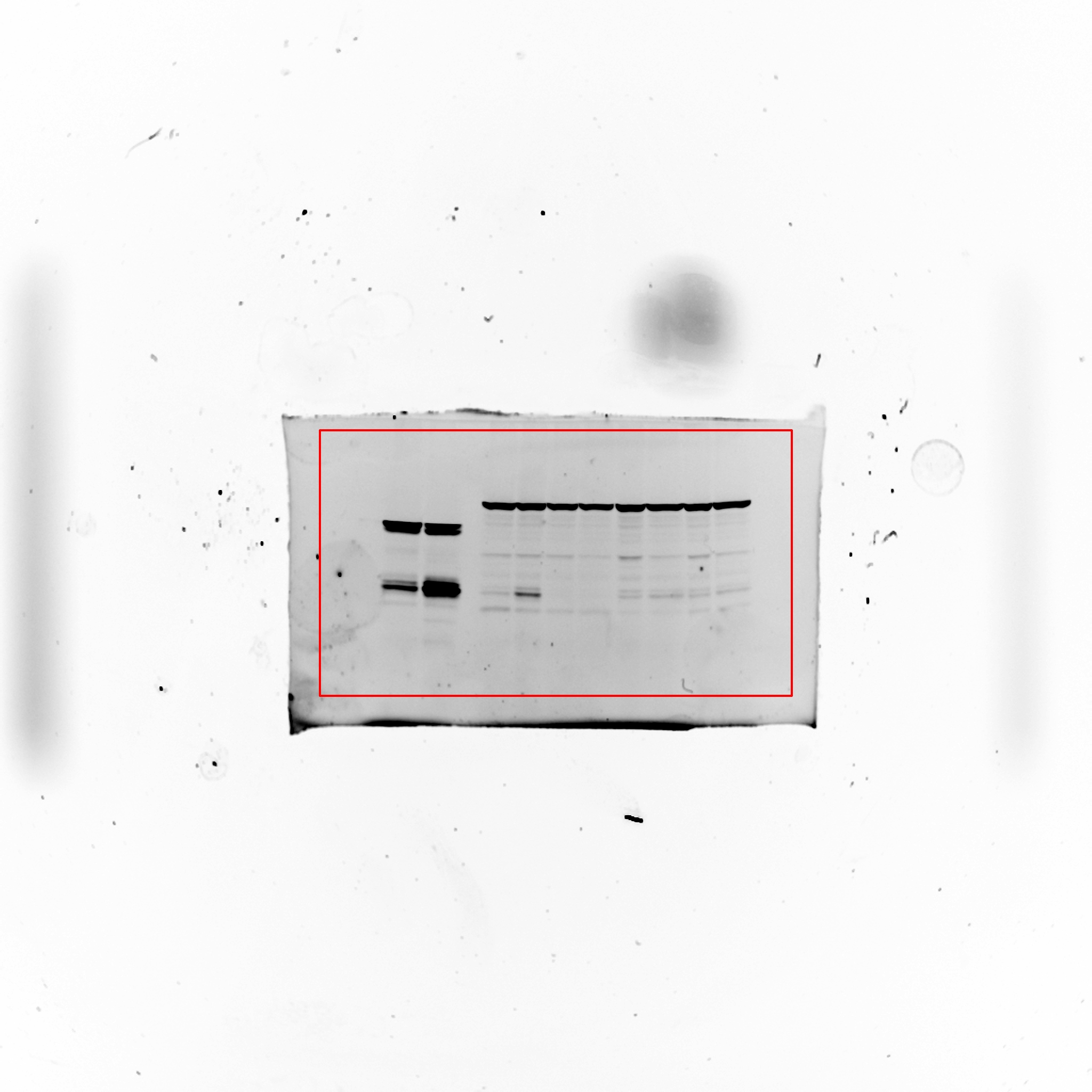

Supplement: Figure 4—source data 1. [file elife-78923-fig4-data1.zip › Figure 4-source data 1/Figure 4a_TMR in-gel fluorescence_annotated.tif]

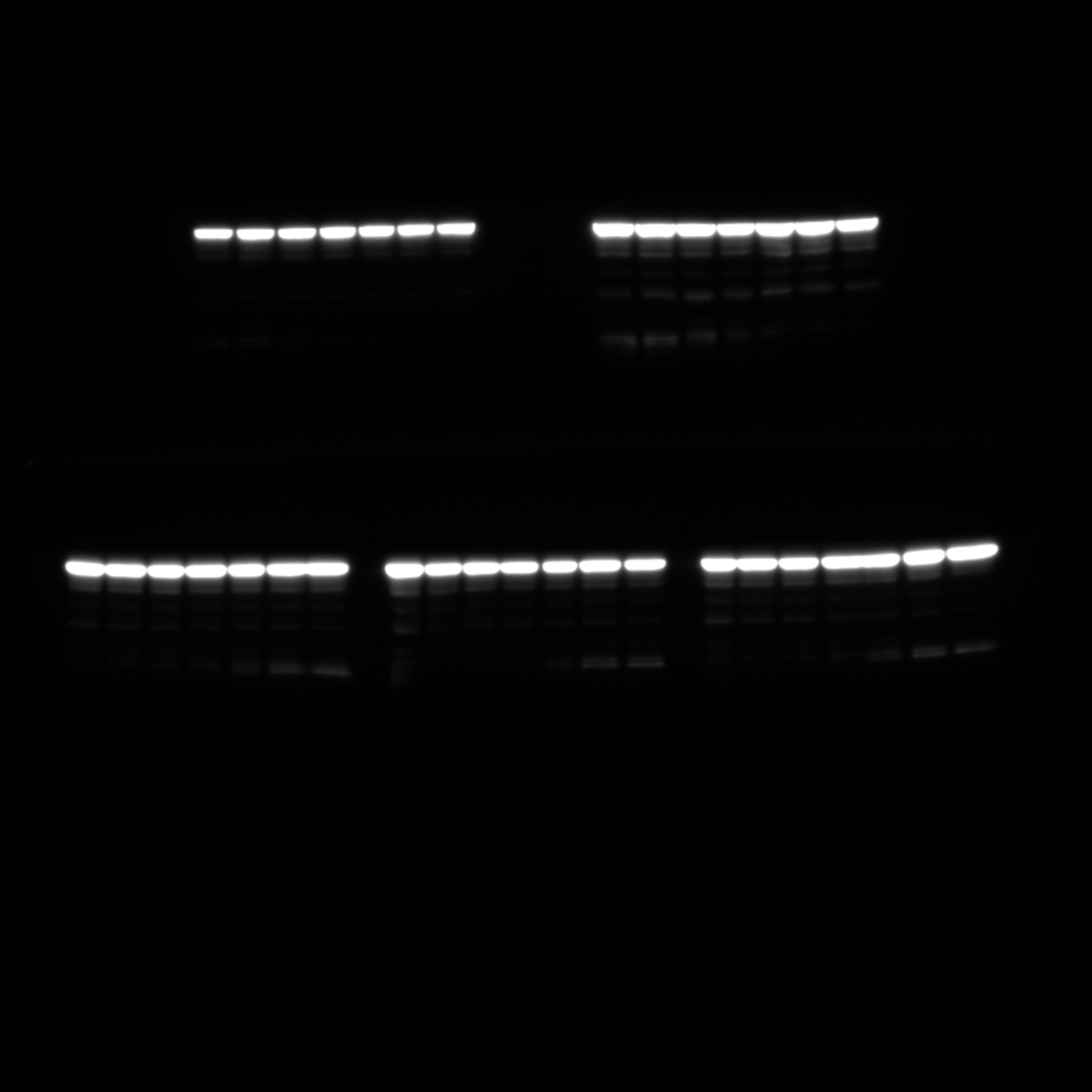

Supplement: Figure 4—source data 1. [file elife-78923-fig4-data1.zip › Figure 4-source data 1/Figure 4b_HaloTag blot_raw.Tif]

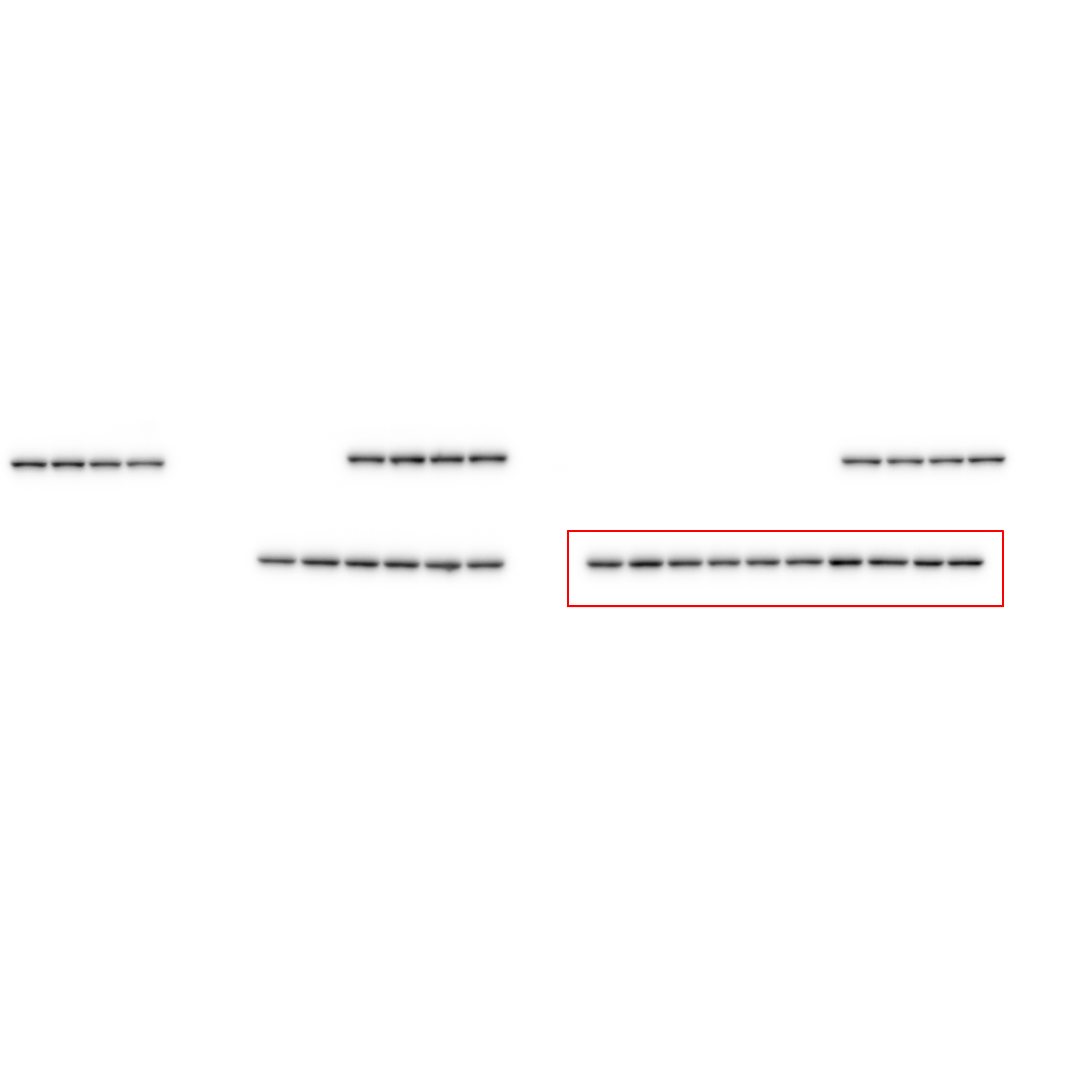

Supplement: Figure 4—source data 1. [file elife-78923-fig4-data1.zip › Figure 4-source data 1/Figure 4a_Hsp90 blot_annotated.tif]

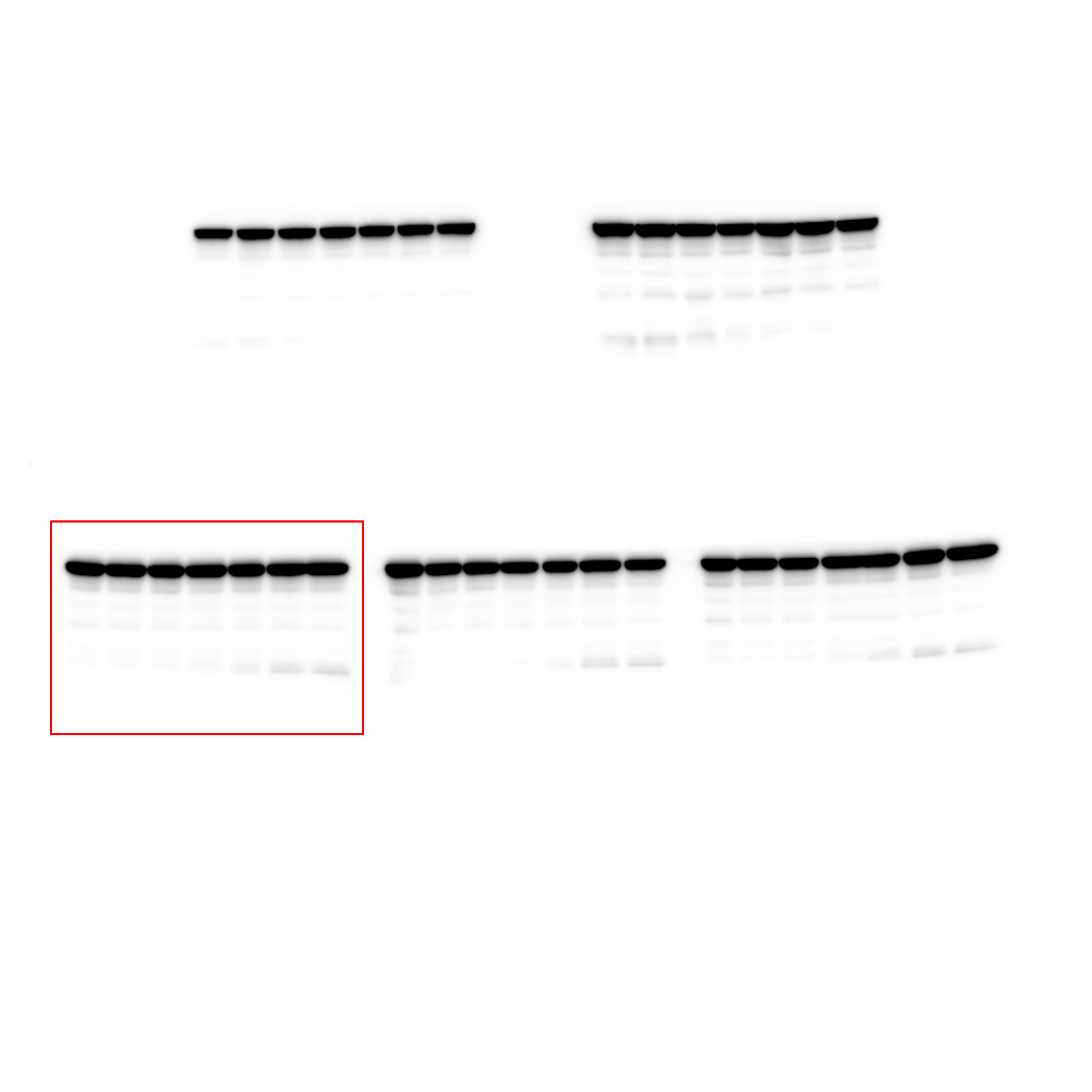

Supplement: Figure 4—source data 1. [file elife-78923-fig4-data1.zip › Figure 4-source data 1/Figure 4b_HaloTag blot_annotated.tif]

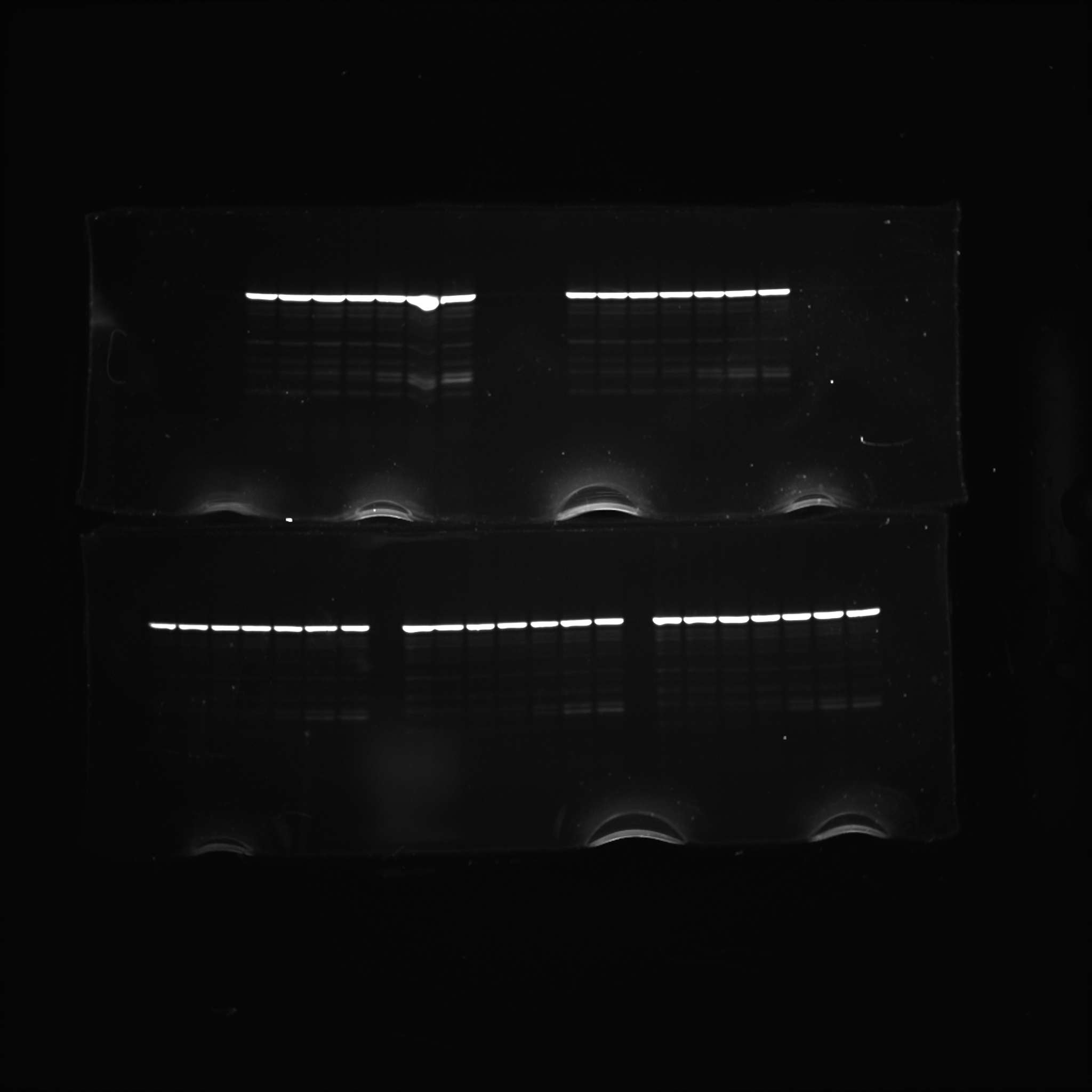

Supplement: Figure 4—source data 1. [file elife-78923-fig4-data1.zip › Figure 4-source data 1/Figure 4b_TMR in-gel fluorescence_raw.TIF]

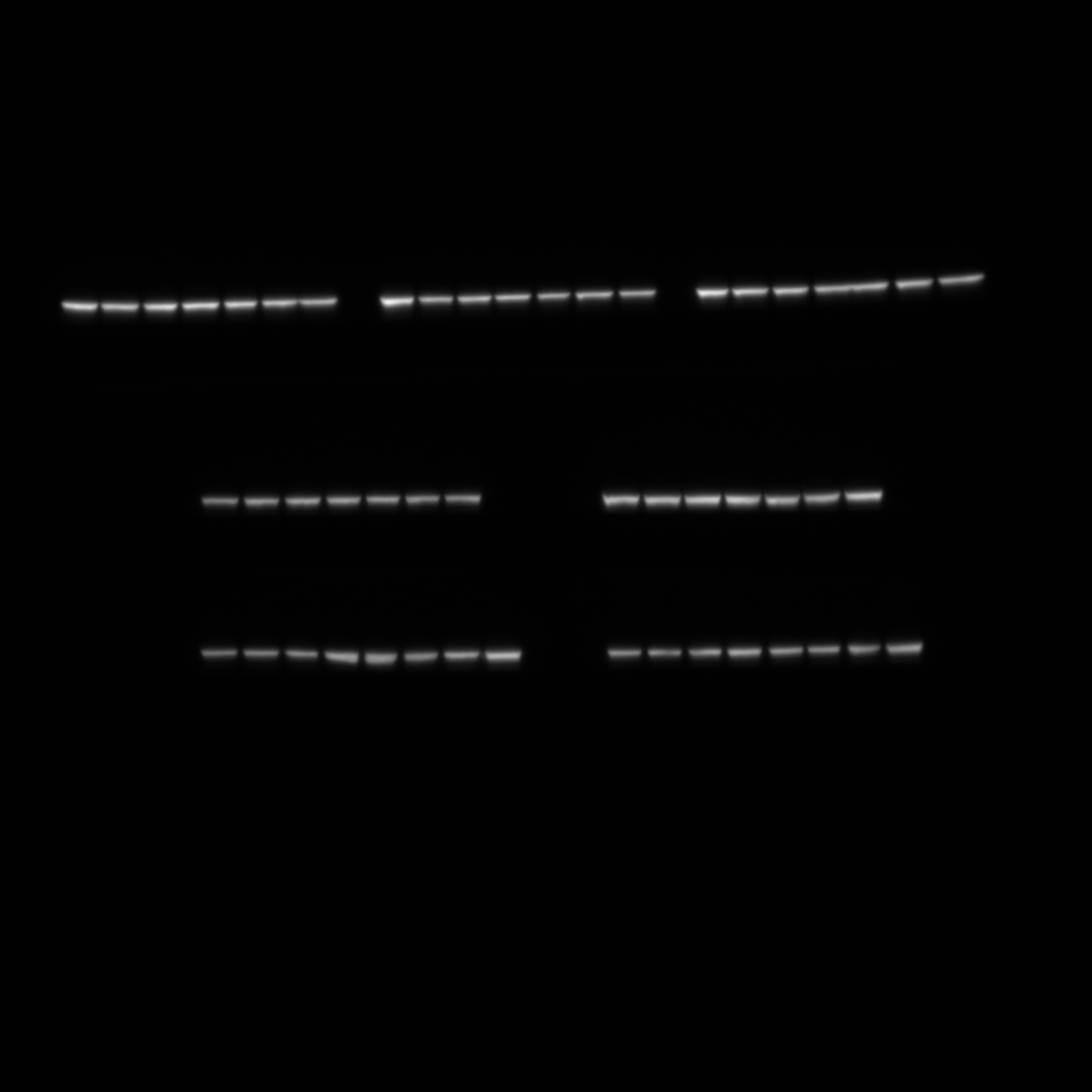

Supplement: Figure 4—source data 1. [file elife-78923-fig4-data1.zip › Figure 4-source data 1/Figure 4b_Hsp90 blot_raw.Tif]

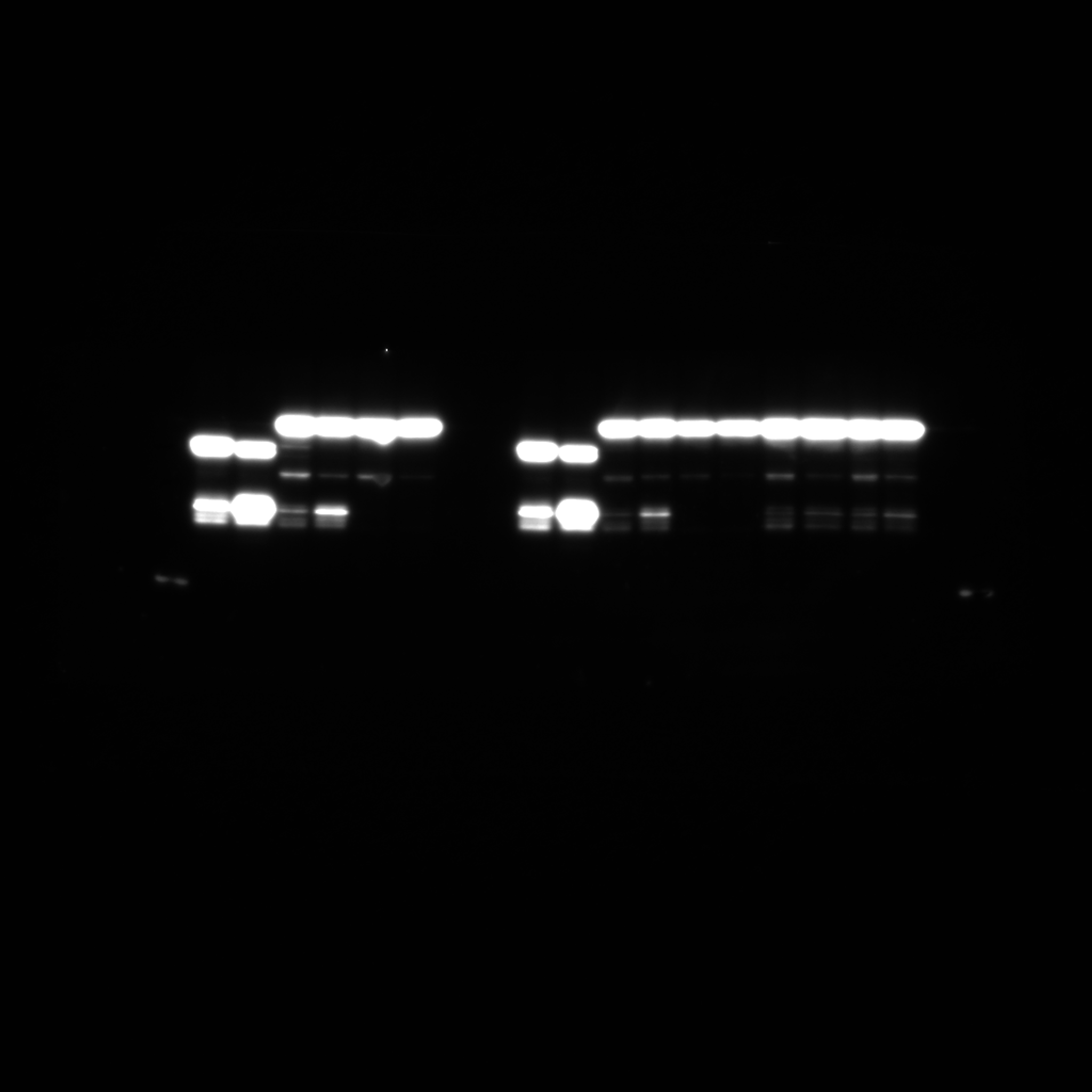

Supplement: Figure 4—source data 1. [file elife-78923-fig4-data1.zip › Figure 4-source data 1/Figure 4a_HaloTag blot_raw.Tif]

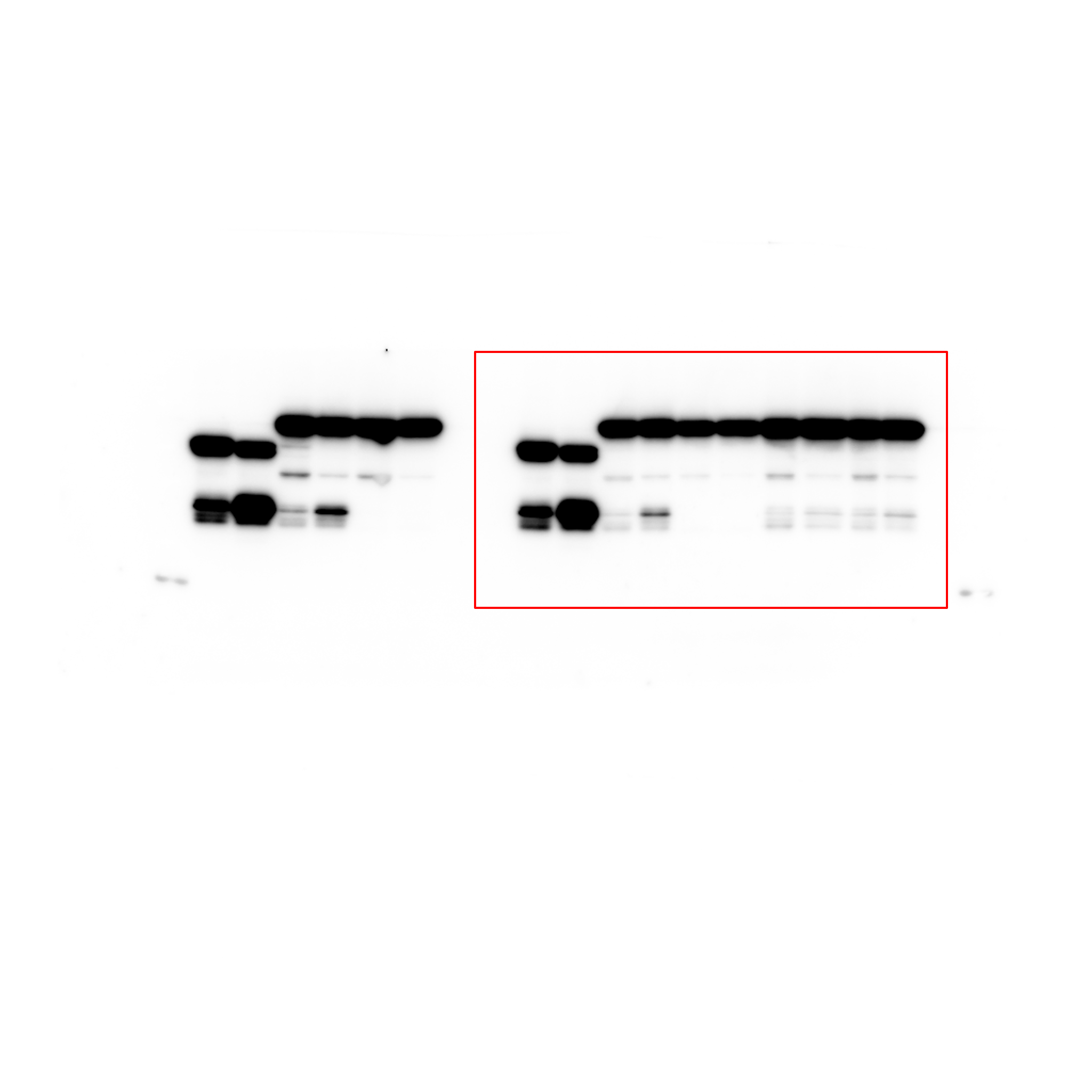

Supplement: Figure 4—source data 1. [file elife-78923-fig4-data1.zip › Figure 4-source data 1/Figure 4a_HaloTag blot_annotated.tif]

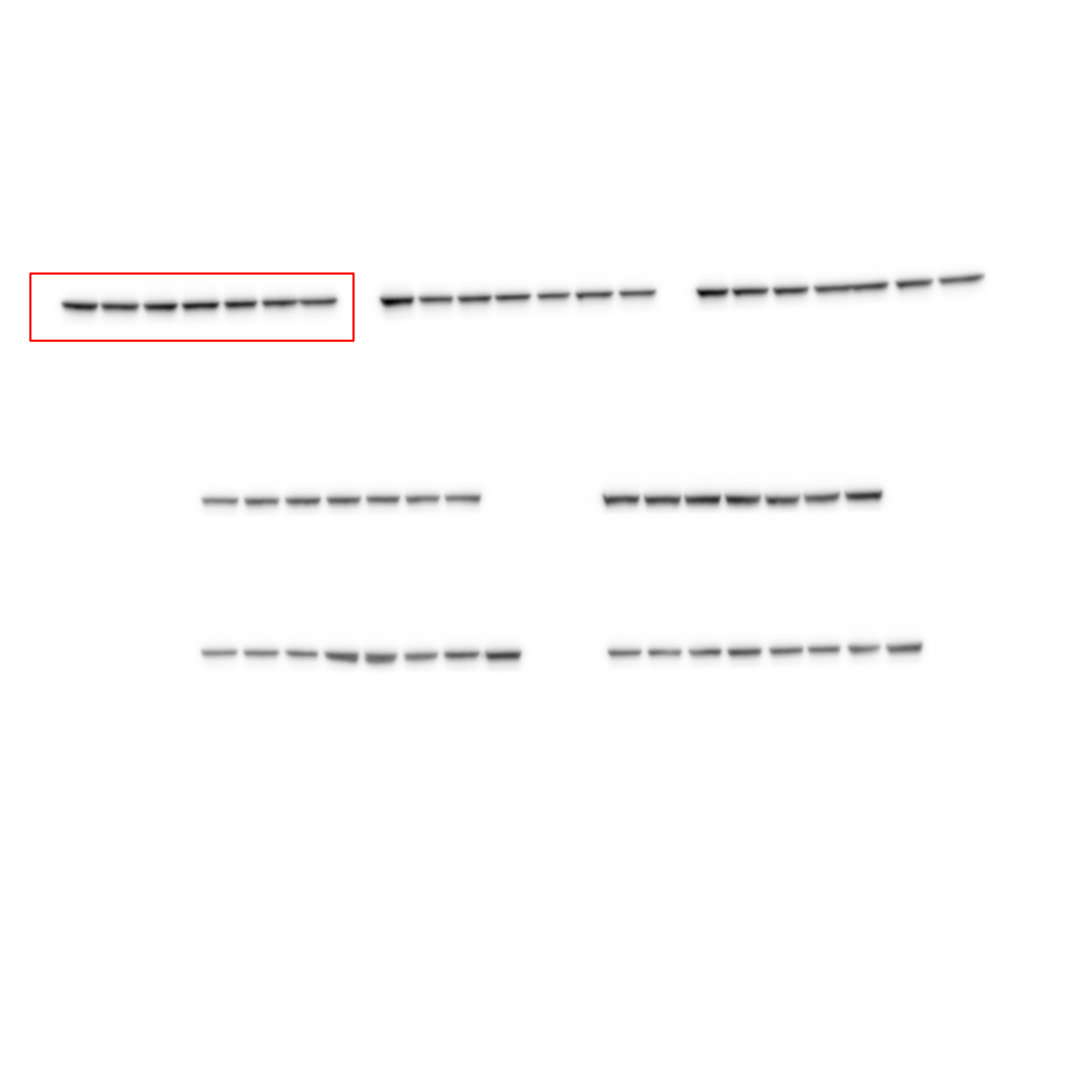

Supplement: Figure 4—source data 1. [file elife-78923-fig4-data1.zip › Figure 4-source data 1/Figure 4b_Hsp90 blot_annotated.tif]

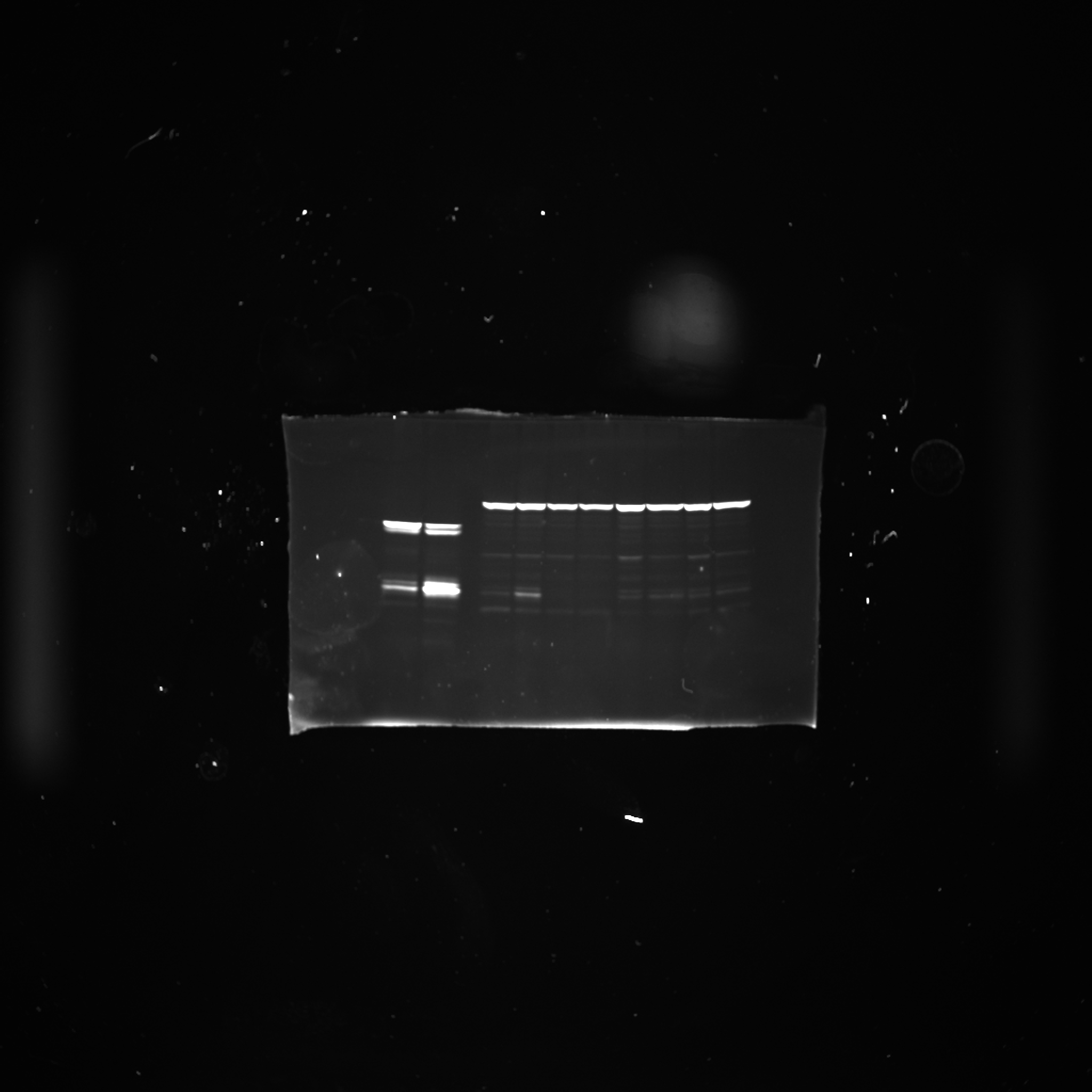

Supplement: Figure 4—source data 1. [file elife-78923-fig4-data1.zip › Figure 4-source data 1/Figure 4a_TMR in-gel fluorescence_raw.TIF]
